# Cytomegalovirus microRNAs Facilitate Persistent Virus Infection in Salivary Glands

**DOI:** 10.1371/journal.ppat.1001150

**Published:** 2010-10-14

**Authors:** Lars Dölken, Astrid Krmpotic, Sheila Kothe, Lee Tuddenham, Mélanie Tanguy, Lisa Marcinowski, Zsolt Ruzsics, Naama Elefant, Yael Altuvia, Hanah Margalit, Ulrich H. Koszinowski, Stipan Jonjic, Sébastien Pfeffer

**Affiliations:** 1 Max von Pettenkofer-Institute, Ludwig-Maximilians-University Munich, Munich, Germany; 2 Department of Histology and Embryology, Faculty of Medicine University of Rijeka, Rijeka, Croatia; 3 Architecture et Réactivité de l'ARN, Université de Strasbourg, Institut de Biologie Moléculaire et Cellulaire du CNRS, Strasbourg, France; 4 Department of Microbiology and Molecular Genetics, Faculty of Medicine, The Hebrew University, Jerusalem, Israel; Oregon Health and Science University, United States of America

## Abstract

Micro (mi)RNAs are small non-coding RNAs that regulate the expression of their targets' messenger RNAs through both translational inhibition and regulation of target RNA stability. Recently, a number of viruses, particularly of the herpesvirus family, have been shown to express their own miRNAs to control both viral and cellular transcripts. Although some targets of viral miRNAs are known, their function in a physiologically relevant infection remains to be elucidated. As such, no *in vivo* phenotype of a viral miRNA knock-out mutant has been described so far. Here, we report on the first functional phenotype of a miRNA knock-out virus *in vivo*. During subacute infection of a mutant mouse cytomegalovirus lacking two viral miRNAs, virus production is selectively reduced in salivary glands, an organ essential for virus persistence and horizontal transmission. This phenotype depends on several parameters including viral load and mouse genetic background, and is abolished by combined but not single depletion of natural killer (NK) and CD4^+^ T cells. Together, our results point towards a miRNA-based immunoevasion mechanism important for long-term virus persistence.

## Author Summary

The recent discovery of miRNAs of viral origin has dramatically changed our view on virus-host interaction. Viral miRNAs have been shown to regulate genes of both cellular and viral origin, contributing to a favorable environment for the virus. However, the real importance of virus-encoded miRNAs during infection of their hosts remains elusive. In this manuscript, we report on the first functional phenotype of a miRNA knock-out mutant of the mouse cytomegalovirus *in vivo*. We show that the mutant virus is attenuated specifically in the salivary glands of infected mice, an organ essential for long-term persistence of the virus and host-to-host spread. Interestingly, this attenuation revealed a striking dependence on genetic background of the mice under study. Only combined depletion of natural killer and T cells abolished the phenotype. These results indicate that, by regulating the immune system, viral miRNAs may play an important role in an efficient persistent infection.

## Introduction

The human cytomegalovirus (HCMV), a member of the β-herpesvirus family, is an important pathogen in immunocompromised patients and the leading cause of congenital birth defects with about 1/1,000 newborns affected [1]. After primary infection, herpesviruses establish a life-long latent infection, leaving the infected host at risk of subsequent reactivation and disease. During their co-evolution with their hosts, cytomegaloviruses have encountered a broad array of immune defense mechanisms, and have thus developed multiple strategies to counteract them (reviewed in [2]). Recently, both viral and cellular miRNAs have been identified as new players in the complex interaction between viruses and their hosts, providing interesting new candidates for targets of urgently needed antiviral drugs (for review see [3]). These small, ∼22 nucleotides long non-coding RNAs regulate the expression of their targets through both translational inhibition and regulation of target RNA stability (for review see [4]). While a single miRNA can regulate the expression of a large number of target genes, the extent of regulation of protein levels usually does not exceed two to three fold [5], [6]. Although a number of viruses (particularly of the herpesvirus family) have been shown to express miRNAs during productive infection and latency [3], the function of viral miRNAs in a physiologically relevant infection remains to be elucidated. Sullivan *et al.* showed that a miRNA mutant murine polyomavirus was not impaired during *in vivo* infection [7], and thus no phenotype of a viral miRNA knock-out mutant has been described so far. Here, we report on the first functional phenotype of a mouse cytomegalovirus (MCMV) lacking two miRNAs. During subacute infection, miRNA mutant virus production was selectively reduced in salivary glands, the major source of persistent CMV infection and virus spread from host-to-host [8], [9]. This phenotype depended on several parameters including viral load and mouse genetic background, and was abolished by combined depletion of natural killer (NK) and CD4^+^ T cells. Together, our results point towards a miRNA-based immunoevasion mechanism in an organ essential for long-term virus persistence and host-to-host transmission.

## Results/Discussion

### Generation of mutant viruses

**Figure 1:**
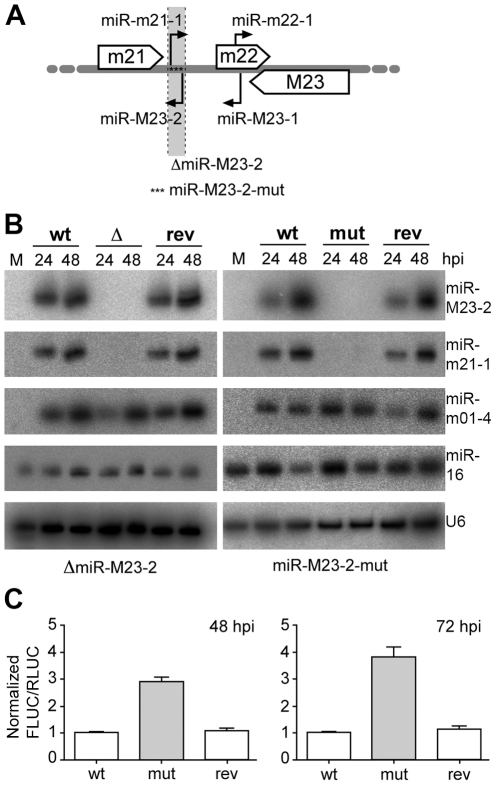
Obtention and characterization of miRNA mutant MCMV. **A.** Schematic illustration of MCMV miRNAs expressed from the m21/m22/M23 MCMV miRNA cluster. Open arrows indicate the coding regions of the m21, m22 and M23 genes. Black arrows indicate pre-miRNAs and their orientation. The region deleted and replaced with stuffer DNA in the ΔmiR-M23-2 mutant MCMV is indicated (light grey and dotted lines), whereas asterisks indicate the point mutations in the miR-M23-2-mut mutant. **B.** Expression levels of miR-M23-2 and miR-m21-1 in NIH-3T3 fibroblasts infected at an MOI of 10 for 24 and 48 h with either wild type (wt) MCMV, MCMV-ΔmiR-M23-2 (Δ) and its revertant (rev) or wt MCMV, MCMV-miR-M23-2-mut (mut) and its corresponding revertant (rev) were determined by northern blot. The viral miRNA mcmv-miR-m01-4, the cellular miR-16 and the snRNA U6 were used as controls. M, mock. **C.** MiR-M23-2 is able to repress target gene expression late in infection. NIH-3T3 cells were infected either with wt MCMV, MCMV-miR-M23-2-mut or its revertant. 24 hpi cells were transfected with a dual luciferase reporter construct containing a perfect match for miR-M23-2 in the 3′-UTR of firefly luciferase (FLUC). *Renilla* luciferase (RLUC) activity was used as a transfection control. Luciferase activity was measured at 48 and 72 hpi.

During productive lytic infection a variety of virally encoded miRNAs are expressed by both HCMV (11 miRNAs) and MCMV (18 miRNAs), which accumulate to high levels throughout the course of infection [10], [11], [12], [13]. This was seen for a number of different cell types upon HCMV and MCMV infection. In MCMV infected fibroblasts, viral miRNAs constitute as much as two thirds of the total miRNA pool at three days post infection (dpi) as assessed by small RNA cloning [12]. At this time-point, two viral miRNAs, namely miR-M23-2 and miR-m21-1, together constituted ∼25% of the overall miRNAs in small RNA libraries. Both miRNAs belong to the m21/m22/M23 miRNA cluster and consist of two pairs of pre-miRNAs, which are expressed antisense to each other at the same genomic locus (Fig. 1A). Of note, this peculiar localization does not hinder their relative expression. We recently reported on an MCMV knock-out mutant lacking both of these viral miRNAs [11]. In this mutant (ΔmiR-M23-2), pre-miR-M23-2 was replaced by 276 nt of stuffer DNA resulting in the knock-out of both miR-M23-2 and miR-m21-1. We now constructed a second independent mutant (miR-M23-2-mut) by traceless mutagenesis [14] inserting 17 point mutations into the miRNA precursor sequence to disrupt hairpin formation and pre-miRNA processing (Fig. 1A). In addition, revertants were created for both viruses (ΔmiR-M23-2-rev and miR-M23-2-mut-rev) by fully restoring the native pre-miRNA locus. Expression of the respective miRNAs was measured by northern blot (Fig. 1B), confirming efficient knock-out and repair of miRNA expression in the mutant and revertant viruses, respectively. Expression of the neighboring genes and miRNAs of the m21/m22/M23 miRNA cluster were quantified by qPCR (for m21 and M23 mRNAs) (Fig. S1A, B) and northern blot (for mcmv-miR-M23-1-3p and miR-m22-1) (Fig. S1C). In both mutants and their respective revertants, no significant effect on expression levels of neighboring transcripts or miRNAs was observed. To demonstrate that miR-M23-2 is functional and can repress luciferase activity of a sensor target during lytic MCMV infection, we created a reporter construct expressing firefly luciferase containing a perfect match binding site for miR-M23-2 in its 3′UTR. Dual luciferase assays demonstrated that miR-M23-2 is functional and can repress luciferase activity of a sensor target during lytic MCMV infection with wild type (wt) MCMV and the revertant virus, but not with miR-M23-2-mut mutant virus (Fig. 1C).

### Both MCMV-ΔmiR-M23-2 and MCMV-miR-M23-2-mut are specifically attenuated in salivary glands during subacute infection

Neither of the two miRNA knock-out mutants showed any attenuation on murine embryonic fibroblasts *in vitro* ([11] and data not shown) indicating that the effect of miR-M23-2 mutation on the virus did not affect viral replication in general. It also confirmed that no second site mutation gravely impaired any essential genes of the mutant viruses. We thus started to infect C57BL/6 and BALB/c mice. While C57BL/6 mice are able to efficiently control acute MCMV infection, BALB/c mice are more susceptible. This is due to the activating NK cell receptor Ly49H, present in C57BL/6 but not BALB/c mice, which directly recognizes the MCMV m157 protein resulting in robust NK cell activation and enhanced virus control [15], [16]. Following three days of infection with either wt MCMV, ΔmiR-M23-2 or its revertant, no difference in virus titers were observed in lungs, spleen or kidney (Fig. S2). The lack of attenuation in any organ of both C57BL/6 and BALB/c at 3 dpi indicates that time might be an important factor to consider when investigating the function of viral miRNAs. As miRNAs are only able to repress *de novo* protein synthesis but have no effect on existing proteins, their effect depends on the decay of existing proteins unless their targets are significantly induced upon infection. Relevant levels of most viral miRNAs are probably not reached before 12 to 24 hours post infection (hpi), and it might take even longer until they are able to recruit significant amounts of their target RNAs to RISC complexes. As first viral progeny are released from infected cells as early as 24 hpi [17], viral miRNAs probably do not have sufficient time to regulate the majority of their targets during early stages of infection.

**Figure 2:**
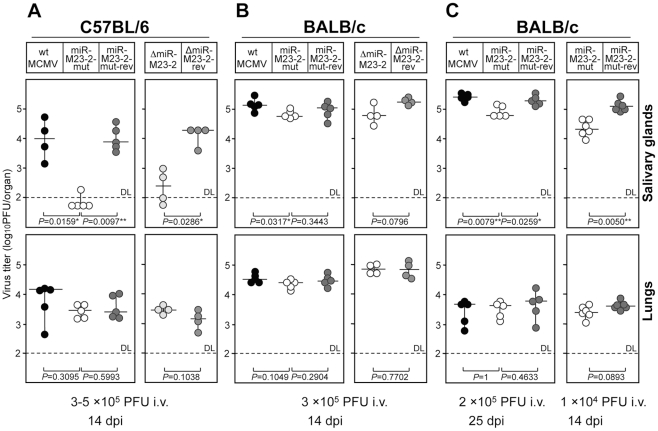
Phenotype of miRNA mutant and revertant MCMV in salivary glands and lungs. **A.** C57BL/6 mice were injected intravenously (i.v.) with 3×10^5^ PFU (left panel) or 1×10^5^ PFU (right panel) of indicated viruses. Virus titers in salivary glands and lungs were determined 14 days post infection. There were significant differences in virus titers in salivary glands between the groups of mice infected with miR-M23-2-mut and wt MCMV, miR-M23-2-mut and miR-M23-2-mut-rev, as well as between the groups of mice infected with ΔmiR-M23-2 and ΔmiR-M23-2-rev. **B.** BALB/c mice were injected i.v. with 3×10^5^ PFU of indicated viruses. Virus titers in salivary glands and lungs were determined 14 dpi. **C.** BALB/c mice were injected i.v. with 2×10^5^ PFU (left panel) or 1×10^4^ PFU (right panel) of indicated viruses. Virus titers in salivary glands and lungs were determined 25 days (left panel) or 14 days (right panel) post infection. There were significant differences in virus titers in salivary glands between the groups of mice infected with miR-M23-2-mut and wt MCMV, miR-M23-2-mut and miR-M23-2-mut-rev (left panel), as well as between the groups of mice infected with miR-M23-2-mut and wt MCMV, and miR-M23-2-mut and miR-M23-2-mut-rev (right panel). Titers in organs of individual mice (circles) and median values (horizontal bars) are shown. DL  =  detection limit; * p<0.05; ** p<0.01.

We therefore studied mice at 14 dpi. At this time, the immune system already controls the virus in most organs, except in lungs and salivary glands. Interestingly, infection with both mutants resulted in an attenuation of ∼100-fold in salivary glands of C57BL/6 mice. However, only minimal attenuation (<2-fold) was detectable in salivary glands of BALB/c mice (Fig. 2A and B). In contrast, all five viruses under study replicated to similar titers in lungs in both strains. The complete lack of any attenuation in lungs of C57BL/6 mice was surprising, but confirmed that all mice had been infected with an equal virus load. As this phenotype was comparable for both mutants and completely lifted by the two revertants, we can be confident that the knock-out of the two miRNAs was responsible for the observed attenuation. As a consequence, only the second set of mutants (MCMV-miR-M23-2-mut and its revertant) were used in further experiments. The lack of attenuation of the miRNA knock-out mutants at 3 dpi as well as the selective attenuation in C57BL/6 but not BALB/c mice at 14 dpi following intravenous (i.v.) infection, argues for a specific function of these two MCMV miRNAs in supporting persistent infection in salivary glands, and against an impairment of virus spread to this site. A few MCMV genes have been identified to be specifically required to maintain spread as well as persistent infection in salivary glands, but their mode of action has remained questionable [18].

### Level of attenuation is dependent on mouse strain and viral load

**Figure 3:**
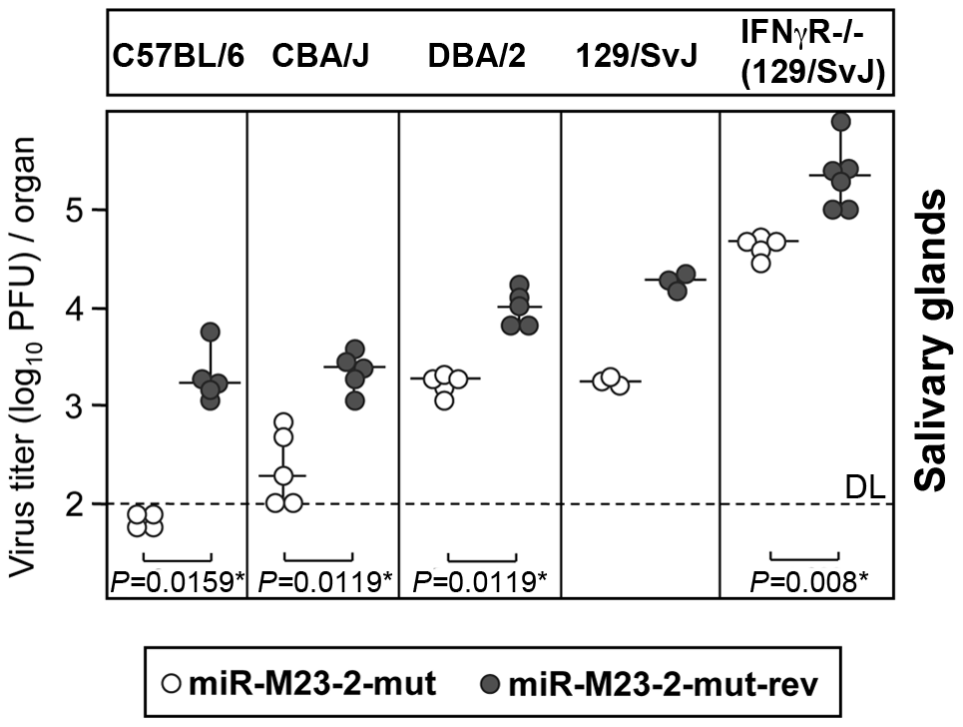
Attenuation of miRNA mutant MCMV in various mouse genetic backgrounds. C57BL/6, CBA/J, DBA/2, 129/SvJ. and 129/SvJ IFNγR^−/−^ mice were i.p. injected with 2×10^5^ PFU of MCMV-miR-M23-2-mut or MCMV-miR-M23-2-mut-rev. Virus titers in salivary glands were determined 14 dpi. Titers in organs of individual mice (circles) and median values (horizontal bars) are shown. There were significant differences in virus titers in salivary glands between mice infected with MCMV-miR-M23-2-mut and MCMV-miR-M23-2-mut-rev for C57BL/6, CBA/J, DBA/2 and 129.SvJ IFNgR^−/−^ mice. DL  =  detection limit; * p<0.05; ** p<0.01.

In order to test whether the miRNA knock-out mutant would require more time for attenuation to become apparent in BALB/c mice, we infected BALB/c mice for 25 days. While at 25 dpi virus titers in lungs had significantly dropped compared to 14 dpi, virus titers in salivary glands were very similar to those seen after 14 days. Only a very small, yet significant attenuation by ∼2-fold was observed in salivary glands (Fig. 2C), while no attenuation was observed in lungs. This clearly less pronounced attenuation of mutant virus in salivary glands in BALB/c mice could be the consequence of the inability of the host immune system to overcome high virus load in this strain, due to the less stringent innate immune control, as compared to C57BL/6 mice. Indeed, virus titers observed in salivary glands of BALB/c mice at both 14 and 25 dpi were significantly higher than in C57BL/6 mice. To test whether viral load in salivary glands had any effect on the observed phenotype, we repeated the infection of BALB/c mice with 20-fold less virus. Remarkably, virus dose reduction now resulted in a significantly greater attenuation (∼5 fold) of the mutant in salivary glands at 14 dpi (Fig. 2C). The attenuation of the mutant virus could also be seen in BALB/c mice infected intraperitoneally with 5×10^4^ PFU (Fig. S3). To further characterize the effects of host genetics on the observed phenotype, we tested three other mouse strains with different susceptibility to MCMV infection (CBA/J, DBA/2 and 129/SvJ mice). In addition, we also included 129/SvJ.IFNγR^−/−^ mice [19], which lack the IFNγ receptor and are thus even more susceptible to MCMV infection than their parental strain. Interestingly, miR-M23-2-mut was attenuated in salivary glands of all four strains at 14 dpi (Fig. 3). Despite very high viral load, attenuation was also observed in 129/SvJ.IFNγR^−/−^ mice. Therefore, both host genetics and viral load are contributing factors involved in attenuation of the miRNA knock-out mutant in salivary glands.

Notably, control of MCMV in salivary glands is completely different than in any other tissue. The virus counteracts host defenses in this sentinel organ by means that are still not fully understood. Virus persistence in salivary gland tissues appears mainly to reflect the situation in acinar glandular epithelial cells. In these cells, MCMV replicates to high titers with a distinct morphogenesis and without causing gross tissue damage [20]. Infectious particles are stored and secreted from large cytoplasmic vacuoles, orientated towards excretion ducts, filled with high numbers (∼1,000) of virions [21]. Interestingly, for unknown reasons, virus isolated from salivary glands of naive mice at 14 dpi is also several fold more virulent than virus coming from any other organ or from cell culture. This gain in virulence is lost after a single round of replication in tissue culture [22]. As MCMV infection in salivary glands results in prolonged production of virus with only minimal cytopathic effects, viral miRNAs most likely gain more time to affect target protein levels in this setting. In contrast, severe tissue damage in lungs caused by prolonged and uncontrolled MCMV infection is the main cause of death in animals infected with a lethal dose of MCMV [23]. We speculate that the lack of attenuation in lungs after 14 and 25 days of infection may be explained by the dominance of lytic virus production over virus persistence. However, selective attenuation of the miRNA knock-out mutant in salivary glands may also reflect viral miRNAs targeting immune control mechanisms of particular importance in this organ or miRNA-mediated effects which, in other organs, are exerted by other viral genes.

### Attenuation of MCMV-miR-M23-2-mut in salivary glands is reverted by combined but not single depletion of NK- and CD4-T-cells

**Figure 4:**
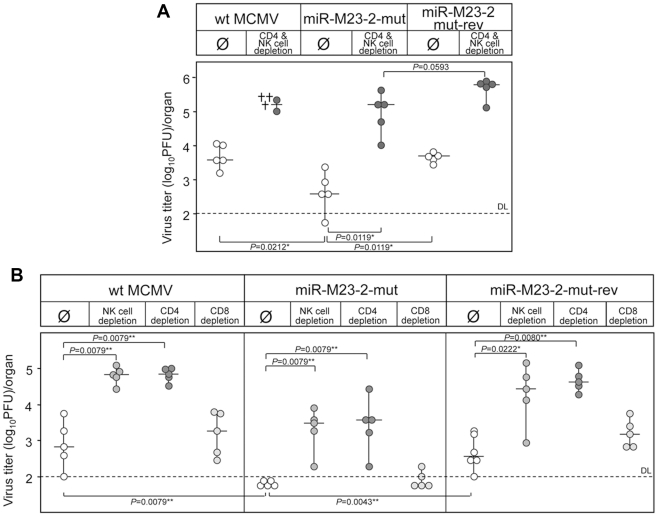
Reversion of the miRNA mutant phenotype by immune cells depletion. **A.** C57BL/6 mice depleted for both NK and CD4^+^ T-cells or mock treated were i.v. injected with 3×10^5^ PFU of either wt MCMV, MCMV-miR-M23-2-mut or its revertant. Virus titers in salivary glands were determined 14 dpi. Differences in virus titers were significant between groups of undepleted mice infected with miR-M23-2-mut and wt MCMV, as well as with miR-M23-2-mut and miR-M23-2-mut-rev. Depletion of CD4^+^ T cells and NK cells resulted in significant increase in the titer of miR-M23-2-mut as well as miR-M23-2-mut-rev. Differences in virus titers between groups of depleted animals infected with miR-M23-2-mut and miR-M23-2-mut-rev were not significant. The crosses for the group of wt MCMV infected and CD4 and NK cell depleted group indicate animals that died a day before virus determination. **B.** C57BL/6 mice, undepleted or depleted of either CD4^+^ T cells, CD8^+^ T cells or NK-cells, were i.p. injected with 2×10^5^ PFU of wt MCMV, MCMV-miR-M23-2-mut or its revertant. Virus titers in salivary glands were determined 14 dpi. Titers in organs of individual mice (circles) and median values (horizontal bars) are shown. DL  =  detection limit; * p<0.05; ** p<0.01.

In salivary glands MCMV infection is almost completely resistant to immunological control by CD8^+^ T cells. Only the concerted action of CD4^+^ T cells and cells with a natural killer (NK) cell-like phenotype finally results in termination of productive infection after many weeks or even months of infection [8], [9]. As such, CD4^+^ T cell deficient mice establish persistent infection for many months, which is restricted to salivary gland [21]. In addition, cytokine signaling is known to play an important role (reviewed in [18]). How the virus persists for a long time in salivary glands, in spite of fully primed immune control, remains an open question. In order to test whether these two MCMV miRNAs are involved in immunological control in salivary glands, we depleted both NK and CD4^+^ T cells in C57BL/6 mice. While the mutant virus was attenuated without NK- and T-cells depletion, combined depletion resulted in significantly higher virus titers (Fig. 4A). When we performed single depletion experiments for NK, CD4^+^ or CD8^+^ T cells in C57BL/6 mice, the depletion of NK and CD4^+^ T cells but not CD8^+^ T cells resulted in a ∼100-fold increase in virus titers (Fig. 4B). This is consistent with previously published data showing that CD4^+^ cells and NK cells control MCMV infection in salivary glands and prevent virus spread [8]. However, although depletion of NK cells or CD4^+^ T cells resulted in significant increase in titers of miR-M23-2-mut, the differences between equally depleted groups of mice infected with wt MCMV or miR-M23-2-mut-rev still remained significant.

Salivary glands are a privileged site for prolonged CMV replication in spite of a fully primed immune response. Virus excretion in saliva is an important mechanism for host-to-host transmission of both murine and human cytomegaloviruses [1]. In mice, salivary glands also represent the first site to produce virus after reactivation [8]. In fact, MCMV was originally isolated from a salivary gland of a persistently infected mouse and was named salivary gland virus of the mouse [24]. Altogether, our results indicate that at least one of these two MCMV miRNAs supports persistent MCMV infection in salivary glands. We speculate that this particular function of viral miRNA has evolved to allow virus spread to new hosts *via* saliva. Interestingly, although the knock-out of both miRNAs resulted only in a very mild attenuation in BALB/c mice, attenuation was substantially increased by reducing the dose of infection. Dependence on viral load in BALB/c mice perhaps implicates cross-talk between infected cells and thus implies the involvement of cytokines and/or chemokines. The fact that only combined depletion of NK cells and CD4^+^ T cells abolished the attenuation may also argue for cytokine dependent attenuation. Attenuation was preserved even in IFNγR^−/−^ mice, indicating that the mechanism is IFNγ independent. Upon severe immunosuppression, infection of connective tissue fibroblasts is also observed in salivary glands [21]. Therefore, we cannot exclude that insufficient immunological control following combined depletion of NK and CD4^+^ T cells simply masked the phenotype of viral miRNAs in persistently infected cells due to the contribution of lytic infection in surrounding cell populations like stromal fibroblasts.

### Prediction of cellular targets of miR-M23-2

In order to get a hint about the possible mechanism behind the observed phenotype of our mutant virus, we predicted the potential cellular targets of miR-M23-2 and miR-m21-1 using the RepTar algorithm [25]. From these predictions, we extracted all genes relevant to the immune response, and found 166 and 200 putative targets for miR-m21-1 and miR-M23-2 respectively (Table S1). Among these predictions, 77 genes were predicted to be targeted by both miRNAs. The chemokine CXCL16 was among the top predicted targets with multiple putative binding sites for both miR-m21-1 and miR-M23-2 (Fig. S4). The predicted binding of miR-M23-2 was more favorable than that of miR-m21-1 in terms of free energy of pairing. CXCL16 is a recently discovered chemokine that is expressed in both soluble and transmembrane forms, ligates to CXCR6 chemokine receptor and guides migration of activated Th1 and Tc1 cells [26], as well as NK cells [27]. It is mainly expressed by dendritic cells and macrophages, but also by fibroblasts and endothelial and epithelial cells [28], [29]. We cloned the full length 3′UTR of CXCL16 in a luciferase reporter vector, and tested its regulation by both miR-M23-2, and miR-m21-1. While no measurable regulation was observed for miR-m21-1, miR-M23-2 readily regulated the CXCL16 reporter (Fig. S5A). In addition, using 2′-O-methylated antisense oligonucleotide, we confirmed that the CXCL16 reporter regulation by miR-M23-2 could be inhibited in a sequence specific manner (Fig. S5B). In order to test whether miR-M23-2 could also regulate the CXCL16 reporter in the context of virus infection, we transfected the CXCL16 luciferase reporter in cells infected either with the miR-M23-2-mut virus or its revertant. The repression of a luciferase reporter containing either a perfect match sensor, a mismatched sensor for miR-M23-2 or the 3′UTR of CXCL16, was only detectable in cells infected with the revertant, but not the mutant virus (Fig. S5C).

Both cellular and viral miRNAs are known to target a large number of different genes. As such, hcmv-miR-UL112-1 targets a number of viral transcripts including the major viral transactivator IE1 as well as the host's NK cell activating ligand MICB [25], [30], [31]. Knock-out of both miR-M23-2 and miR-m21-1 probably resulted in the loss of regulation of several genes. Therefore, although preliminary observations indicate that CXCL16 is a target of miR-M23-2 but not of miR-m21-1, it is very unlikely that its regulation is the sole reason for the attenuation of miR-M23-2/m21-1 mutant viruses. This concept is also supported by our findings that attenuation of the miR-M23-2/m21-1 mutant viruses revealed dependence on host genetics and viral load, indicating a multifactorial rather than a single mode of regulation. Indeed, our target predictions contained numerous other genes involved in innate immune response (Table S1). Although the exact mechanism by which miR-M23-2 and miR-m21-1 contribute to an efficient accumulation in salivary glands remains to be identified, we hypothesize that these viral miRNAs contribute to the generation of a microenvironment in this organ that is favorable for the virus to persist. Additional mechanisms such as the regulation of viral gene expression might also contribute to the observed phenotype. In this context, it is not surprising that lifting only one type of control, e.g. depletion of CD4^+^ T cells, was not sufficient to completely revert viral miRNA function.

Viral miRNAs have been implicated in the control of viral latency and reactivation although formal proof *in vivo* is still lacking [3]. Our data not only provide the first *in vivo* phenotype of viral miRNAs but also demonstrate that viral miRNAs are important in chronic infection in a site well known for its significance in host-to-host transmission.

## Materials and Methods

### Ethics statement

All of the protocols used for breeding of mice and different kinds of treatments were approved by the Ethical Committee of the Faculty of Medicine University of Rijeka and were performed in accordance with Croatian Law for the Protection of Laboratory Animals, which has been harmonized with the existing EU legislation (EC Directive 86/609/EEC).

### Construction of mutant viruses

As pre-miR-M23-2 and pre-miR-m21-1 are located on opposing strands of the MCMV genome and are 98% complementary (the predicted 5′-end of pre-miR-m21-1 extends the 3′-end of pre-miR-M23-2 by one nucleotide), deletion of one miRNA always results in concordant knock-out of the other. To exclude unexpected effects on the genomic locus by the miRNA knock-out we constructed two independent mutants and revertants. The generation of the first mutant (MCMV-ΔmiR-M23-2) has been described [11]. The revertant virus was created by replacing the 276 bp insert with an expression cassette for galactokinase (GalK) and kanamycin resistance (Kn) in DH10B E.coli. The linear DNA fragment was generated by PCR on pGPS-GalK/Kn using primers H5-miR-M23-2-galK/Kn and H3-miR-M23-2-galk/Kn. Sequences of these and all other primers are provided in Table S2. After transferring the recombinant BAC to SW102 bacteria by electroporation, the wt pre-miR-M23-2 sequence was restored by traceless mutagenesis using a linear DNA fragment generated by PCR on pSM3fr using the PCR primers H5-miR-M23-rev and H3-miR-M23-rev [14]. The second mutant (MCMV-miR-M23-2-mut) was constructed solely in SW102 bacteria. First, the whole m21/m22/M23 locus of pSM3fr was replaced using homologous recombination by a GalK/Kn cassette amplified from pGPS-GalK/kn using primers H5-GalK/Kn-m22 and H3-GalK/Kn-m22. Next, the whole m21/m22/M23 miRNA cluster containing 4 miRNAs and part of the m22 gene was subcloned into pGPS1.1 (resulting in pGPS-m22) by PCR cloning using primers m22-for and m22-rev. To generate the mutant pre-miR-M23-2 template, 17 point mutations were inserted into the pre-miR-M23-2 within pGPS-m22 (resulting in pGPS-m22-miR-M23-2-mut) by oligonucleotide cloning with the two oligonucleotides pre-miR-M23-2-mut-s and pre-miR-M23-2-mut-as and an ApaL1 and PvuI digest. The m21/m22/M23 locus with the mutated pre-miR-M23-2 was excised from pGPS-m22-miR-M23-2-mut using EcoRV and inserted into pSM3fr-m22-galK/Kn by traceless mutagenesis resulting in the MCMV-M23-2-mut BAC. To generate the revertant, the GalK/Kn cassette was reinserted again as described above followed by reinsertion of the wt m21/m22/M23 miRNA cluster using the EcoRV fragment of pGPS-m22.

All viruses were reconstituted by transfecting the recombinant BACs into murine embryonic fibroblasts as described [23]. Virus titers were determined on MEFs by standard plaque assay [23].

### Northern blot analysis

RNA was extracted using TRIzol and northern blotting was performed on 10 µg of total RNA as described before [11], [32]. Probes were 5′ ^32^P-radiolabelled oligodeoxynucleotides perfectly complementary to the miRNA sequence or to part of the U6 snRNA sequence. Blots were analyzed and quantified by phosphorimaging using a FLA5100 scanner from Fuji.

### Quantitative PCR analysis of viral transcripts

Real time PCR analysis of m21 and M23 transcripts was performed as described before [12] using primers m21for and m21rev and M23for and M23rev respectively as indicated in Table S2.

### Target prediction by RepTar algorithm

RepTar is a miRNA target prediction algorithm that is independent of evolutionary conservation considerations and is not limited to seed pairing sites. It is based on the finding that in some targets the miRNA binding site repeats in the 3′UTR. It identifies high scoring repetitive elements in each 3′UTR, matches them to the miRNA sequences and evaluates them as candidate miRNA binding sites. Based on the information gained from these repetitive sites, RepTar then searches for non-repetitive sites as well. The final set of Reptar predictions includes targets with conserved or non-conserved single or multiple binding sites of several types including: seed binding sites, seed wobble sites (seed sites that include G:U pairing in the seed), 3′ compensatory binding sites and full-match binding sites. The special properties of the algorithm make it advantageous for predicting targets of the less conserved viral miRNAs.

### Dual luciferase experiments

The perfect match sensor for miR-M23-2 was obtained by annealing the oligonucleotides PM-M23-2-for and PM-M23-2-rev. The miR-M23-2 mismatch sensor was obtained by annealing the oligonucleotides MM-M23-2-for and MM-M23-2-rev. The 3′UTR of CXCL16 (position 16–1100) was PCR amplified from NIH-3T3 genomic DNA using primers CXCL16-for and CXCL16-rev. To each product, AttB1 and AttB2 sites were incorporated by PCR using primers AttB1-for and AttB2-rev (see Table S2). The resulting PCR products were then recombined sequentially in pDONR/Zeo and in psiCHECK-2 plasmids using Gateway technology (Invitrogen). NIH-3T3 cells were infected with wt MCMV, MCMV-miR-M23-2-mut as well as with MCMV-miR-M23-2-mut-rev at an MOI = 10 using centrifugal enhancement. 24 h post infection cells were transfected with psiCHECK-PM-M23-2 using the TransIT-3T3 Transfection kit (Mirus) following the manufacturer's instructions. At 48 and 72 h post infection luciferase activities were measured employing the Dual-Luciferase Reporter Assay (Promega).

For CXCL16 dual luciferase assays, constructs were co-transfected into NIH-3T3 cells with the indicated concentrations of miRNA mimic or Allstars negative control siRNA (Qiagen), using lipofectamine 2000 (Invitrogen) as per manufacturer's instructions. Luciferase activities were measured 48 h post transfection (Glomax, Promega). The inhibition of miRNA activity was performed by co-transfection of 100 nM 2′-O-methylated oligonucleotides specific for miR-M23-2 or for *C. elegans* miR-67 as a control. For CXCL16 luciferase assays in MCMV-infected cells, NIH-3T3 cells were infected with MCMV-miR-M23-2 mut or MCMV-miR-M23-2-mut-rev at an MOI = 10, using centrifugal enhancement. 24h post infection cells were transfected with psiCHECK-PM-M23-2, psiCHECK-MM-M23-2 or psiCHECK-CXCL16 using lipofectamine 2000 (Invitrogen) as per manufacturer's instructions. Luciferase activities were measured 48 h post transfection (Glomax, Promega).

### Mice

BALB/c (H-2^d^), C57BL/6 (H-2^b^), CBA/J (H-2^k^), DBA/2 (H-2^d^), 129/SvJ (H-2^b^) and 129/SvJ IFNγR^-/-^ (H-2^b^) mice were housed and bred under specific-pathogen-free conditions at the Central Animal Facility, Faculty of Medicine, University of Rijeka.

### Infection conditions and detection of infectious MCMV in tissues, depletion of lymphocyte subsets, and statistical evaluation

Mice were injected either intraperitoneally or intravenously with indicated doses (1×10^4^ PFU to 5×10^5^ PFU) of tissue culture-grown wt MCMV or recombinant viruses in 0.5 ml of diluent. Organs were collected either 14 or 25 days after infection and virus titers were determined by a standard plaque-forming assay [33]. In vivo depletion of CD4^+^ and CD8^+^ T lymphocyte subsets and NK cells was performed by intraperitoneal injection of the mAbs to CD4 (YTS191.1), to CD8 (YTS 169.4) molecules [34] and to NK1.1 (PK136) [35]. Statistical significance of the difference between experimental groups was determined by the Mann-Whitney exact rank test.

## Supporting Information

Figure S1 Quantitative analysis of miR-M23-2 flanking transcripts and miRNAs. **A and B.** qRT-PCR analysis of m21 and M23 transcripts accumulation in wt MCMV, ΔmiR-M23-2, miR-M23-2-mut and their revertants infected NIH-3T3 cells at 48 hpi. Data was normalized to IE1 expression levels. **C.** Northern blot analysis of miR-M23-1-3p and miR-m22-1 accumulation in cells infected with wt MCMV, ΔmiR-M23-2, miR-M23-2-mut and their revertant viruses at 48 hpi. M, mock infected cells; EtBr, Ethidium Bromide.
(0.24 MB TIF)

Figure S2 Virus titers of wt MCMV, ΔmiR-M23-2 and miR-M23-2-mut in various organs at 3 days post infection. C57BL/6 and BALB/c mice were injected i.v. with 5×10^5^ or 3×10^5^ PFU, respectively with wt MCMV, ΔmiR-M23-2 and miR-M23-2-mut, as well as with their respective revertants. Virus titers in organs were determined 3 days post infection. Titers in organs of individual mice (circles) and median values (horizontal bars) are shown. DL  =  detection limit.
(0.12 MB TIF)

Figure S3 Virus titers of wt MCMV, miR-M23-2-mut and miR-M23-2-mut-rev in BALB/c salivary glands and lungs at 14 days post intraperitoneal infection. Titers in organs of individual mice (circles) and median values (horizontal bars) are shown. DL  =  detection limit; * p<0.05; ** p<0.01.
(0.11 MB TIF)

Figure S4 Schematic representation of miR-M23-2 and miR-m21-1 binding sites within the CXCL16 3′UTR. Shown are the sites predicted by RepTar version 1.1, based on the statistical profiles of repeating elements in the 3′UTR. The miRNA is indicated in red on top of the alignment. For miR-M23-2 there is an additional full seed match at position 221, which was not detected by the version of the algorithm used at the time of the analysis. Loc: position in 3′UTR (location is zero based). ΔG: free energy of pairing in Kcal/mol.
(0.31 MB TIF)

Figure S5 Identification of a putative cellular target of miR-M23-2. **A.** CXCL16 is regulated by miR-M23-2, but not by miR-m21-1. NIH-3T3 fibroblasts were co-transfected with a bulged luciferase sensor for miR-M23-2 or a luciferase reporter construct containing the entire CXCL16 3′UTR, and with the indicated miRNA oligonucleotides mimics or negative control siRNA. Dual luciferase assays were performed 48 h post-transfection (n = 5). FLUC to RLUC ratios were first normalized to the values obtained for the empty reporter vector and then to the values obtained with the negative control siRNA, which were set to 1. **B.** Regulation by miR-M23-2 oligonucleotide mimic of both mismatched sensor for miR-M23-2 and CXCL16 3′UTR luciferase reporter can be reverted by co-transfection of a 2′-O-methylated (2′O Me) antisense oligonucleotide directed against miR-M23-2, but not by a control (Ctrl) 2′-O-methylated antisense oligonucleotide directed against the *C. elegans* miRNA miR-67. Dual luciferase assays were performed 48 h post-transfection (n = 6). FLUC to RLUC ratios were first normalized to the values obtained for the empty reporter vector and then to the values obtained with the control 2′-O-methylated oligonucleotide, which were set to 1. **C.** Regulation of perfect match (PM), mismatch (MM) sensors for miR-M23-2 or CXCL16 3′UTR luciferase reporters in cells infected with MCMV-miR-M23-2-mut or its corresponding revertant. Dual luciferase assays were performed 48 h post-transfection (n = 3). Shown here is a representative example of four independent experiments.
(0.46 MB TIF)

Table S1 Immuno-related predicted targets of miR-m21-1 and miR-M23-2. Gene annotations were extracted from http://cgap.nci.nih.gov/Genes/GOBrowser. All genes with the GO Biological Process category: “immune system process” were considered immuno-related. For each target gene the number of predicted binding sites and the binding site with minimal free energy upon miRNA:3′UTR pairing are reported. The minimal free energy value and the pattern of base-pairing for this site are shown (computed by the program RNAcofold of Vienna package). miRNA and mRNA are displayed as the upper and lower string, respectively. A solid line represents canonical Watson-Crick base pairing, and a colon represents G:U base pairing.
(1.04 MB PDF)

Table S2 List of PCR primers and oligonucleotides.
(0.23 MB PDF)
