# Supplementary figures and images for: Cytomegalovirus microRNAs Facilitate Persistent Virus Infection in Salivary Glands
Source: PLoS Pathog. 2010 Oct 14;6(10):e1001150. doi: 10.1371/journal.ppat.1001150 (PMC2954898; doi:10.1371/journal.ppat.1001150)

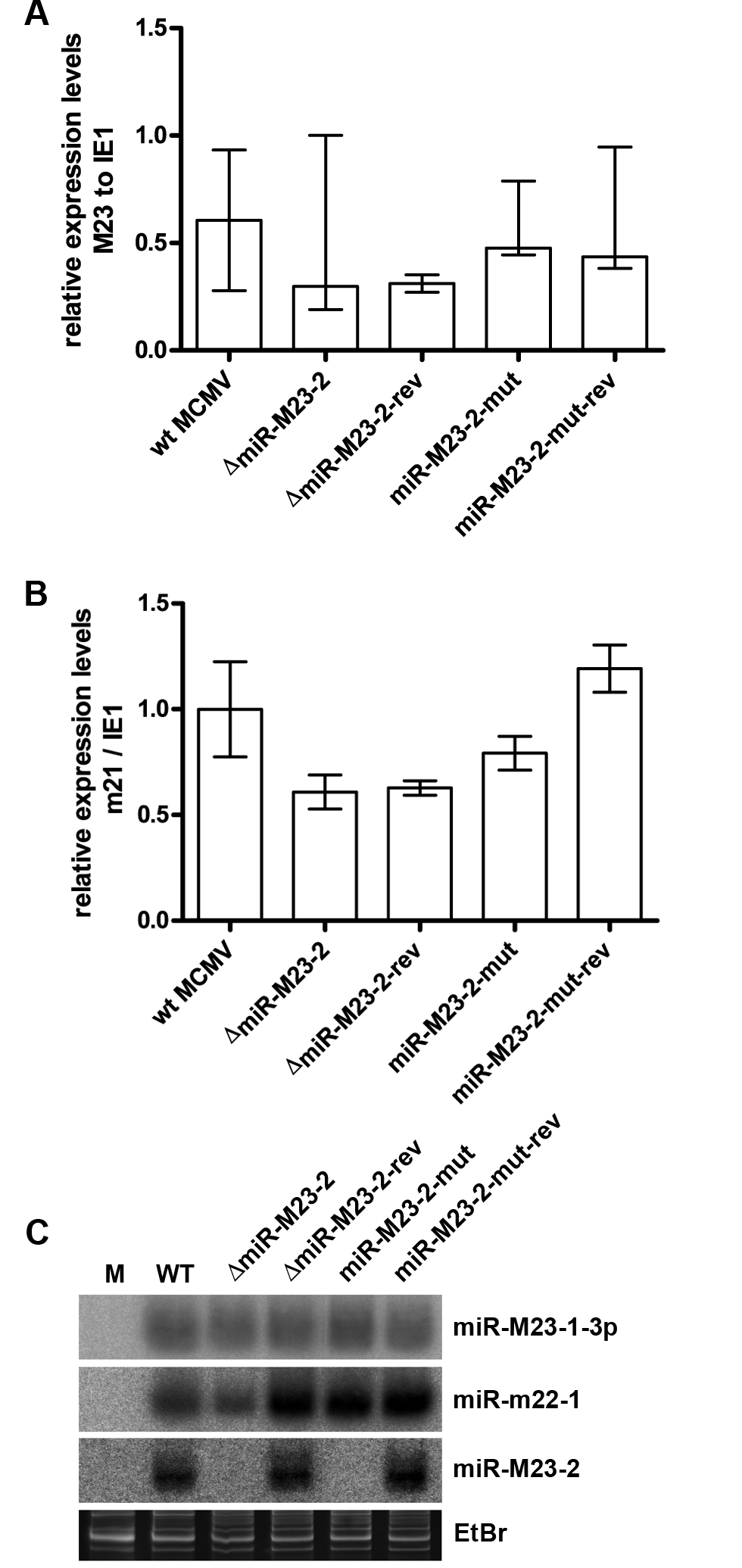

Supplement: Figure S1 — Quantitative analysis of miR-M23-2 flanking transcripts and miRNAs. A and B. qRT-PCR analysis of m21 and M23 transcripts accumulation in wt MCMV, ΔmiR-M23-2, miR-M23-2-mut and their revertants infected NIH-3T3 cells at 48 hpi. Data was normalized to IE1 expression levels. C. Northern blot analysis of miR-M23-1-3p and miR-m22-1 accumulation in cells infected with wt MCMV, ΔmiR-M23-2, miR-M23-2-mut and their revertant viruses at 48 hpi. M, mock infected cells; EtBr, Ethidium Bromide. (0.24 MB TIF) [file ppat.1001150.s001.tif]

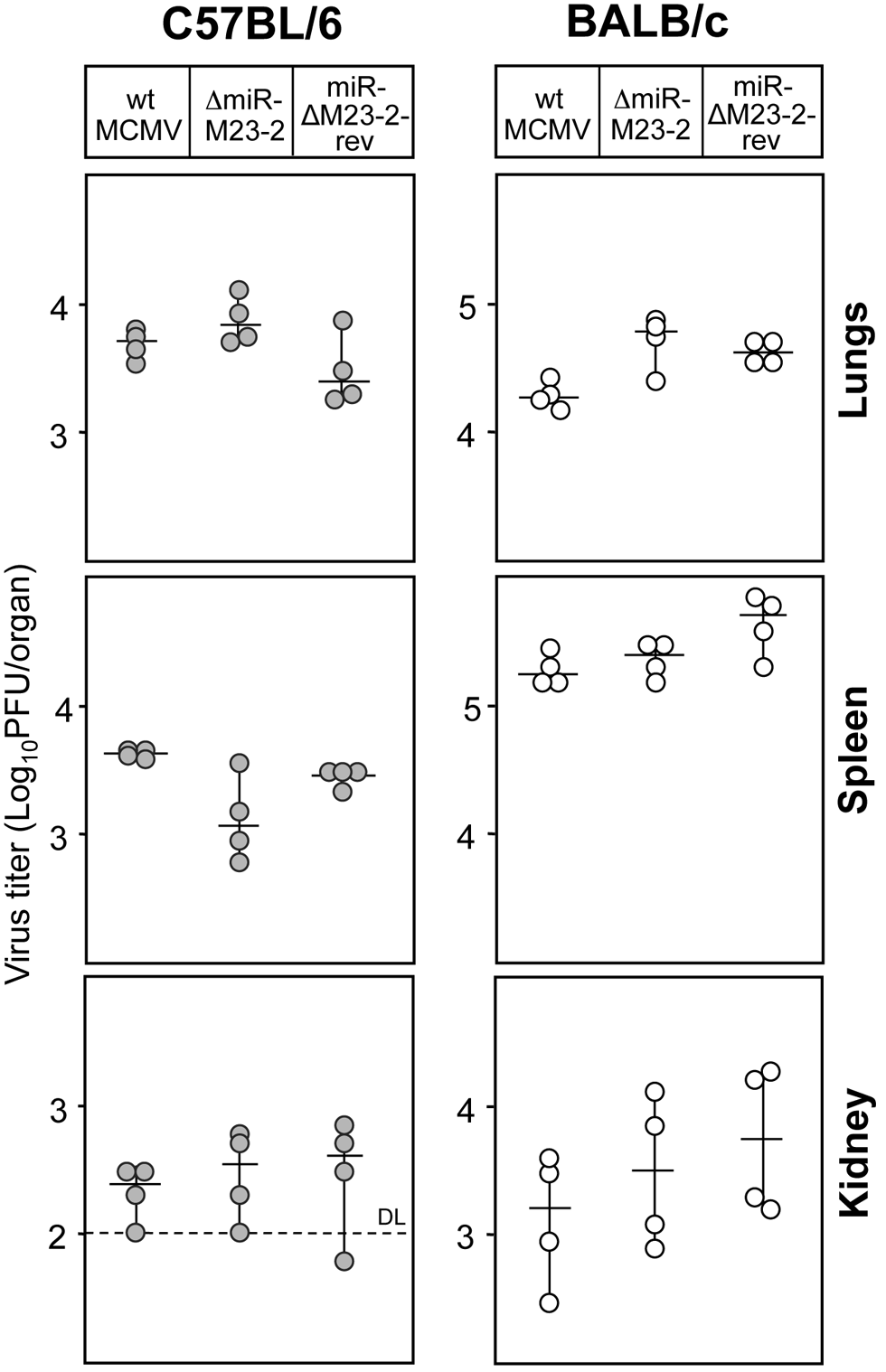

Supplement: Figure S2 — Virus titers of wt MCMV, ΔmiR-M23-2 and miR-M23-2-mut in various organs at 3 days post infection. C57BL/6 and BALB/c mice were injected i.v. with 5×10^5^ or 3×10^5^ PFU, respectively with wt MCMV, ΔmiR-M23-2 and miR-M23-2-mut, as well as with their respective revertants. Virus titers in organs were determined 3 days post infection. Titers in organs of individual mice (circles) and median values (horizontal bars) are shown. DL = detection limit. (0.12 MB TIF) [file ppat.1001150.s002.tif]

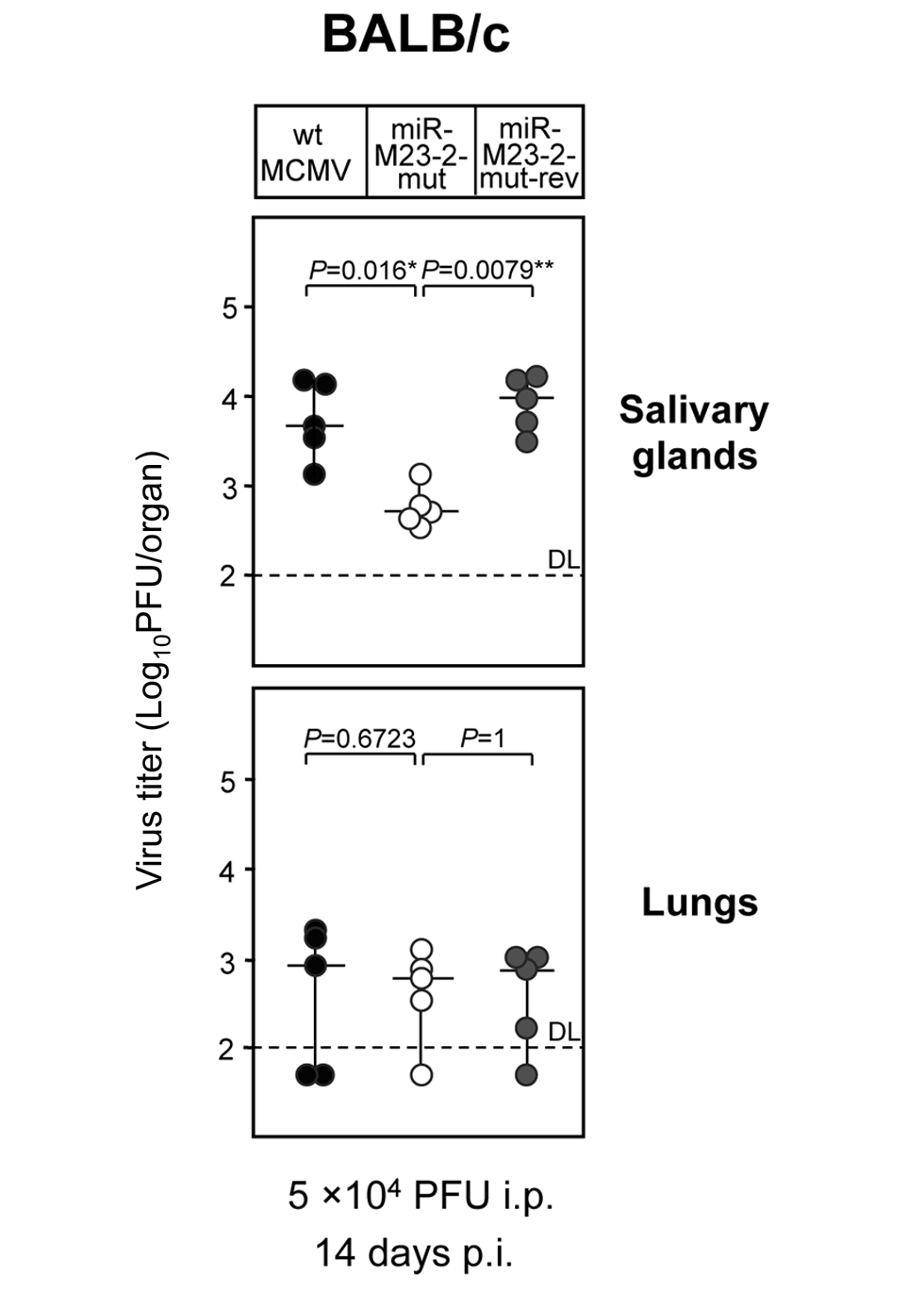

Supplement: Figure S3 — Virus titers of wt MCMV, miR-M23-2-mut and miR-M23-2-mut-rev in BALB/c salivary glands and lungs at 14 days post intraperitoneal infection. Titers in organs of individual mice (circles) and median values (horizontal bars) are shown. DL = detection limit; * p<0.05; ** p<0.01. (0.11 MB TIF) [file ppat.1001150.s003.tif]

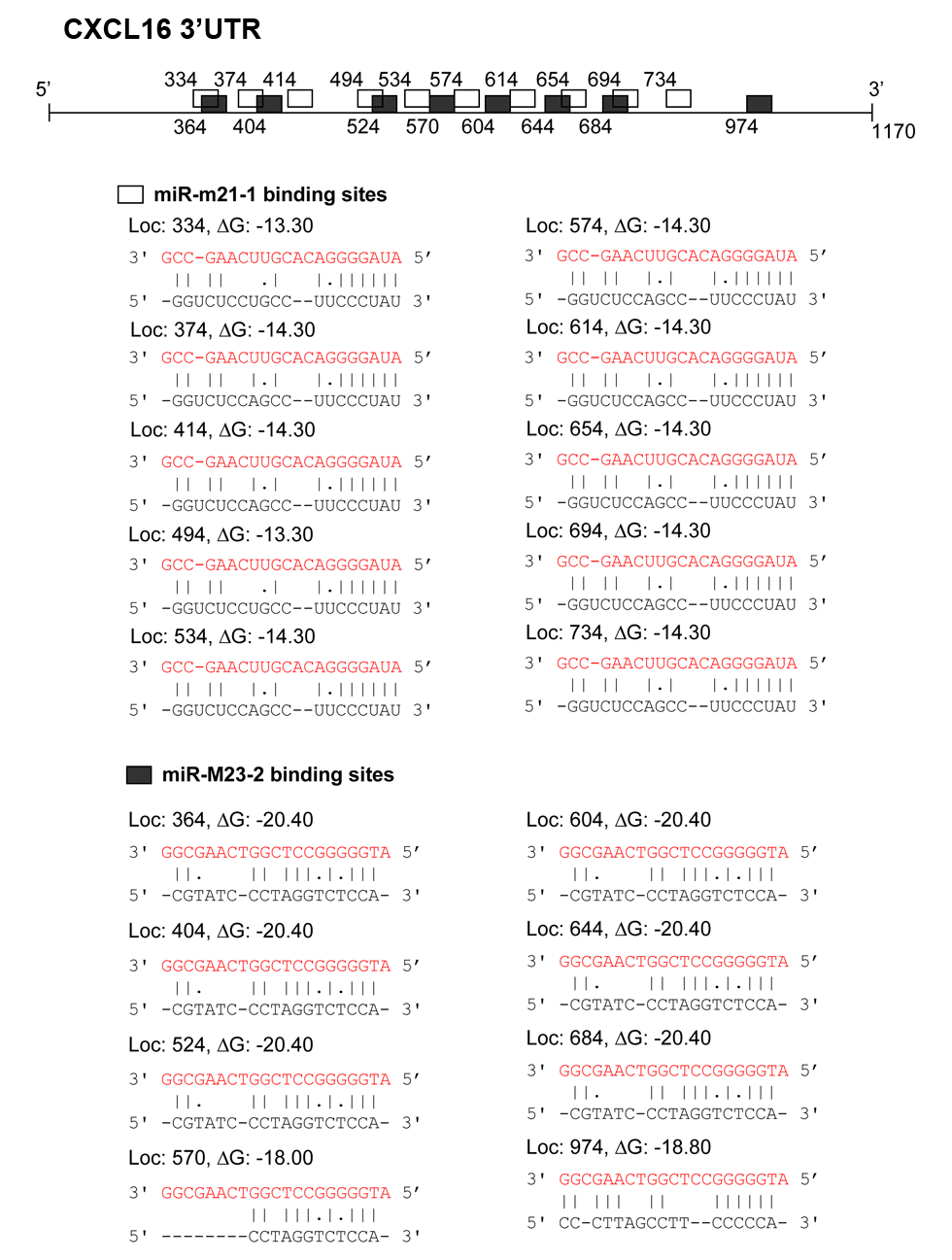

Supplement: Figure S4 — Schematic representation of miR-M23-2 and miR-m21-1 binding sites within the CXCL16 3′UTR. Shown are the sites predicted by RepTar version 1.1, based on the statistical profiles of repeating elements in the 3′UTR. The miRNA is indicated in red on top of the alignment. For miR-M23-2 there is an additional full seed match at position 221, which was not detected by the version of the algorithm used at the time of the analysis. Loc: position in 3′UTR (location is zero based). ΔG: free energy of pairing in Kcal/mol. (0.31 MB TIF) [file ppat.1001150.s004.tif]

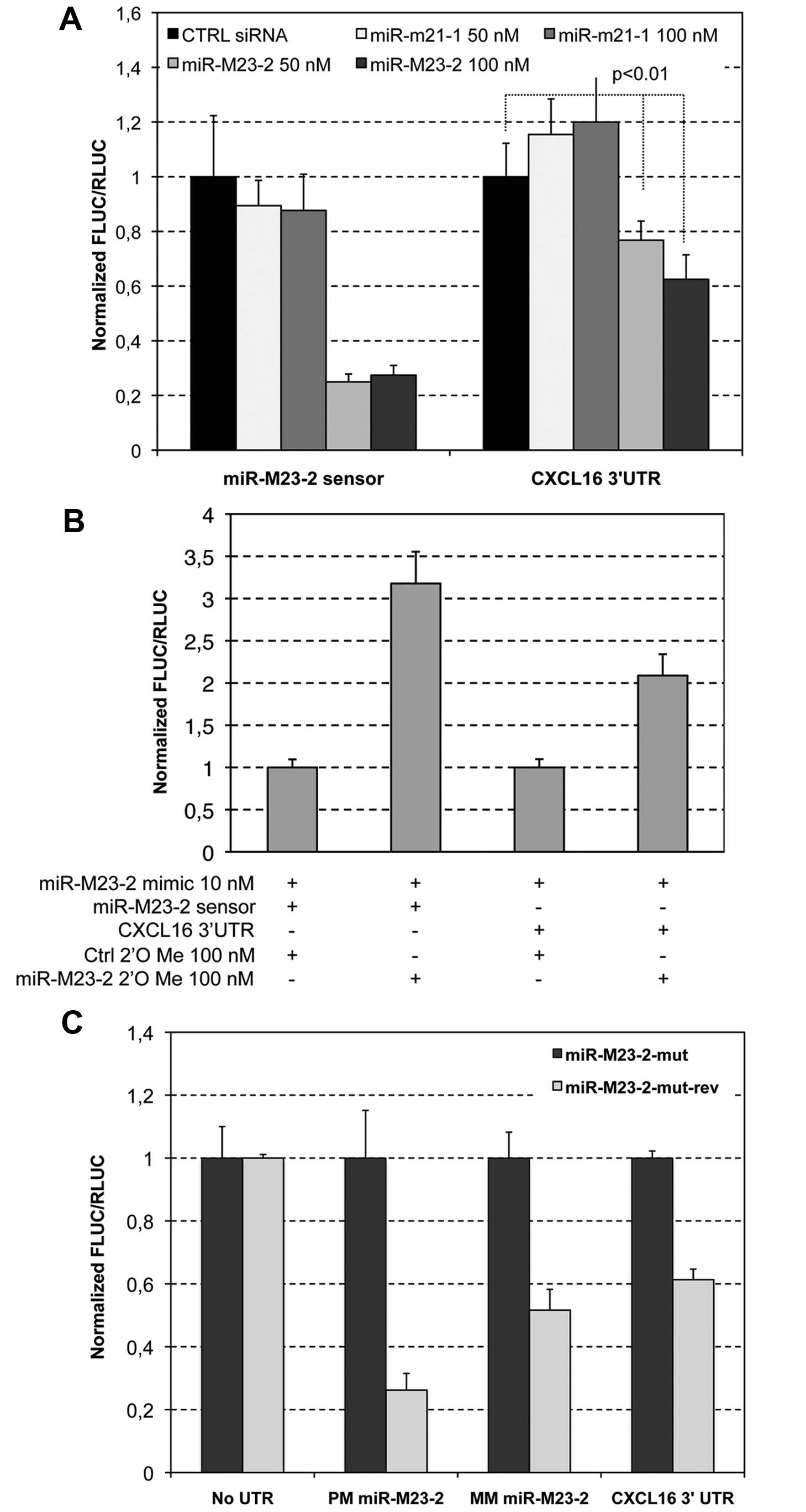

Supplement: Figure S5 — Identification of a putative cellular target of miR-M23-2. A. CXCL16 is regulated by miR-M23-2, but not by miR-m21-1. NIH-3T3 fibroblasts were co-transfected with a bulged luciferase sensor for miR-M23-2 or a luciferase reporter construct containing the entire CXCL16 3′UTR, and with the indicated miRNA oligonucleotides mimics or negative control siRNA. Dual luciferase assays were performed 48 h post-transfection (n = 5). FLUC to RLUC ratios were first normalized to the values obtained for the empty reporter vector and then to the values obtained with the negative control siRNA, which were set to 1. B. Regulation by miR-M23-2 oligonucleotide mimic of both mismatched sensor for miR-M23-2 and CXCL16 3′UTR luciferase reporter can be reverted by co-transfection of a 2′-O-methylated (2′O Me) antisense oligonucleotide directed against miR-M23-2, but not by a control (Ctrl) 2′-O-methylated antisense oligonucleotide directed against the C. elegans miRNA miR-67. Dual luciferase assays were performed 48 h post-transfection (n = 6). FLUC to RLUC ratios were first normalized to the values obtained for the empty reporter vector and then to the values obtained with the control 2′-O-methylated oligonucleotide, which were set to 1. C. Regulation of perfect match (PM), mismatch (MM) sensors for miR-M23-2 or CXCL16 3′UTR luciferase reporters in cells infected with MCMV-miR-M23-2-mut or its corresponding revertant. Dual luciferase assays were performed 48 h post-transfection (n = 3). Shown here is a representative example of four independent experiments. (0.46 MB TIF) [file ppat.1001150.s005.tif]
