# Supplementary material for: Cytomegalovirus microRNAs Facilitate Persistent Virus Infection in Salivary Glands
Source: PLoS Pathog. 2010 Oct 14;6(10):e1001150. doi: 10.1371/journal.ppat.1001150 (PMC2954898; doi:10.1371/journal.ppat.1001150)
Supplement: Table S1 — Immuno-related predicted targets of miR-m21-1 and miR-M23-2. Gene annotations were extracted from http://cgap.nci.nih.gov/Genes/GOBrowser. All genes with the GO Biological Process category: “immune system process” were considered immuno-related. For each target gene the number of predicted binding sites and the binding site with minimal free energy upon miRNA:3′UTR pairing are reported. The minimal free energy value and the pattern of base-pairing for this site are shown (computed by the program RNAcofold of Vienna package). miRNA and mRNA are displayed as the upper and lower string, respectively. A solid line represents canonical Watson-Crick base pairing, and a colon represents G:U base pairing. (1.04 MB PDF) [file ppat.1001150.s006.pdf]

Table S1. Page 1

miR-m21-1 predicted targets ranked by minimal free energy (MFE) of binding

<table>
<tr><th>Predicted Target</th><th></th><th></th><th colspan="2">Binding site with minimal free energy (MFE)</th></tr>
<tr><th>Gene symbol:::RefSeq</th><th>miRNA</th><th># binding sites</th><th>MFE (Kcal/mol)</th><th>Binding site</th></tr>
<tr><td>Twsg1:::NM_023053</td><td>mcmv-miR-m21-1</td><td>1</td><td>-29.7</td><td>3' GCCGAACTTGCA---CAGGGGATA 5'
    ||:|||:|:|| |||||||:|
5' -GGTTTGGATGTTATGTCCCCTGT 3'</td></tr>
<tr><td>Blr1:::NM_007551</td><td>mcmv-miR-m21-1</td><td>1</td><td>-28</td><td>3' GC-CGAACTTG--CACAGGGGATA 5'
    | |||||::| ||||||||
5' -GAGCTTGGGCCAGTGTCCCC--- 3'</td></tr>
<tr><td>Olr1:::NM_138648</td><td>mcmv-miR-m21-1</td><td>3</td><td>-25.1</td><td>3' GCCGAACTT--GCACAGGGGATA 5'
    | ||||| ||||||||||:
5' -GAATTGAATTCGTGTCCCCTG- 3'</td></tr>
<tr><td>Kdr:::NM_010612</td><td>mcmv-miR-m21-1</td><td>2</td><td>-24.7</td><td>3' GCC-GAACTTGCACAGGGGATA 5'
   ||| || |||||||:|||:
5' CGGACTCTTACGTGTCTCCTG- 3'</td></tr>
<tr><td>Osm:::NM_001013365</td><td>mcmv-miR-m21-1</td><td>2</td><td>-24.7</td><td>3' GCCGAACTTG----CACAGGGGATA 5'
    || |||| ||||||||||
5' -GGGGTGAAGTTGAGTGTCCCCTA- 3'</td></tr>
<tr><td>Cd300lb:::NM_199221</td><td>mcmv-miR-m21-1</td><td>1</td><td>-24.3</td><td>3' GCCGAA---CTTGCACAGGGGATA 5'
    ||| |||:| |||||||:
5' -GGCCACGAGAATG-GTCCCCTG- 3'</td></tr>
<tr><td>Cd4:::NM_013488</td><td>mcmv-miR-m21-1</td><td>1</td><td>-23.5</td><td>3' GCCGAACTTGC-ACAGGGGATA 5'
      |||||| ||||||||||
5' ---CTTGAAAGTTGTCCCCTAT 3'</td></tr>
<tr><td>Otub1:::NM_134150</td><td>mcmv-miR-m21-1</td><td>1</td><td>-22.8</td><td>3' GC-CGAACTTGCACAGGGGATA 5'
    | ||||| ||||||||:|
5' -GAGCTTGCC--TGTCCCCTGT 3'</td></tr>
<tr><td>Cxcl14:::NM_019568</td><td>mcmv-miR-m21-1</td><td>2</td><td>-22.7</td><td>3' GCCGA--ACTTGCACAGGGGATA 5'
    ||:| |||| |||||||:
5' -GGTTTCTGAAAG-GTCCCCTG- 3'</td></tr>
<tr><td>Syk:::NM_011518</td><td>mcmv-miR-m21-1</td><td>2</td><td>-22.52</td><td>3' GCCGAACTTGCACAGGGGATA 5'
    || |||||||||:
5' -GGATTTCA-GTGTCCCCTG- 3'</td></tr>
<tr><td>H2-Eb1:::NM_010382</td><td>mcmv-miR-m21-1</td><td>2</td><td>-22.5</td><td>3' GCCGAA-CTTGCACAGGGGATA 5'
    ||| |:|| |||||||
5' -GGCGGGGGACCCATCCCCTA- 3'</td></tr>
<tr><td>Smad3:::NM_016769</td><td>mcmv-miR-m21-1</td><td>2</td><td>-22.3</td><td>3' GCCGA---ACTTGCACAGGGGATA 5'
    |||| |||| |||||:||:|
5' -GGCTCTTTGAAA-TGTCCTCTGT 3'</td></tr>
<tr><td>G6pdx:::NM_008062</td><td>mcmv-miR-m21-1</td><td>1</td><td>-22.2</td><td>3' GCCGAACTTGC-ACAGGGGATA 5'
    |||| | ||||||||:
5' -GGCTCTA--GATGTCCCCTG- 3'</td></tr>
<tr><td>Slamf1:::NM_013730</td><td>mcmv-miR-m21-1</td><td>2</td><td>-22.2</td><td>3' GCCGAACTTGCA---CAGGGGATA 5'
    ||| ||:|| |||||||:
5' -GGCATAAATGTCAAGTCCCCTG- 3'</td></tr>
<tr><td>Oas1b:::NM_001083925</td><td>mcmv-miR-m21-1</td><td>1</td><td>-22.1</td><td>3' GCCGAACTTGCACAGGGGATA 5'
    ||||| | ||||||||:
5' -GGCTT---C-TGTCCCCTG- 3'</td></tr>
<tr><td>Stat5b:::NM_011489</td><td>mcmv-miR-m21-1</td><td>2</td><td>-22.1</td><td>3' GCCGAACTTGCACAGGGGATA 5'
            ||||||||||:|
5' ---------CGTGTCCCCTGT 3'</td></tr>
<tr><td>Bcap31:::NM_012060</td><td>mcmv-miR-m21-1</td><td>1</td><td>-21.9</td><td>3' GCCGAACTTGCAC-AGGGGATA 5'
    |||||| ||| |:||||:
5' -GGCTTG---GTGTTTCCCTG- 3'</td></tr>
<tr><td>Hif1a:::NM_010431</td><td>mcmv-miR-m21-1</td><td>1</td><td>-21.9</td><td>3' GCCGAA--CTTGCACAGGGGATA 5'
    ||: ||: ||||||:|||
5' -GGTGAGGGAGTTTGTCCCTTAT 3'</td></tr>
<tr><td>Il1rl1:::NM_001025602</td><td>mcmv-miR-m21-1</td><td>1</td><td>-21.9</td><td>3' GCCGAACTTGCAC-AGGGGATA 5'
    ||| :||| ||||||||
5' -GGCCT---TGTGTTCCCCTAT 3'</td></tr>
<tr><td>Traf6:::NM_009424</td><td>mcmv-miR-m21-1</td><td>3</td><td>-21.9</td><td>3' GCCGAACTTGCACAGGGGATA 5'
    || || ||||||||:|
5' -GGAACCTACATGTCCCCTGT 3'</td></tr>
<tr><td>Blnk:::NM_008528</td><td>mcmv-miR-m21-1</td><td>1</td><td>-21.6</td><td>3' GCCGAAC-TTGCACAGGGGATA 5'
   |||:| :|| |||||:||:|
5' CGGTTCCAGACATGTCCTCTGT 3'</td></tr>
<tr><td>Podxl:::NM_013723</td><td>mcmv-miR-m21-1</td><td>3</td><td>-21.6</td><td>3' GCCGAACT-TGCACAGGGGATA 5'
    ||| || | ||||||:||:
5' -GGCA-GACAGGTGTCCTCTG- 3'</td></tr>
<tr><td>Cd86:::NM_019388</td><td>mcmv-miR-m21-1</td><td>1</td><td>-21.1</td><td>3' GCCGAACTTGCACAGGGGATA 5'
             |||||||||||
5' ----------GTGTCCCCTAT 3'</td></tr>
<tr><td>Cd300lg:::NM_027987</td><td>mcmv-miR-m21-1</td><td>1</td><td>-21</td><td>3' GCCGAACTTGCACAGGGGATA 5'
    ||| :|||||||:|:
5' -GGCCC---TGTGTCCCTTG- 3'</td></tr>
<tr><td>Hbb-b1:::NM_008220</td><td>mcmv-miR-m21-1</td><td>1</td><td>-20.5</td><td>3' GCCGAACTTGCACAGGGGATA 5'
    ||:| | |||||||||
5' -GGTT---ATATGTCCCCTA- 3'</td></tr>
<tr><td>Hs1bp3:::NM_021429</td><td>mcmv-miR-m21-1</td><td>1</td><td>-20.5</td><td>3' GCCGAACTTGCACAGGGGATA 5'
             |||||||||:|
5' ----------GTGTCCCCTGT 3'</td></tr>
<tr><td>Colec12:::NM_130449</td><td>mcmv-miR-m21-1</td><td>1</td><td>-20.4</td><td>3' GCCGAACTTGCACAGGGGATA 5'
    |||||| ||||||:
5' -GGCTTGCT----TCCCCTG- 3'</td></tr>
<tr><td>Cplx2:::NM_009946</td><td>mcmv-miR-m21-1</td><td>3</td><td>-20.1</td><td>3' GCCGAACTTGCACAGGGGATA 5'
   |||| ||||||||:
5' CGGCCAA----TGTCCCCTG- 3'</td></tr>
<tr><td>Pik3r1:::NM_001024955</td><td>mcmv-miR-m21-1</td><td>1</td><td>-20.1</td><td>3' GCCGAACTTGCACAGGGGATA 5'
    |||| ||||:|||:|
5' -GGCTGTT---TGTCTCCTGT 3'</td></tr>
<tr><td>Clec5a:::NM_001038604</td><td>mcmv-miR-m21-1</td><td>3</td><td>-19.9</td><td>3' GCCGAACTTGC--ACAGGGGATA 5'
         ||:|| |||||:||||
5' ------GAGCGGTTGTCCTCTAT 3'</td></tr>
<tr><td>Lig4:::NM_176953</td><td>mcmv-miR-m21-1</td><td>1</td><td>-19.9</td><td>3' GCCGAACTTGCACAGGGGATA 5'
         |||| ||||:|||||
5' ------GAACTTGTCTCCTAT 3'</td></tr>
<tr><td>Rbpj:::NM_001080927</td><td>mcmv-miR-m21-1</td><td>1</td><td>-19.8</td><td>3' GCCGAACTTGCACAGGGGATA 5'
     |||| || ||||||:||
5' --GCTTTTAC-TGTCCCTTA- 3'</td></tr>
</table>

Supplementary Table 1. page 2

<table>
<tr><td>Cbfa2t3h:::NM_009824</td><td>mcmv-miR-m21-1</td><td>3</td><td>-19.56</td><td>3' GCCGAACTTGCACAGGGGATA 5'
    ||: |||||||:|
5' -GGTGCTCTTA-GTCCCCTGT 3'</td></tr>
<tr><td>Fancc:::NM_001042673</td><td>mcmv-miR-m21-1</td><td>2</td><td>-19.4</td><td>3' GCCGAAC--TTGCACAGGGGATA 5'
    ||| ||| |||||||:
5' -GGCCATAAAACC-GTCCCCTG- 3'</td></tr>
<tr><td>Cebpa:::NM_007678</td><td>mcmv-miR-m21-1</td><td>1</td><td>-19.34</td><td>3' GCCGAACTTGCACAGGGGATA 5'
    ||||| |||||||
5' -GGCTTCCCCCTCTCCCCTA- 3'</td></tr>
<tr><td>Il3:::NM_010556</td><td>mcmv-miR-m21-1</td><td>1</td><td>-19.2</td><td>3' GCCGA-ACTTGC--ACAGGGGATA 5'
    ||| || | ||||||:|:|
5' -GGCCATGTTCATTTGTCCCTTGT 3'</td></tr>
<tr><td>Fcgr2b:::NM_001077189</td><td>mcmv-miR-m21-1</td><td>1</td><td>-19.1</td><td>3' GCCGAACTTGCACAGGGGATA 5'
    || |||||:|||:
5' -GGATCTA--GTGTCTCCTG- 3'</td></tr>
<tr><td>Rorc:::NM_011281</td><td>mcmv-miR-m21-1</td><td>2</td><td>-19.1</td><td>3' GCCGAACTTGCACAGGGGATA 5'
     ||| |:| |||||:||:|
5' --GCTCCAGC-TGTCCTCTGT 3'</td></tr>
<tr><td>Sox6:::NM_001025559</td><td>mcmv-miR-m21-1</td><td>1</td><td>-19</td><td>3' GCCGAACTTGCACAGGGGATA 5'
   |||: |:|| ||||||:|
5' CGGT--GGAC---TCCCCTGT 3'</td></tr>
<tr><td>Tnfsf10:::NM_009425</td><td>mcmv-miR-m21-1</td><td>1</td><td>-18.6</td><td>3' GCCG--AACTTGCACAGGGGATA 5'
    ||: ||| | ||||:|||:|
5' -GGTCTTTGCCC-TGTCTCCTGT 3'</td></tr>
<tr><td>Ihh:::NM_010544</td><td>mcmv-miR-m21-1</td><td>1</td><td>-18.4</td><td>3' GCCGAACTTGCAC-AGGGGATA 5'
    |||| |:| ||||||:
5' -GGCTA-AGCTCCTTCCCCTG- 3'</td></tr>
<tr><td>H2-M3:::NM_013819</td><td>mcmv-miR-m21-1</td><td>1</td><td>-18.3</td><td>3' GCCGAACTTGCACAGGGGATA 5'
    ||: |||||:|||:|
5' -GGT------GTGTCTCCTGT 3'</td></tr>
<tr><td>Spn:::NM_009259</td><td>mcmv-miR-m21-1</td><td>1</td><td>-18.3</td><td>3' GCCGAACTTGCAC--AGGGGATA 5'
    ||| |||| || ||||:|:|
5' -GGC-TGAA-GTCTTTCCCTTGT 3'</td></tr>
<tr><td>Tgfbr3:::NM_011578</td><td>mcmv-miR-m21-1</td><td>3</td><td>-18.3</td><td>3' GCCGAACT-TGCACAGGGGATA 5'
     || |:| |||||||:|
5' --GCCCTACATG-GTCCCCTGT 3'</td></tr>
<tr><td>Cblb:::NM_001033238</td><td>mcmv-miR-m21-1</td><td>1</td><td>-18.1</td><td>3' GCCGAACTTGCACAGGGGATA 5'
    |||| :|| ||||||
5' -GGCTAAT-TGTAACCCCTA- 3'</td></tr>
<tr><td>Ccl24:::NM_019577</td><td>mcmv-miR-m21-1</td><td>1</td><td>-18.1</td><td>3' GCCGAACTTGCACAGGGGATA 5'
             |||||:|||:|
5' ----------GTGTCTCCTGT 3'</td></tr>
<tr><td>Cd27:::NM_001033126</td><td>mcmv-miR-m21-1</td><td>1</td><td>-18.1</td><td>3' GCCGAACTTGCAC-AGGGGATA 5'
    ||:   :| || ||||||:|
5' -GGTG---GCCTGCTCCCCTGT 3'</td></tr>
<tr><td>Cd3d:::NM_013487</td><td>mcmv-miR-m21-1</td><td>1</td><td>-17.9</td><td>3' GCCGAACTTGCACAGGGGATA 5'
   |||| :| ||||||:|
5' CGGC----GCCT-TCCCCTGT 3'</td></tr>
<tr><td>Fkbp1a:::NM_008019</td><td>mcmv-miR-m21-1</td><td>1</td><td>-17.9</td><td>3' GCCGAACTTGCACAGGGGATA 5'
     |||| ||||||||:
5' --GCTTT----TGTCCCCTG- 3'</td></tr>
<tr><td>Ccl8:::NM_021443</td><td>mcmv-miR-m21-1</td><td>1</td><td>-17.8</td><td>3' GCCGAACTTGCAC-AGGGGATA 5'
     ||| ||| || |||||||
5' --GCTGGAA-GTTCTCCCCTA- 3'</td></tr>
<tr><td>Cd34:::NM_133654</td><td>mcmv-miR-m21-1</td><td>2</td><td>-17.8</td><td>3' GCCGAACTTGCACAGGGGATA 5'
    ||| |||||||:
5' -GGCCTCA----GTCCCCTG- 3'</td></tr>
<tr><td>Cxcl12:::NM_001012477</td><td>mcmv-miR-m21-1</td><td>3</td><td>-17.8</td><td>3' GCCGAACTTGCACAGGGGATA 5'
   ||||| :| |:||||:
5' CGGCTGT-GCCC-TTCCCTG- 3'</td></tr>
<tr><td>Ifnar1:::NM_010508</td><td>mcmv-miR-m21-1</td><td>4</td><td>-17.8</td><td>3' GCCGAACTTGC-ACAGGGGATA 5'
         |||| |||||:||:|
5' ------GAACCCTGTCCTCTGT 3'</td></tr>
<tr><td>Ikbkb:::NM_010546</td><td>mcmv-miR-m21-1</td><td>2</td><td>-17.8</td><td>3' GCCGAACT-TGCACAGGGGATA 5'
     |||| || ||||:|||:
5' --GCTTCTCACTTGTCTCCTG- 3'</td></tr>
<tr><td>March8:::NM_027920</td><td>mcmv-miR-m21-1</td><td>1</td><td>-17.8</td><td>3' GCCGAACTTGCACAGGGGATA 5'
           :| ||||||||:|
5' --------GC-TGTCCCCTGT 3'</td></tr>
<tr><td>Mll1:::NM_001081049</td><td>mcmv-miR-m21-1</td><td>2</td><td>-17.71</td><td>3' GCCGAACTTGCACAGGGGATA 5'
    ||||| ||||||:
5' -GGCTT-------TCCCCTG- 3'</td></tr>
<tr><td>Cx3cr1:::NM_009987</td><td>mcmv-miR-m21-1</td><td>1</td><td>-17.6</td><td>3' GCCGAACTTGCACAGGGGATA 5'
     ||||| ||||||:
5' --GCTTGCTTTC-TCCCCTG- 3'</td></tr>
<tr><td>Lat:::NM_010689</td><td>mcmv-miR-m21-1</td><td>1</td><td>-17.4</td><td>3' GCCGAACTTGCAC-AGGGGATA 5'
     || |||: |||||||
5' --GCCTGAGAATCTTCCCCTA- 3'</td></tr>
<tr><td>Samhd1:::NM_018851</td><td>mcmv-miR-m21-1</td><td>1</td><td>-17.4</td><td>3' GCCGAACTTGC-----ACAGGGGATA 5'
    ||: ||| ||||||||:
5' -GGTAC-AACTCCCCCTGTCCCCTG- 3'</td></tr>
<tr><td>Ticam2:::NM_173394</td><td>mcmv-miR-m21-1</td><td>1</td><td>-17.4</td><td>3' GCCGAA---CTTGCACAGGGGATA 5'
     |||| ||| ||||||:||
5' --GCTTTTAGAAA-TGTCCCTTA- 3'</td></tr>
<tr><td>Apc:::NM_007462</td><td>mcmv-miR-m21-1</td><td>4</td><td>-17.3</td><td>3' GCCGAACTTGCACAGGGGATA 5'
    || |||| ||||||
5' -GGAAAGAACCAGCCCCCTA- 3'</td></tr>
<tr><td>Irak1:::NM_008363</td><td>mcmv-miR-m21-1</td><td>1</td><td>-17.3</td><td>3' GCCGAACTTGCAC-AGGGGATA 5'
     |: ||:| ||||||:
5' --GTGGGAGCTAGCTCCCCTG- 3'</td></tr>
<tr><td>H2-K1:::NM_001001892</td><td>mcmv-miR-m21-1</td><td>1</td><td>-17.2</td><td>3' GC-CGAACTTGCACAGGGGATA 5'
    | | ||| |||:||||:|
5' -GTGTCTGA---TGTTCCCTGT 3'</td></tr>
<tr><td>Rsad2:::NM_021384</td><td>mcmv-miR-m21-1</td><td>2</td><td>-17.2</td><td>3' GCCGAACTTGCACAGGGGATA 5'
        ||||| ||||||:|
5' -----TGAAC---TCCCCTGT 3'</td></tr>
<tr><td>Cx3cl1:::NM_009142</td><td>mcmv-miR-m21-1</td><td>2</td><td>-17.1</td><td>3' GCCGAACTTGCACAGGGGATA 5'
             |||||:|||:
5' ----------GTGTCTCCTG- 3'</td></tr>
</table>

Supplementary Table 1. page 3

Gbp5:::NM_153564	mcmv-miR-m21-1	1	-17.1	3' GCCGAACTTGCA-CAGGGGATA 5'<br>| :| ||:||||| 5' ------GGGTGGAGTTCCCTA- 3'
Il10:::NM_010548	mcmv-miR-m21-1	1	-17.1	3' GCCGAACTTGCACAGGGGATA 5'<br>||: || ||||||| 5' ------GAGGGT-TCCCCTA- 3'
Mx2:::NM_013606	mcmv-miR-m21-1	1	-16.7	3' GCCGA-ACTTGCACAGGGGATA 5'<br>|||| || | |:||||:| 5' -GGCTCTGTCC---TTCCCTGT 3'
Kit:::NM_021099	mcmv-miR-m21-1	1	-16.5	3' GCCGAACTTGCAC-AGGGGATA 5'<br>||| ||||||:<br>5' ----------GTGCTCCCCTG- 3'
Hoxb4:::NM_010459	mcmv-miR-m21-1	1	-16.4	3' GCCGAACTTGCACAGGGGATA 5'<br>||||||||| 5' -----------TGTCCCCTA- 3'
Pml:::NM_008884	mcmv-miR-m21-1	1	-16.4	3' GCCGAACTTGCACAGGGGATA 5'<br>||: | |||:||||:| 5' -GGTCAGT---TGTTCCCTGT 3'
Chrna4:::NM_015730	mcmv-miR-m21-1	1	-16.37	3' GCCGAACTTGCACAGGGGATA 5'<br>|||| |||||| 5' -GGCTACCCACTTCCCCTA- 3'
Il25:::NM_080729	mcmv-miR-m21-1	1	-16.3	3' GCCGAACTTGCACAGGGGATA 5'<br>|||| ||||||:<br>5' -GGCTCCTT----TCCCCTG- 3'
Prkca:::NM_011101	mcmv-miR-m21-1	3	-16.3	3' GCCGAACTTGCACAGGGGATA 5'<br>|| :|| ||||:||:<br>5' -GGA---GACAGGTCCTCTG- 3'
Skap1:::NM_001033186	mcmv-miR-m21-1	1	-16.1	3' GCCGAACTTGCACAGGGGATA 5'<br>|||||||| 5' ------------GTCCCCTA- 3'
Irf8:::NM_008320	mcmv-miR-m21-1	1	-16	3' GCCGAACTTGCACAGGGGATA 5'<br>|||||: |:||||:| 5' ---CTTGAGACC-TTCCCTGT 3'
Ttc7:::NM_028639	mcmv-miR-m21-1	2	-16	3' GCCGAACTTGCAC-AGGGGATA 5'<br>||| ||||||:<br>5' ----------GTGCTCCCCTG- 3'
Zbtb16:::NM_001033324	mcmv-miR-m21-1	1	-16	3' GC-CGAACTTGCACAGGGGATA 5'<br>| |||| :| |||||||| 5' -GAGCTTT--TGAATCCCCTAT 3'
4732429D16Rik:::NM_145437	mcmv-miR-m21-1	2	-15.9	3' GCCGAACTTGCACAGGGGATA 5'<br>||| |: ||||||:||| 5' --GCT---ATCTGTCCCTTAT 3'
Sp1:::NM_013672	mcmv-miR-m21-1	1	-15.76	3' GCCGAACTTGCACAGGGGATA 5'<br>||||| |:||||| 5' CGGCTCCATTCC-TTCCCTA- 3'
C3ar1:::NM_009779	mcmv-miR-m21-1	2	-15.4	3' GCCGAACTTGCACAGGGGATA 5'<br>|||:|| || |:|||||| 5' CGGTTT--ACATATTCCCTAT 3'
Inppl1:::NM_010567	mcmv-miR-m21-1	1	-15.4	3' GCCGAACTTG-CACAGGGGATA 5'<br>|| || | ||:||||| 5' -GGG--GATCAGAGTTCCCTA- 3'
Nod2:::NM_145857	mcmv-miR-m21-1	1	-15.4	3' GCCGAACTTGCAC-AGGGGATA 5'<br>||: |:| ||||||:<br>5' -GGTCA-AGCAGATTCCCCTG- 3'
Tgtp:::NM_011579	mcmv-miR-m21-1	1	-15.3	3' GCCGAACTTGCACAGGGGATA 5'<br>||| ||||||| 5' -GGC---------TCCCCTA- 3'
Ndrg1:::NM_008681	mcmv-miR-m21-1	1	-15.1	3' GCCGAACTTGCACAGGGGATA 5'<br>|| |||| ||||:|:|| 5' --GC-TGAA--TGTCTCTTA- 3'
H2-Q7:::NM_010394	mcmv-miR-m21-1	1	-14.9	3' GCCGAACTT-GCACAGGGGATA 5'<br>|| | |||:||||:| 5' -----TGTCTCATGTTCCCTGT 3'
Il1a:::NM_010554	mcmv-miR-m21-1	1	-14.8	3' GCCGAACTTGCAC--AGGGGATA 5'<br>||| ||||||:<br>5' ----------GTGTTTCCCCTG- 3'
Oasl2:::NM_011854	mcmv-miR-m21-1	1	-14.7	3' GCCGAACTTGCAC--------AGGGGATA 5'<br>||:|||| ||||:|:| 5' -----TGGACGTAAATCTATCTCCCTTGT 3'
Slc7a2:::NM_001044740	mcmv-miR-m21-1	2	-14.7	3' GCCGAACTTGCAC-AGGGGATA 5'<br>|| ||:| ||||||:| 5' ---CTGAAATGCCATCCCCTGT 3'
Tnfsf15:::NM_177371	mcmv-miR-m21-1	1	-14.7	3' GCCGAACTTGCACAGGGGATA 5'<br>||| ||||||:<br>5' -GGC---------TCCCCTG- 3'
Tal1:::NM_011527	mcmv-miR-m21-1	3	-14.6	3' GCCGAACTTGC--ACAGGGGATA 5'<br>||| ||||:||||| 5' -----TGATTCTTTGTCTCCTAT 3'
Skil:::NM_001039090	mcmv-miR-m21-1	1	-14.5	3' GCCGAACTTGCACAGGGGATA 5'<br>||| |||||:||| 5' -------AAC-TGTCCTCTA- 3'
Heph:::NM_181273	mcmv-miR-m21-1	1	-14.4	3' GCCGAACTTGCAC-AGGGGATA 5'<br>||||:| |:||||:<br>5' ----TTGAGCATATTTCCCTG- 3'
Jarid2:::NM_021878	mcmv-miR-m21-1	1	-14.4	3' GCCGAACTTGCAC-AGGGGATA 5'<br>|| ||| |:||||:| 5' -----TGTAAGTGCTTCCCTGT 3'
Clec2i:::NM_020257	mcmv-miR-m21-1	1	-14.34	3' GC-CGAACTTGCACAGGGGATA 5'<br>| ||| |||||||| 5' -GTGCTACTTTT--TCCCCTAT 3'
Cxcl16:::NM_023158	mcmv-miR-m21-1	10	-14.3	3' GCC-GAACTTGCACAGGGGATA 5'<br>|| || |:| |:|||||| 5' -GGTCTCCAGCC--TTCCCTAT 3'
Ifit2:::NM_008332	mcmv-miR-m21-1	1	-14.2	3' GCCGA-ACTTGCACAGGGGATA 5'<br>||| ||: ||||||:<br>5' --GCTCTGGT----TCCCCTG- 3'
Abcc9:::NM_001044720	mcmv-miR-m21-1	1	-14.1	3' GCCGAACTTGCAC--AGGGGATA 5'<br>|||| || ||||||:<br>5' -----TGAAGTTGCCTCCCCTG- 3'

Supplementary Table 1. page 4

<table>
<tr><td>H2-Q8:::NM_023124</td><td>mcmv-miR-m21-1</td><td>1</td><td>-14.1</td><td>3' GCCGAACTTGCACAGGGGATA 5'
             || ||||||:|
5' ----------GT-TCCCCTGT 3'</td></tr>
<tr><td>H28:::NM_031367</td><td>mcmv-miR-m21-1</td><td>1</td><td>-14</td><td>3' GCCGAACTTGCACAGGGGATA 5'
               ||||:||:|
5' ------------GTCCTCTGT 3'</td></tr>
<tr><td>Masp1:::NM_008555</td><td>mcmv-miR-m21-1</td><td>1</td><td>-14</td><td>3' GCCGAACTTGCAC--AGGGGATA 5'
       |||||| |:||||:
5' ----TTGAACCACCTTTCCCTG- 3'</td></tr>
<tr><td>Orc3l:::NM_015824</td><td>mcmv-miR-m21-1</td><td>1</td><td>-14</td><td>3' GCCGAACTTGCAC-AGGGGATA 5'
          ||| || |||||||
5' -------AAC-TGCTCCCCTA- 3'</td></tr>
<tr><td>Pik3cd:::NM_001029837</td><td>mcmv-miR-m21-1</td><td>1</td><td>-13.9</td><td>3' GCCGAACTTGCACAGGGGATA 5'
         || : ||||||:||
5' ------GACTCTGTCCCTTA- 3'</td></tr>
<tr><td>Dclre1c:::NM_175683</td><td>mcmv-miR-m21-1</td><td>1</td><td>-13.8</td><td>3' GCCGAACTTGCACAGGGGATA 5'
     |||| ||||||:
5' --GCTTTTTT---TCCCCTG- 3'</td></tr>
<tr><td>Cd74:::NM_001042605</td><td>mcmv-miR-m21-1</td><td>1</td><td>-13.6</td><td>3' GCCGAACTTGCACAGGGGATA 5'
              |||:||||:
5' -----------TGTTCCCTG- 3'</td></tr>
<tr><td>Il1r1:::NM_008362</td><td>mcmv-miR-m21-1</td><td>1</td><td>-13.6</td><td>3' GCCGAACTTGCACAGGGGATA 5'
               |||||:|:|
5' ------------GTCCCTTGT 3'</td></tr>
<tr><td>Txnrd2:::NM_013711</td><td>mcmv-miR-m21-1</td><td>1</td><td>-13.6</td><td>3' GCCGAACTTGCACAGGGGATA 5'
    || ||| ||||||
5' -GGGATGACTATA-CCCCTA- 3'</td></tr>
<tr><td>B4galt1:::NM_022305</td><td>mcmv-miR-m21-1</td><td>1</td><td>-13.4</td><td>3' GCCGAACTTGCACAGGGGATA 5'
               |||||:|:|
5' ------------GTCCCTTGT 3'</td></tr>
<tr><td>Cd8a:::NM_001081110</td><td>mcmv-miR-m21-1</td><td>2</td><td>-13.4</td><td>3' GCCGAACTTGCAC-AGGGGATA 5'
      ||||| ||:|||:|
5' ---CTTGATATAAATCTCCTGT 3'</td></tr>
<tr><td>Nfam1:::NM_028728</td><td>mcmv-miR-m21-1</td><td>2</td><td>-13.4</td><td>3' GCCGAACTTGCACAGGGGATA 5'
            || ||||||:
5' ---------CGCCTCCCCTG- 3'</td></tr>
<tr><td>C8a:::NM_146148</td><td>mcmv-miR-m21-1</td><td>2</td><td>-13.3</td><td>3' GCCGAACTTGCACAGGGGATA 5'
          :|| ||||||:|
5' -------GACCA-TCCCCTGT 3'</td></tr>
<tr><td>Crip3:::NM_053250</td><td>mcmv-miR-m21-1</td><td>1</td><td>-13.3</td><td>3' GCCGAACTTGCACAGGGGATA 5'
             | ||:||||:
5' ----------GCGTTCCCTG- 3'</td></tr>
<tr><td>Ddx58:::NM_172689</td><td>mcmv-miR-m21-1</td><td>1</td><td>-13.3</td><td>3' GCCGAACTTGCACAGGGGATA 5'
              ||||||:|:
5' -----------TGTCCCTTG- 3'</td></tr>
<tr><td>Bcl3:::NM_033601</td><td>mcmv-miR-m21-1</td><td>1</td><td>-13.2</td><td>3' GCCGAACTTGCACAGGGGATA 5'
               ||:||||:|
5' ------------GTTCCCTGT 3'</td></tr>
<tr><td>Tgfbr2:::NM_009371</td><td>mcmv-miR-m21-1</td><td>1</td><td>-13.2</td><td>3' GCCGAACTTGCACAGGGGATA 5'
              |||:||||:
5' -----------TGTTCCCTG- 3'</td></tr>
<tr><td>Tnfsf11:::NM_011613</td><td>mcmv-miR-m21-1</td><td>1</td><td>-13.1</td><td>3' GC-CGAACTTGCAC--AGGGGATA 5'
    | || :|| ||||||:
5' -GTGCACTT-TGTAATTCCCCTG- 3'</td></tr>
<tr><td>Cxcl9:::NM_008599</td><td>mcmv-miR-m21-1</td><td>1</td><td>-12.9</td><td>3' GCCGAACTTGCACAGGGGATA 5'
          :|| |||||||
5' -------GACTATTCCCCTA- 3'</td></tr>
<tr><td>Aicda:::NM_009645</td><td>mcmv-miR-m21-1</td><td>1</td><td>-12.8</td><td>3' GCCGAAC-TTGCACAGGGGATA 5'
         | |:| ||||||:
5' ------GAAGCAAATCCCCTG- 3'</td></tr>
<tr><td>Dnaja3:::NM_023646</td><td>mcmv-miR-m21-1</td><td>1</td><td>-12.8</td><td>3' GCCGAACTTGCACAGGGGATA 5'
               ||||:||:
5' ------------GTCCTCTG- 3'</td></tr>
<tr><td>Tbkbp1:::NM_198100</td><td>mcmv-miR-m21-1</td><td>1</td><td>-12.8</td><td>3' GCCGAACTTGCACAGGGGATA 5'
            ||| |:||||:
5' ---------CGTCTTCCCTG- 3'</td></tr>
<tr><td>Add1:::NM_001102444</td><td>mcmv-miR-m21-1</td><td>1</td><td>-12.7</td><td>3' GCCGAACTTGCACAGGGGATA 5'
        ||| | ||||||:
5' -----TGACCTC-TCCCCTG- 3'</td></tr>
<tr><td>Cd247:::NM_031162</td><td>mcmv-miR-m21-1</td><td>1</td><td>-12.7</td><td>3' GCCGAACTTGCAC---AGGGGATA 5'
         ||| ||||||:
5' ------GAAACCACCCTCCCCTG- 3'</td></tr>
<tr><td>Indo:::NM_008324</td><td>mcmv-miR-m21-1</td><td>1</td><td>-12.7</td><td>3' GCCGAACTTGCAC--AGGGGATA 5'
             ||| |:||||:|
5' ----------GTGCATTCCCTGT 3'</td></tr>
<tr><td>Ciapin1:::NM_134141</td><td>mcmv-miR-m21-1</td><td>1</td><td>-12.5</td><td>3' GCCGAACTTGCACAGGGGATA 5'
                ||||||:|
5' -------------TCCCCTGT 3'</td></tr>
<tr><td>Itpkb:::NM_001081175</td><td>mcmv-miR-m21-1</td><td>1</td><td>-12.43</td><td>3' GCCGAACTTGCACAGGGGATA 5'
     || |||||||
5' --GCCTCTCTCC-TCCCCTA- 3'</td></tr>
<tr><td>Irgm:::NM_008326</td><td>mcmv-miR-m21-1</td><td>1</td><td>-12.4</td><td>3' GCCGAACTTGCACAGGGGATA 5'
                ||||||:|
5' -------------TCCCCTGT 3'</td></tr>
<tr><td>Sod2:::NM_013671</td><td>mcmv-miR-m21-1</td><td>1</td><td>-12.4</td><td>3' GCCGAACTTGCACAGGGGATA 5'
                |||||||
5' -------------TCCCCTA- 3'</td></tr>
<tr><td>Tlr7:::NM_133211</td><td>mcmv-miR-m21-1</td><td>1</td><td>-12.4</td><td>3' GCCGAACTTGCACAGGGGATA 5'
                |||||||
5' -------------TCCCCTA- 3'</td></tr>
<tr><td>Cebpg:::NM_009884</td><td>mcmv-miR-m21-1</td><td>1</td><td>-12.3</td><td>3' GCCGAACTTGCACAGGGGATA 5'
    ||:| ||:|||:|
5' -GGTT--------TCTCCTGT 3'</td></tr>
<tr><td>Ifnz:::NM_197889</td><td>mcmv-miR-m21-1</td><td>1</td><td>-12.3</td><td>3' GCCGAACTTGCACAGGGGATA 5'
      ||| |:|| |:||||:
5' ---CTTT-ATGTCTTCCCTG- 3'</td></tr>
<tr><td>Sla2:::NM_029983</td><td>mcmv-miR-m21-1</td><td>1</td><td>-12.24</td><td>3' GCCGAACTTGCACAGGGGATA 5'
    ||| ||||||
5' -GGCCTCA------CCCCTA- 3'</td></tr>
</table>

Supplementary Table 1. page 5

<table>
<tr><td>Ccl22:::NM_009137</td><td>mcmv-miR-m21-1</td><td>1</td><td>-12.2</td><td>3' GCCGAACTTGCACAGGGGATA 5'
                ||||||:
5' -------------TCCCCTG- 3'</td></tr>
<tr><td>Ccl5:::NM_013653</td><td>mcmv-miR-m21-1</td><td>1</td><td>-12.2</td><td>3' GCCGAACTTGCACAGGGGATA 5'
                |||||||
5' -------------TCCCCTA- 3'</td></tr>
<tr><td>Gbp1:::NM_010259</td><td>mcmv-miR-m21-1</td><td>1</td><td>-12.2</td><td>3' GCCGAACTTGCACAGGGGATA 5'
                |||||||
5' -------------TCCCCTA- 3'</td></tr>
<tr><td>Klrk1:::NM_001083322</td><td>mcmv-miR-m21-1</td><td>1</td><td>-12.2</td><td>3' GCCGAACTTGCACAGGGGATA 5'
                |||||||
5' -------------TCCCCTA- 3'</td></tr>
<tr><td>Myd88:::NM_010851</td><td>mcmv-miR-m21-1</td><td>1</td><td>-12.2</td><td>3' GCCGAACTTGCACAGGGGATA 5'
                |||||||
5' -------------TCCCCTA- 3'</td></tr>
<tr><td>Rag1:::NM_009019</td><td>mcmv-miR-m21-1</td><td>1</td><td>-12.1</td><td>3' GCCGAACTTGCACAGGGGATA 5'
                ||||||:
5' -------------TCCCCTG- 3'</td></tr>
<tr><td>Il20rb:::NM_001033543</td><td>mcmv-miR-m21-1</td><td>1</td><td>-12</td><td>3' GCCGAACTTGCAC--AGGGGATA 5'
         |:|| |||:||:|
5' ------GGACTTCATTCCTCTGT 3'</td></tr>
<tr><td>Il2ra:::NM_008367</td><td>mcmv-miR-m21-1</td><td>1</td><td>-12</td><td>3' GCCGAACTTGCACAGGGGATA 5'
                |||||||
5' -------------TCCCCTA- 3'</td></tr>
<tr><td>Il31ra:::NM_139299</td><td>mcmv-miR-m21-1</td><td>1</td><td>-11.92</td><td>3' GCCGAACTTGCACAGGGGATA 5'
    ||| |||:||:|
5' -GGCCATT-----TCCTCTGT 3'</td></tr>
<tr><td>Smad5:::NM_008541</td><td>mcmv-miR-m21-1</td><td>1</td><td>-11.9</td><td>3' GCCGAACTTGCACAGGGGATA 5'
                ||||||:
5' -------------TCCCCTG- 3'</td></tr>
<tr><td>Ccr2:::NM_009915</td><td>mcmv-miR-m21-1</td><td>2</td><td>-11.8</td><td>3' GCCGAACTTGCAC-AGGGGATA 5'
          ||:|| |:||||:|
5' -------AATGTTCTTCCCTGT 3'</td></tr>
<tr><td>Orai1:::NM_175423</td><td>mcmv-miR-m21-1</td><td>1</td><td>-11.7</td><td>3' GCCGAACTTGCACAGGGGATA 5'
                ||||||:
5' -------------TCCCCTG- 3'</td></tr>
<tr><td>Selplg:::NM_009151</td><td>mcmv-miR-m21-1</td><td>1</td><td>-11.7</td><td>3' GCCGAACTTGCACAGGGGATA 5'
                ||||||:
5' -------------TCCCCTG- 3'</td></tr>
<tr><td>Tlx1:::NM_021901</td><td>mcmv-miR-m21-1</td><td>1</td><td>-11.7</td><td>3' GCCGAACTTGCACAGGGGATA 5'
                ||||||:
5' -------------TCCCCTG- 3'</td></tr>
<tr><td>Elf4:::NM_019680</td><td>mcmv-miR-m21-1</td><td>1</td><td>-11.6</td><td>3' GCCGAACTTGCACA-GGGGATA 5'
         | ||| :|||||
5' ------GTCCGTCCCTCCCTA- 3'</td></tr>
<tr><td>Myo1e:::NM_181072</td><td>mcmv-miR-m21-1</td><td>1</td><td>-11.6</td><td>3' GCCGAACTTGCACAGGGGATA 5'
        ||| |||:||:|
5' -----TGATACATTCCTCTGT 3'</td></tr>
<tr><td>Pag1:::NM_053182</td><td>mcmv-miR-m21-1</td><td>1</td><td>-11.46</td><td>3' GCCGAACTTGCACAGGGGATA 5'
     ||| |:||||:|
5' --GCTATTTTCC-TTCCCTGT 3'</td></tr>
<tr><td>Rhoh:::NM_001081105</td><td>mcmv-miR-m21-1</td><td>1</td><td>-11.44</td><td>3' GCCGAACTTGCACAGGGGATA 5'
     ||| |||:||:|
5' --GCTGTTTTC--TCCTCTGT 3'</td></tr>
<tr><td>Atp7a:::NM_009726</td><td>mcmv-miR-m21-1</td><td>1</td><td>-11.3</td><td>3' GCCGAACTTGCACAGGGGATA 5'
        || |||||::||
5' -----TGTCATTGTCCTTTA- 3'</td></tr>
<tr><td>Exo1:::NM_012012</td><td>mcmv-miR-m21-1</td><td>1</td><td>-11.2</td><td>3' GCCGAACTTGCACAGGGGATA 5'
                 ||||||
5' --------------CCCCTA- 3'</td></tr>
<tr><td>Irf4:::NM_013674</td><td>mcmv-miR-m21-1</td><td>1</td><td>-11.2</td><td>3' GCCGAACTTGCACAGGGGATA 5'
      |||| | |:|||||
5' ---CTTGCCC---TTCCCTA- 3'</td></tr>
<tr><td>Epas1:::NM_010137</td><td>mcmv-miR-m21-1</td><td>4</td><td>-11.1</td><td>3' GCCGAACTTGCACAGGGGATA 5'
                 ||||||
5' --------------CCCCTA- 3'</td></tr>
<tr><td>Bak1:::NM_007523</td><td>mcmv-miR-m21-1</td><td>1</td><td>-10.9</td><td>3' GCCGAACTTGCACAGGGGATA 5'
                 ||||||
5' --------------CCCCTA- 3'</td></tr>
<tr><td>Hoxb3:::NM_001079869</td><td>mcmv-miR-m21-1</td><td>1</td><td>-10.9</td><td>3' GCCGAACTTGCACAGGGGATA 5'
                 ||||||
5' --------------CCCCTA- 3'</td></tr>
<tr><td>Icosl:::NM_015790</td><td>mcmv-miR-m21-1</td><td>1</td><td>-10.9</td><td>3' GCCGAACTTGCACAGGGGATA 5'
    || :|| |:||||:
5' -GGA---GACT--TTCCCTG- 3'</td></tr>
<tr><td>Bcl6:::NM_009744</td><td>mcmv-miR-m21-1</td><td>1</td><td>-10.8</td><td>3' GCCGAACTTGCACAGGGGATA 5'
     |||| ||:|||||
5' --GCTTCT-----TCTCCTAT 3'</td></tr>
<tr><td>Tnfsf12:::NM_011614</td><td>mcmv-miR-m21-1</td><td>1</td><td>-10.7</td><td>3' GCCGAACTTGCACAGGGGATA 5'
      ||| ||| |:||||:
5' ---CTTAAACT--TTCCCTG- 3'</td></tr>
<tr><td>Eif2ak2:::NM_011163</td><td>mcmv-miR-m21-1</td><td>1</td><td>-10.6</td><td>3' GCCGAACTTGCAC-AGGGGATA 5'
         ||| |||:||:
5' ------GAAAACATTCCTCTG- 3'</td></tr>
<tr><td>Fancc:::NM_007985</td><td>mcmv-miR-m21-1</td><td>1</td><td>-10.6</td><td>3' GCCGAACTTGCAC-AGGGGATA 5'
              || |:||||||
5' -----------TGCTTCCCTAT 3'</td></tr>
<tr><td>Il1rl1:::NM_010743</td><td>mcmv-miR-m21-1</td><td>1</td><td>-10.5</td><td>3' GCCGAACTTGCAC-AGGGGATA 5'
          ||:| |||:||:|
5' -------AATGCCTTCCTCTGT 3'</td></tr>
<tr><td>Cxcr4:::NM_009911</td><td>mcmv-miR-m21-1</td><td>1</td><td>-10.3</td><td>3' GCCGAACTTGCACAGGGGATA 5'
       |||| :|||||
5' ----TTGATTTCACTCCCTA- 3'</td></tr>
<tr><td>Rogdi:::NM_133185</td><td>mcmv-miR-m21-1</td><td>1</td><td>-10.3</td><td>3' GCCGAACTTGCACAGGGGATA 5'
                |:||||:|
5' -------------TTCCCTGT 3'</td></tr>
<tr><td>Sod1:::NM_011434</td><td>mcmv-miR-m21-1</td><td>1</td><td>-10.3</td><td>3' GCCGAACTTGCACAGGGGATA 5'
                |:||||:|
5' -------------TTCCCTGT 3'</td></tr>
</table>

<table>
<tr><td>H2-Eb2:::NM_001033978</td><td>mcmv-miR-m21-1</td><td>1</td><td>-10.1</td><td>3' GCCGAACTTGCACAGGGGATA 5'
|:||||:|
5' -------------TTCCCTGT 3'</td></tr>
<tr><td>Pbx1:::NM_183355</td><td>mcmv-miR-m21-1</td><td>1</td><td>-10.1</td><td>3' GCCGAACTTGCACAGGGGATA 5'
|:||||:|
5' -------------TTCCCTGT 3'</td></tr>
</table>

miR-M23-2 predicted targets ranked by minimal free energy (MFE) of binding

<table>
<tr><th>Predicted Target</th><th></th><th></th><th colspan="2">Binding site with minimal free energy (MFE)</th></tr>
<tr><th>Gene symbol:::RefSeq</th><th>miRNA</th><th># binding sites</th><th>MFE (Kcal/mol)</th><th>Binding site</th></tr>
<tr><td>Gm106:::NM_001033288</td><td>mcmv-miR-M23-2</td><td>1</td><td>-30.7</td><td>3' GGCGAACTGGCTCCGGGGGTA 5'
|||| ||||||||:|||
5' --GCTT--CCGAGGCCTCCA- 3'</td></tr>
<tr><td>Plekhk1:::NM_001081346</td><td>mcmv-miR-M23-2</td><td>1</td><td>-29</td><td>3' GGCGAACTGGCTCC-GGGGGTA 5'
:||||| || ||| ||||||
5' -TGCTTG-CCCAGGACCCCCA- 3'</td></tr>
<tr><td>Ccl22:::NM_009137</td><td>mcmv-miR-M23-2</td><td>2</td><td>-27.5</td><td>3' GGCGAACTGGCTCCGGGGGTA 5'
|:| |:|| |||:|||||
5' --GTTAGGCCAAGGTCCCCA- 3'</td></tr>
<tr><td>Tinagl:::NM_023476</td><td>mcmv-miR-M23-2</td><td>1</td><td>-27.4</td><td>3' GGCGAACTGGCTCCGGGGGTA 5'
|| ||:| || |||||||
5' --GC-TGGCGGAAGCCCCCA- 3'</td></tr>
<tr><td>Nfkb1:::NM_008689</td><td>mcmv-miR-M23-2</td><td>2</td><td>-27.2</td><td>3' GGCGAACTGGCTCCGGGGGTA 5'
|:|| ||:||| ||||||
5' CTGC-TGGCCGTT-CCCCCA- 3'</td></tr>
<tr><td>Icosl:::NM_015790</td><td>mcmv-miR-M23-2</td><td>2</td><td>-26.4</td><td>3' GGCGAACT-GG-C-TCCGGGGGTA 5'
||| || | |||||||||
5' --GCTGATTCCAGCAGGCCCCCA- 3'</td></tr>
<tr><td>Ncf1:::NM_010876</td><td>mcmv-miR-M23-2</td><td>1</td><td>-26.1</td><td>3' GGCGAACTGG-CTCCGGGGGTA 5'
||||: |:||:|||||
5' -----TGACTGGGGGTCCCCA- 3'</td></tr>
<tr><td>Jak2:::NM_001048177</td><td>mcmv-miR-M23-2</td><td>1</td><td>-26</td><td>3' GGCGAACTGGCTCCGGGGGTA 5'
|:|: ||| ||||||:|||
5' CTGTGTGA--GAGGCCTCCA- 3'</td></tr>
<tr><td>Tal1:::NM_011527</td><td>mcmv-miR-M23-2</td><td>1</td><td>-25.4</td><td>3' GGCGAAC-TGGCT-CCGGGGGTA 5'
| ||:|| ||||||||
5' ------GTACTGAGGGCCCCCA- 3'</td></tr>
<tr><td>Myh9:::NM_022410</td><td>mcmv-miR-M23-2</td><td>3</td><td>-25.2</td><td>3' GGCGA-ACTGGCTCCGGGGGTA 5'
|:||| ||||| | |||||
5' CTGCTCTGACCATGTCCCCC-- 3'</td></tr>
<tr><td>Tcfeb:::NM_011549</td><td>mcmv-miR-M23-2</td><td>2</td><td>-25.2</td><td>3' GGCGAACTGGCTCCGGGGGTA 5'
|| ||||:|:| ||||||
5' CC-CTTGGCTGCTACCCCCA- 3'</td></tr>
<tr><td>Zbtb22:::NM_020625</td><td>mcmv-miR-M23-2</td><td>1</td><td>-24.7</td><td>3' GGCGAACTGGCTCC---GGGGGTA 5'
||| |||:|| ||||||
5' --GCTC--CCGGGGTTTCCCCCA- 3'</td></tr>
<tr><td>Bcl2l11:::NM_009754</td><td>mcmv-miR-M23-2</td><td>2</td><td>-24.1</td><td>3' GGCGAACTG-GCT--CCGGGGGTA 5'
|||| :| ||||||||
5' -----TGACATGTGTGGCCCCCA- 3'</td></tr>
<tr><td>Dock2:::NM_033374</td><td>mcmv-miR-M23-2</td><td>1</td><td>-24</td><td>3' GGCGAACTGGCTCCGGGGGTA 5'
|:||||||||
5' ----------GGGGCCCCCA- 3'</td></tr>
<tr><td>Tlx1:::NM_021901</td><td>mcmv-miR-M23-2</td><td>1</td><td>-24</td><td>3' GGCGAACT-GGC--TCCGGGGGTA 5'
||||: |:| ||||||:||
5' ---CTTGGTCTGTCAGGCCCTCA- 3'</td></tr>
<tr><td>Tnfrsf13c:::NM_028075</td><td>mcmv-miR-M23-2</td><td>1</td><td>-24</td><td>3' GGCGAAC-TGGCTCCGGGGGTA 5'
|:|:| :|||||| |:|||
5' CTGTTCTAGCCGAGGACTCCA- 3'</td></tr>
<tr><td>Eda:::NM_010099</td><td>mcmv-miR-M23-2</td><td>1</td><td>-23.9</td><td>3' GGCGAACTGGCTCCGGGGGTA 5'
:|| |:| |||||||||
5' -TGCA-GGC--AGGCCCCCA- 3'</td></tr>
<tr><td>March8:::NM_027920</td><td>mcmv-miR-M23-2</td><td>2</td><td>-23.9</td><td>3' GGCGAACTGGCT-CCGGGGGTA 5'
|||: || ||:|||||
5' -----TGATGGAGGGTCCCCA- 3'</td></tr>
<tr><td>Card11:::NM_175362</td><td>mcmv-miR-M23-2</td><td>2</td><td>-23.5</td><td>3' GGCGAACTGGC-TCCGGGGGTA 5'
|:|| |:| |||||:|||
5' CTGC----CTGCAGGCCTCCA- 3'</td></tr>
<tr><td>Hdac5:::NM_001077696</td><td>mcmv-miR-M23-2</td><td>1</td><td>-23.4</td><td>3' GGCGAACTGGCTCCGGGGGTA 5'
||| |||||||||
5' -------ACCCTGGCCCCCAT 3'</td></tr>
<tr><td>Sox4:::NM_009238</td><td>mcmv-miR-M23-2</td><td>3</td><td>-23.4</td><td>3' GGCGAACTGGCTCC----GGGGGTA 5'
|||||| ||| ||||||
5' --GCTTGAA-GAGACTCCCCCCCA- 3'</td></tr>
<tr><td>Tgfbr2:::NM_009371</td><td>mcmv-miR-M23-2</td><td>6</td><td>-23.3</td><td>3' GGCGAACTGGCTCCGGGGGTA 5'
||| |:|| |||:|||
5' --GCTGGGCCAA-GCCTCCA- 3'</td></tr>
<tr><td>A830091I15Rik:::NM_172795</td><td>mcmv-miR-M23-2</td><td>2</td><td>-23.2</td><td>3' GGCGAACTGGCTCCGGGGGTA 5'
||:| |||:|||||
5' ------GATC-AGGTCCCCA- 3'</td></tr>
<tr><td>Spn:::NM_009259</td><td>mcmv-miR-M23-2</td><td>1</td><td>-23.1</td><td>3' GGCGAACTGGCTCCGGGGGTA 5'
|: || |:| |||||||
5' --GTGTGTCTGCAGCCCCCA- 3'</td></tr>
<tr><td>H2-Eb1:::NM_010382</td><td>mcmv-miR-M23-2</td><td>1</td><td>-22.8</td><td>3' GGCGAACTGGCTCC--GGGGGTA 5'
|||||:|| ||:|||
5' --GCTTGGCCCACATCCCTCCA- 3'</td></tr>
<tr><td>Epas1:::NM_010137</td><td>mcmv-miR-M23-2</td><td>2</td><td>-22.7</td><td>3' GGCGAACTGGCTC---CGGGGGTA 5'
|| || |:|: ||||||||
5' --GC-TGTCTGGTAATGCCCCCAT 3'</td></tr>
<tr><td>C1qc:::NM_007574</td><td>mcmv-miR-M23-2</td><td>1</td><td>-22.6</td><td>3' GGCGAACTGGCTCCGGGGGTA 5'
|:|||| || |||||||
5' CTGCTT--CCA--GCCCCCA- 3'</td></tr>
<tr><td>C8b:::NM_133882</td><td>mcmv-miR-M23-2</td><td>1</td><td>-22.6</td><td>3' GGCGAACTGGCTCCGGGGGTA 5'
| ||| || ||||::|||
5' -CACTTAGCC-AGGCTTCCA- 3'</td></tr>
</table>

Supplementary Table 1. page 7

<table>
<tr><td>Lyst:::NM_010748</td><td>mcmv-miR-M23-2</td><td>1</td><td>-22.5</td><td>3' GGCGAACTGGCTCCGGGGGTA 5'
     ||||| || |||:||:|
5' -CGCTTCCCCA--GCCTCCGT 3'</td></tr>
<tr><td>Ticam1:::NM_174989</td><td>mcmv-miR-M23-2</td><td>1</td><td>-22.5</td><td>3' GGCGAACTGGCTCCGGGGGTA 5'
           :|| :||:|||||
5' -------GCCTGGGTCCCCA- 3'</td></tr>
<tr><td>Cd7:::NM_009854</td><td>mcmv-miR-M23-2</td><td>2</td><td>-22.41</td><td>3' GGCGAACTGGCTCCGGGGGTA 5'
    |:||| ||||||||
5' CTGCT-------GGCCCCCA- 3'</td></tr>
<tr><td>H2-Q8:::NM_023124</td><td>mcmv-miR-M23-2</td><td>1</td><td>-22.4</td><td>3' GGCGAACTGGCTCCGGGGGTA 5'
     | || |:|:|: ||||||
5' -CACTAGGCTGGTTCCCCCA- 3'</td></tr>
<tr><td>Vasn:::NM_139307</td><td>mcmv-miR-M23-2</td><td>1</td><td>-22.4</td><td>3' GGCGAACTGGCTCCGGGGGTA 5'
    || ||| || |||||||
5' CCAG-TGAAGGAAGCCCCCA- 3'</td></tr>
<tr><td>Cblb:::NM_001033238</td><td>mcmv-miR-M23-2</td><td>3</td><td>-22.3</td><td>3' GGCGAACTGGCTC--CGGGGGTA 5'
    |:||| |:| ||||||||
5' CTGCT---CTGTGTTGCCCCCAT 3'</td></tr>
<tr><td>Cd300a:::NM_170758</td><td>mcmv-miR-M23-2</td><td>4</td><td>-22.3</td><td>3' GGCGAACTGGCTCCGGGGGTA 5'
      || || | ||:|||||
5' --GCCCGAGCTTGGTCCCCA- 3'</td></tr>
<tr><td>Smad3:::NM_016769</td><td>mcmv-miR-M23-2</td><td>2</td><td>-22.3</td><td>3' GGCGAAC-TGGCTCCGGGGGTA 5'
    || || ||||| ||||||
5' CCACTGACACCGA--CCCCCA- 3'</td></tr>
<tr><td>Zbtb16:::NM_001033324</td><td>mcmv-miR-M23-2</td><td>3</td><td>-22.3</td><td>3' GGCGAACTGGCTCCGGGGGTA 5'
          |:|| ||||||||
5' ------GGCCAC-GCCCCCAT 3'</td></tr>
<tr><td>Smad5:::NM_008541</td><td>mcmv-miR-M23-2</td><td>1</td><td>-22.2</td><td>3' GGCGAACTGGCTCCGGGGGTA 5'
     :||||| || ||||||
5' -TGCTTG-CCCTT-CCCCCA- 3'</td></tr>
<tr><td>Rorc:::NM_011281</td><td>mcmv-miR-M23-2</td><td>2</td><td>-21.8</td><td>3' GGCGAACTGGCTCCGGGGGTA 5'
    |:| ||:| |||||||
5' CTGG-TGGCAA--GCCCCCA- 3'</td></tr>
<tr><td>Cplx2:::NM_009946</td><td>mcmv-miR-M23-2</td><td>5</td><td>-21.6</td><td>3' GGCGAACTGGCTCCGGGGGTA 5'
      ||| :|| || ||||||
5' --GCT--GCCCAGACCCCCA- 3'</td></tr>
<tr><td>Plcg2:::NM_172285</td><td>mcmv-miR-M23-2</td><td>2</td><td>-21.6</td><td>3' GGCGAACTGGCTCC---GGGGGTA 5'
         |||: |||| ||||||
5' -----TGATAGAGGATCCCCCCA- 3'</td></tr>
<tr><td>Relb:::NM_009046</td><td>mcmv-miR-M23-2</td><td>1</td><td>-21.5</td><td>3' GGCGAACTGGCTCCGGGGGTA 5'
      | || | |||:|||||
5' --GAGGGAGC-AGGTCCCCA- 3'</td></tr>
<tr><td>Blr1:::NM_007551</td><td>mcmv-miR-M23-2</td><td>1</td><td>-21.4</td><td>3' GGCGAACTGGCTC-CGGGGGTA 5'
          |:|| || |:|||||
5' ------GGCC-AGTGTCCCCA- 3'</td></tr>
<tr><td>Rpl22:::NM_009079</td><td>mcmv-miR-M23-2</td><td>1</td><td>-21.4</td><td>3' GGCGAACTGGCTCCGGGGGTA 5'
    |:|:||| |: |||:|||
5' CTGTTTGTCTA--GCCTCCA- 3'</td></tr>
<tr><td>Thpo:::NM_009379</td><td>mcmv-miR-M23-2</td><td>1</td><td>-21.4</td><td>3' GGCGAACTGGCTCCGGGGGTA 5'
    |||| || ||||||
5' CCGCGCCTCCAACACCCCCA- 3'</td></tr>
<tr><td>Traf6:::NM_009424</td><td>mcmv-miR-M23-2</td><td>3</td><td>-21.4</td><td>3' GGCGAACTGGCTCCGGGGGTA 5'
    || ||| |:| |||:|||
5' CC-CTTAGCTGT-GCCTCCA- 3'</td></tr>
<tr><td>9830130M13Rik:::NM_177713</td><td>mcmv-miR-M23-2</td><td>2</td><td>-21.3</td><td>3' GGCGAACTGGCTCCGGGGGTA 5'
    || | ||| |||||:||:
5' CC-CATCACC-AGGCCTCCG- 3'</td></tr>
<tr><td>Chrnb2:::NM_009602</td><td>mcmv-miR-M23-2</td><td>3</td><td>-21.3</td><td>3' GGCGAACTGGCTCCGGGGGTA 5'
           :|||| |:|||||
5' -------GCCGA-GTCCCCA- 3'</td></tr>
<tr><td>Cx3cr1:::NM_009987</td><td>mcmv-miR-M23-2</td><td>2</td><td>-21.3</td><td>3' GGCGAACTGGCTCC-----GGGGGTA 5'
          |||:||| ||||||
5' ------GACTGAGTCATATCCCCCA- 3'</td></tr>
<tr><td>Fcer1g:::NM_010185</td><td>mcmv-miR-M23-2</td><td>1</td><td>-21.3</td><td>3' GGCGAACTGGCTCCGGGGGTA 5'
     | | ||:| |||:||||||
5' -CAC-TGGC--AGGTCCCCAT 3'</td></tr>
<tr><td>Ihh:::NM_010544</td><td>mcmv-miR-M23-2</td><td>2</td><td>-21.3</td><td>3' GGCGAACTGGCTCCGGGGGTA 5'
    |:||| ||| ||||||
5' CTGCTAAACCACA-CCCCCA- 3'</td></tr>
<tr><td>H2-Ab1:::NM_207105</td><td>mcmv-miR-M23-2</td><td>1</td><td>-21</td><td>3' GGCGAACTGG--CTCCGGGGGTA 5'
         |||:| ||| ||||||
5' -----TGATCTGGAGTCCCCCA- 3'</td></tr>
<tr><td>Hdac7a:::NM_019572</td><td>mcmv-miR-M23-2</td><td>1</td><td>-21</td><td>3' GGCGAACTGGCTCCGGGGGTA 5'
    || || :|:| ||:|:||||
5' CCCCTA-GCTGTGGTCTCCAT 3'</td></tr>
<tr><td>Klf11:::NM_178357</td><td>mcmv-miR-M23-2</td><td>1</td><td>-21</td><td>3' GGCGAACTGGCTCCGGGGGTA 5'
         ||| || |||||||
5' -----TGAAAGA-GCCCCCA- 3'</td></tr>
<tr><td>Nfam1:::NM_028728</td><td>mcmv-miR-M23-2</td><td>2</td><td>-21</td><td>3' GGCGAACTGG--CTC--CGGGGGTA 5'
       :|||:| ||| |||:|||
5' ---TTTGGCACAGAGCTGCCTCCA- 3'</td></tr>
<tr><td>Il2ra:::NM_008367</td><td>mcmv-miR-M23-2</td><td>3</td><td>-20.9</td><td>3' GGCGAACTGGCTCCGGGGGTA 5'
          || ||||||||
5' ------GAAAT-GGCCCCCA- 3'</td></tr>
<tr><td>Olr1:::NM_138648</td><td>mcmv-miR-M23-2</td><td>2</td><td>-20.9</td><td>3' GGCGAACTGGCTCCGGGGGTA 5'
      || |:|: ||||:|||
5' --GCA-GGCT--GGCCTCCA- 3'</td></tr>
<tr><td>Podxl:::NM_013723</td><td>mcmv-miR-M23-2</td><td>1</td><td>-20.9</td><td>3' GGCGAACTGGCTCCGGGGGTA 5'
          ||| |||:|||||
5' ------GAC--AGGTCCCCA- 3'</td></tr>
<tr><td>Rhoh:::NM_001081105</td><td>mcmv-miR-M23-2</td><td>2</td><td>-20.9</td><td>3' GGCGAACTGGCTCCGGGGGTA 5'
    || || :|: |||||||
5' CC-CTGAGCTAT-GCCCCCA- 3'</td></tr>
<tr><td>Unc13d:::NM_001009573</td><td>mcmv-miR-M23-2</td><td>1</td><td>-20.8</td><td>3' GGCGAACTGGCTCCGGGGGTA 5'
    |:|| ||| ||||||
5' CTGCACCCCCGTC-CCCCCA- 3'</td></tr>
<tr><td>Hs1bp3:::NM_021429</td><td>mcmv-miR-M23-2</td><td>3</td><td>-20.7</td><td>3' GGCGAACTGGCTCCGGGGGTA 5'
    |: || |:: |||:|||||
5' CTCCTAGGTTAAGGTCCCCA- 3'</td></tr>
</table>

Supplementary Table 1. page 8

<table>
<tr><td>Lta:::NM_010735</td><td>mcmv-miR-M23-2</td><td>2</td><td>-20.7</td><td>3' GGCGAACT---GGCTCCGGGGGTA 5'
       |||: || |||||:||||
5' ----TTGGATTCC-AGGCCTCCAT 3'</td></tr>
<tr><td>Kirrel3:::NM_026324</td><td>mcmv-miR-M23-2</td><td>1</td><td>-20.6</td><td>3' GGCGAACTGGCTC-CGGGGGTA 5'
     || ||: | |||||||
5' --GCCTGGGAGTCCGCCCCCA- 3'</td></tr>
<tr><td>Klrk1:::NM_001083322</td><td>mcmv-miR-M23-2</td><td>2</td><td>-20.6</td><td>3' GGCGAACTGGCTCC----GGGGGTA 5'
        ||| :|||| ||||||
5' -----TGAATGAGGGATTCCCCCA- 3'</td></tr>
<tr><td>Irf4:::NM_013674</td><td>mcmv-miR-M23-2</td><td>2</td><td>-20.5</td><td>3' GGCGAACTGGCTCCGGGGGTA 5'
         | ||| |||||||
5' ------GTTCGA-GCCCCCA- 3'</td></tr>
<tr><td>Tlr13:::NM_205820</td><td>mcmv-miR-M23-2</td><td>1</td><td>-20.5</td><td>3' GGCGAACTGGCTCCGGGGGTA 5'
    :||| || |||::||||
5' -TGCT---CC-AGGTTCCCA- 3'</td></tr>
<tr><td>Cbfa2t3h:::NM_009824</td><td>mcmv-miR-M23-2</td><td>5</td><td>-20.4</td><td>3' GGCGAACTGGCTC-CGGGGGTA 5'
    :||| || || |||:|||
5' -TGCTCC-CCCAGAGCCTCCA- 3'</td></tr>
<tr><td>Cxcl16:::NM_023158</td><td>mcmv-miR-M23-2</td><td>8</td><td>-20.4</td><td>3' GGCGAACTGGCTCCGGGGGTA 5'
    ||: || |||:|:|||
5' -CGTATC-CCTAGGTCTCCA- 3'</td></tr>
<tr><td>Il1f10:::NM_153077</td><td>mcmv-miR-M23-2</td><td>1</td><td>-20.4</td><td>3' GGCGAACTGGC-----TC-CGGGGGTA 5'
         |:|| || |||||||
5' ------GGCCTCGTCTAGTGCCCCCA- 3'</td></tr>
<tr><td>Tbkbp1:::NM_198100</td><td>mcmv-miR-M23-2</td><td>2</td><td>-20.4</td><td>3' GGCGAACTGGCTCCGGGGGTA 5'
   || |||| | ||:|||||
5' CC-CTTG-C---GGTCCCCA- 3'</td></tr>
<tr><td>Kdr:::NM_010612</td><td>mcmv-miR-M23-2</td><td>1</td><td>-20.3</td><td>3' GGCGAACTGGCTCCGGGGGTA 5'
         |:|: |||||||
5' ------GGCTCC-GCCCCCA- 3'</td></tr>
<tr><td>Cebpe:::NM_207131</td><td>mcmv-miR-M23-2</td><td>1</td><td>-20.2</td><td>3' GGCGAACTGGCTCCGGGGGTA 5'
   || ||| |: |||:|||||
5' CC-CTT--CT-AGGTCCCCA- 3'</td></tr>
<tr><td>Il1r1:::NM_008362</td><td>mcmv-miR-M23-2</td><td>1</td><td>-20.2</td><td>3' GGCGAACTGGCTCCGGGGGTA 5'
    | ||| ||| |||||||
5' -CTCTT-ACCCCAGCCCCCA- 3'</td></tr>
<tr><td>Tnrc5:::NM_028065</td><td>mcmv-miR-M23-2</td><td>1</td><td>-20.2</td><td>3' GGCGAACTGGCTC--CGGGGGTA 5'
        ||||: |||||||
5' -----TGACTCCCACGCCCCCA- 3'</td></tr>
<tr><td>Il13:::NM_008355</td><td>mcmv-miR-M23-2</td><td>2</td><td>-20</td><td>3' GGCGAACTGGCTCCGGGGGTA 5'
     || ||:| ||||||
5' --GCCTGGCAACA-CCCCCA- 3'</td></tr>
<tr><td>Ank1:::NM_031158</td><td>mcmv-miR-M23-2</td><td>1</td><td>-19.9</td><td>3' GGCGAACTGGCTCCGGGGGTA 5'
   || || || ||||||||
5' CC-CTACCCCCCAGCCCCCAT 3'</td></tr>
<tr><td>Bcl3:::NM_033601</td><td>mcmv-miR-M23-2</td><td>1</td><td>-19.9</td><td>3' GGCGAACTGGCTC-CGGGGGTA 5'
         |||: |||||||
5' ------GACTCTCAGCCCCCA- 3'</td></tr>
<tr><td>Tnf:::NM_013693</td><td>mcmv-miR-M23-2</td><td>3</td><td>-19.9</td><td>3' GGCGAACTGGCTC--CGGGGGTA 5'
    || :|:||| |:|||||
5' -CGGA--GCTGAGCTGTCCCCA- 3'</td></tr>
<tr><td>Isg15:::NM_015783</td><td>mcmv-miR-M23-2</td><td>1</td><td>-19.8</td><td>3' GGCGAACTGGCTCCGGGGGTA 5'
               |||||||||
5' ------------GGCCCCCAT 3'</td></tr>
<tr><td>Prkca:::NM_011101</td><td>mcmv-miR-M23-2</td><td>1</td><td>-19.6</td><td>3' GGCGAACTGGCTCCGGGGGTA 5'
    :|| || |||||||
5' -TGCCT--CCCCAGCCCCCA- 3'</td></tr>
<tr><td>Ttc7:::NM_028639</td><td>mcmv-miR-M23-2</td><td>2</td><td>-19.6</td><td>3' GGCGAACTGGCTCCGGGGGTA 5'
    | ||||:|: ||||||
5' -C-CTTGGCTT---CCCCCA- 3'</td></tr>
<tr><td>Lif:::NM_001039537</td><td>mcmv-miR-M23-2</td><td>1</td><td>-19.5</td><td>3' GGCGAACTGGCTCC-GGGGGTA 5'
     | || :| || ||||||
5' --GGGTG-TCAGGGTCCCCCA- 3'</td></tr>
<tr><td>Mnx1:::NM_019944</td><td>mcmv-miR-M23-2</td><td>1</td><td>-19.5</td><td>3' GGCGAACTGGCTCCGGGGGTA 5'
     |||| || ||||||
5' --GCTTTCCCAT--CCCCCA- 3'</td></tr>
<tr><td>Nlrx1:::NM_178420</td><td>mcmv-miR-M23-2</td><td>1</td><td>-19.5</td><td>3' GGCGAACTGGCTCCGGGGGTA 5'
   |:|:   :|| ||||||
5' CTGTGCTGCCCCCACCCCCA- 3'</td></tr>
<tr><td>Slc7a2:::NM_001044740</td><td>mcmv-miR-M23-2</td><td>1</td><td>-19.5</td><td>3' GGCGAACTGGC---TCCGGGGGTA 5'
         |:| |||||:||:
5' ------GGCAGAATAGGCCTCCG- 3'</td></tr>
<tr><td>Cnih:::NM_009919</td><td>mcmv-miR-M23-2</td><td>1</td><td>-19.4</td><td>3' GGCGAACTGGCTCCGGGGGTA 5'
        ||||| ||||:||
5' -----TGACC---GCCCTCA- 3'</td></tr>
<tr><td>Ifnar1:::NM_010508</td><td>mcmv-miR-M23-2</td><td>1</td><td>-19.4</td><td>3' GGCGAACT-GGCTC----CGGGGGTA 5'
         || |:||| |:|||||
5' ------GATCTGAGCTCTGTCCCCA- 3'</td></tr>
<tr><td>Nod2:::NM_145857</td><td>mcmv-miR-M23-2</td><td>3</td><td>-19.4</td><td>3' GGCGAAC-TGGCTCCGGGGGTA 5'
   || | ||| ||||||||
5' CCACCCCCACCCCCGCCCCCAT 3'</td></tr>
<tr><td>Chrna4:::NM_015730</td><td>mcmv-miR-M23-2</td><td>2</td><td>-19.3</td><td>3' GGCGAACTGGCTCCGGGGGTA 5'
    ||| ||| |||:|||
5' -CGC---ACCAA-GCCTCCA- 3'</td></tr>
<tr><td>Spib:::NM_019866</td><td>mcmv-miR-M23-2</td><td>1</td><td>-19.2</td><td>3' GGC-GAACTGGCTCCGGGGGTA 5'
     | |||| | ||||||
5' --GACTTGGTCAA--CCCCCA- 3'</td></tr>
<tr><td>B4galt1:::NM_022305</td><td>mcmv-miR-M23-2</td><td>1</td><td>-19</td><td>3' GGCGAACTGGCTCCGGGGGTA 5'
           |||| |||:|||
5' --------CCGA-GCCTCCA- 3'</td></tr>
<tr><td>Clcf1:::NM_019952</td><td>mcmv-miR-M23-2</td><td>1</td><td>-19</td><td>3' GGCGAACTGGCTCC-GGGGGTA 5'
        ||||| ||||||
5' -----TGACCCTTAACCCCCA- 3'</td></tr>
<tr><td>P2rx7:::NM_011027</td><td>mcmv-miR-M23-2</td><td>2</td><td>-18.8</td><td>3' GGCGAACTG-GCTCC---GGGGGTA 5'
     || ||:| :| | ||||||
5' --GCATGGCATGTGTCCACCCCCA- 3'</td></tr>
<tr><td>Tshr:::NM_011648</td><td>mcmv-miR-M23-2</td><td>1</td><td>-18.8</td><td>3' GGCGAACTGGCTCCGGGGGTA 5'
     || ||||: :|||||
5' --GC---ACCGGCTTCCCCA- 3'</td></tr>
</table>

Supplementary Table 1. page 9

<table>
<tr><td>Bysl:::NM_016859</td><td>mcmv-miR-M23-2</td><td>1</td><td>-18.7</td><td>3' GGCGAACTGGCTCCGGGGGTA 5'
     ||| || |:|||||
5' --GCTCCTCCCT-GTCCCA- 3'</td></tr>
<tr><td>Tirap:::NM_054096</td><td>mcmv-miR-M23-2</td><td>3</td><td>-18.7</td><td>3' GGCGAACTGGCTCC---GGGGGTA 5'
          :|| || ||||||
5' -------GCCAGGGACTCCCCCA- 3'</td></tr>
<tr><td>Hsh2d:::NM_197944</td><td>mcmv-miR-M23-2</td><td>1</td><td>-18.6</td><td>3' GGCGAACTGGC--TCCGGGGGTA 5'
           || |||:||||||
5' --------CCTTTAGGTCCCCAT 3'</td></tr>
<tr><td>Nlrp3:::NM_145827</td><td>mcmv-miR-M23-2</td><td>1</td><td>-18.6</td><td>3' GGCGAACTGGCTCCGGGGGTA 5'
   |||||| | ||||||
5' CCGCTTCTC-----CCCCCA- 3'</td></tr>
<tr><td>Mink1:::NM_001045959</td><td>mcmv-miR-M23-2</td><td>1</td><td>-18.5</td><td>3' GGCGAACTGGCTCC---GGGGGTA 5'
    ||: |||| ||||||
5' -CGT--GACCACCCTTTCCCCCA- 3'</td></tr>
<tr><td>Bak1:::NM_007523</td><td>mcmv-miR-M23-2</td><td>3</td><td>-18.4</td><td>3' GGCGAACTGGCTCCGGGGGTA 5'
   |:|:   :| |||||||
5' CTGT---GCAAT-GCCCCCA- 3'</td></tr>
<tr><td>Cebpg:::NM_009884</td><td>mcmv-miR-M23-2</td><td>1</td><td>-18.4</td><td>3' GGCGAACTGGCTC--CGGGGGTA 5'
        ||:|| |||:|||
5' -----TGGCCTTCCTGCCTCCA- 3'</td></tr>
<tr><td>Sox6:::NM_001025559</td><td>mcmv-miR-M23-2</td><td>5</td><td>-18.4</td><td>3' GGCGAACTGGCTCCGGGGGTA 5'
    |||| || ||||||
5' -CGCT---CCCTTTCCCCCA- 3'</td></tr>
<tr><td>Vegfa:::NM_001025250</td><td>mcmv-miR-M23-2</td><td>1</td><td>-18.4</td><td>3' GGCGAACTGGCTCCGGGGGTA 5'
   |:|| || |:|||||
5' CTGCCCT-CCCTTGTCCCCA- 3'</td></tr>
<tr><td>Sla2:::NM_029983</td><td>mcmv-miR-M23-2</td><td>2</td><td>-18.3</td><td>3' GGCGAA--CTGGCTCC----GGGGGTA 5'
   |||| |||| |:||||
5' CCGCAGTAGACCCCACCTAACTCCCA- 3'</td></tr>
<tr><td>Bcl11b:::NM_001079883</td><td>mcmv-miR-M23-2</td><td>3</td><td>-18.2</td><td>3' GGCGAACTGG----CTC--CGGGGGTA 5'
         |||: || |||||||
5' ------GACTCTAGGAACAGCCCCCA- 3'</td></tr>
<tr><td>Cd300lf:::NM_145634</td><td>mcmv-miR-M23-2</td><td>1</td><td>-18.2</td><td>3' GGCGAACTGGCTCCGGGGGTA 5'
   |||| || ||||||
5' CCGCC---CCTC--CCCCCA- 3'</td></tr>
<tr><td>Tcfe2a:::NM_011548</td><td>mcmv-miR-M23-2</td><td>1</td><td>-18.2</td><td>3' GGCGAACTGGCTCCGGGGGTA 5'
         ||| |||:|:||||
5' ------GAC--AGGTCTCCAT 3'</td></tr>
<tr><td>Barx1:::NM_007526</td><td>mcmv-miR-M23-2</td><td>1</td><td>-18</td><td>3' GGCGAACTGGCTCCGGGGGTA 5'
           || ||||:|||
5' --------CCCCGGCCTCCA- 3'</td></tr>
<tr><td>Ccl21a:::NM_011335</td><td>mcmv-miR-M23-2</td><td>1</td><td>-18</td><td>3' GGCGAACT-GGCTCC----GGGGGTA 5'
         || || ||| ||||||
5' ------GAGCCCAGGAGATCCCCCA- 3'</td></tr>
<tr><td>Ccl21b:::NM_011124</td><td>mcmv-miR-M23-2</td><td>1</td><td>-18</td><td>3' GGCGAACT-GGCTCC----GGGGGTA 5'
         || || ||| ||||||
5' ------GAGCCCAGGAGATCCCCCA- 3'</td></tr>
<tr><td>Ccl21c:::NM_023052</td><td>mcmv-miR-M23-2</td><td>1</td><td>-18</td><td>3' GGCGAACT-GGCTCC----GGGGGTA 5'
         || || ||| ||||||
5' ------GAGCCCAGGAGATCCCCCA- 3'</td></tr>
<tr><td>Flvcr1:::NM_001081259</td><td>mcmv-miR-M23-2</td><td>1</td><td>-17.9</td><td>3' GGCGAACTGGCTCCGGGGGTA 5'
    :|| ||:| ||||||
5' -TGC---ACTGTCTCCCCCA- 3'</td></tr>
<tr><td>Sgpl1:::NM_009163</td><td>mcmv-miR-M23-2</td><td>1</td><td>-17.8</td><td>3' GGCGAACTGGCTCCGGGGGTA 5'
     ||| :| |||||::||
5' --GCT---TC-AGGCCTTCA- 3'</td></tr>
<tr><td>Ndrg1:::NM_008681</td><td>mcmv-miR-M23-2</td><td>2</td><td>-17.7</td><td>3' GGCGAA-CTGGCTCCGGGGGTA 5'
    ||:| || : ||||:||
5' -CGTTGGGAGTCA-GCCCTCA- 3'</td></tr>
<tr><td>Icam1:::NM_010493</td><td>mcmv-miR-M23-2</td><td>1</td><td>-17.4</td><td>3' GGCGAACTGGCTCCGGGGGTA 5'
         ||| |:|||||
5' ------GACATT-GTCCCCA- 3'</td></tr>
<tr><td>Kit:::NM_021099</td><td>mcmv-miR-M23-2</td><td>2</td><td>-17.4</td><td>3' GGCGAACTGGCTCCGGGGGTA 5'
    | |||| || ||||||
5' -C-CTTG-CCCT--CCCCCA- 3'</td></tr>
<tr><td>Skap1:::NM_001033186</td><td>mcmv-miR-M23-2</td><td>1</td><td>-17.4</td><td>3' GGCGAACT-GGCTCCGGGGGTA 5'
     || |:| |:|||||
5' --GCCCTCTCTGT-GTCCCCA- 3'</td></tr>
<tr><td>Tnfsf12:::NM_011614</td><td>mcmv-miR-M23-2</td><td>2</td><td>-17.4</td><td>3' GGCGAACTGGCTCCGGGGGTA 5'
   || | ||||: ||||||
5' CC-C-TGACT----CCCCCA- 3'</td></tr>
<tr><td>Pbx1:::NM_183355</td><td>mcmv-miR-M23-2</td><td>3</td><td>-17.3</td><td>3' GGCGAACTGGCTC-CGGGGGTA 5'
   || | || :| ||||:|||
5' CCTC---ACTAGGAGCCCTCAT 3'</td></tr>
<tr><td>Tgfbr1:::NM_009370</td><td>mcmv-miR-M23-2</td><td>2</td><td>-17.3</td><td>3' GGCGA--ACTGGCTCCGGGGGTA 5'
    :|:| ||||| ||:|||
5' -TGTTCCTGACCCCA-CCTCCA- 3'</td></tr>
<tr><td>Bank1:::NM_001033350</td><td>mcmv-miR-M23-2</td><td>1</td><td>-17.2</td><td>3' GGCGAACTGGCTCCGGGGGTA 5'
   |:| ||| ||:|:|||
5' CTGGAATACCT-GGTCTCCA- 3'</td></tr>
<tr><td>Vnn1:::NM_011704</td><td>mcmv-miR-M23-2</td><td>1</td><td>-17.2</td><td>3' GGCGAACTGGCTCCGGGGGTA 5'
           || |||:|:|||
5' --------CC-AGGTCTCCA- 3'</td></tr>
<tr><td>Procr:::NM_011171</td><td>mcmv-miR-M23-2</td><td>1</td><td>-17.1</td><td>3' GGCGAACTGGCTCCGGGGGTA 5'
    :|| || || ||||||
5' -TGCCAAACAGACTCCCCCA- 3'</td></tr>
<tr><td>Twsg1:::NM_023053</td><td>mcmv-miR-M23-2</td><td>1</td><td>-17.1</td><td>3' GGCGAACTGGC--TCCGGGGGTA 5'
     || || :||:|:|||
5' --GCAGAACAGCAGGGTCTCCA- 3'</td></tr>
<tr><td>Mapk1:::NM_011949</td><td>mcmv-miR-M23-2</td><td>1</td><td>-17.07</td><td>3' GGCGAACTGGCTCCGGGGGTA 5'
    :|| |||||||
5' -TGCATTTATTCTGCCCCCA- 3'</td></tr>
<tr><td>Il10:::NM_010548</td><td>mcmv-miR-M23-2</td><td>1</td><td>-16.9</td><td>3' GGCGAACTGGCTCC-GGGGGTA 5'
   || || ||:| ||||||
5' CC-CT--ACTGTCATCCCCCA- 3'</td></tr>
<tr><td>Sfxn1:::NM_027324</td><td>mcmv-miR-M23-2</td><td>2</td><td>-16.9</td><td>3' GGCGAACTGGCTCCGGGGGTA 5'
           || |||||||
5' --------CCAAAGCCCCCA- 3'</td></tr>
</table>

Supplementary Table 1. page 10

<table>
<tr><td>Il25:::NM_080729</td><td>mcmv-miR-M23-2</td><td>1</td><td>-16.8</td><td>3' GGCGAACTGGCTCCGGGGGTA 5'
            |:|| ||||||
5' --------CTGAATCCCCCA- 3'</td></tr>
<tr><td>Selp:::NM_011347</td><td>mcmv-miR-M23-2</td><td>3</td><td>-16.8</td><td>3' GGCGAACTGGCTCCGGGGGTA 5'
   |:|| |||: ||:|||
5' CTGCA-GACTTTC-CCTCCA- 3'</td></tr>
<tr><td>Syk:::NM_011518</td><td>mcmv-miR-M23-2</td><td>1</td><td>-16.8</td><td>3' GGCGA-ACTGGCTCCGGGGGTA 5'
     | ||||| :|||||
5' --GATGTGACCT---TCCCCA- 3'</td></tr>
<tr><td>5830411N06Rik:::NM_175533</td><td>mcmv-miR-M23-2</td><td>1</td><td>-16.7</td><td>3' GGCGAACTGGCTCCGGGGGTA 5'
   || | || :| |||:|:|||
5' CC-C-TG-TC-AGGTCTCCA- 3'</td></tr>
<tr><td>Pbx1:::NM_008783</td><td>mcmv-miR-M23-2</td><td>1</td><td>-16.7</td><td>3' GGCGAACTGGCTCC--GGGGGTA 5'
      ||| ||:| ||||||
5' ---CTTTACTGTTGTACCCCCA- 3'</td></tr>
<tr><td>Tap2:::NM_011530</td><td>mcmv-miR-M23-2</td><td>1</td><td>-16.7</td><td>3' GGCGAACTGGCTCCGGGGGTA 5'
               ||||:|||
5' ------------GGCCTCCA- 3'</td></tr>
<tr><td>Add1:::NM_001102444</td><td>mcmv-miR-M23-2</td><td>1</td><td>-16.6</td><td>3' GGCGAACTGGCTCCGGGGGTA 5'
         |:| |:||||||
5' ------GGCAA--GTCCCCAT 3'</td></tr>
<tr><td>Cxcl14:::NM_019568</td><td>mcmv-miR-M23-2</td><td>2</td><td>-16.6</td><td>3' GGCGAACTGGCTCCGGGGGTA 5'
     || || |:|||||
5' --GCAT--CCAAAGTCCCCA- 3'</td></tr>
<tr><td>Ercc2:::NM_007949</td><td>mcmv-miR-M23-2</td><td>1</td><td>-16.6</td><td>3' GGCGAACTGGCTCCGGGGGTA 5'
   |:|| ||| :|||||
5' CTGC----CCGCCTTCCCCA- 3'</td></tr>
<tr><td>Fcgr1:::NM_010186</td><td>mcmv-miR-M23-2</td><td>1</td><td>-16.6</td><td>3' GGCGAACTGG-CTCCGGGGGTA 5'
         | || || ||||||
5' ------GTCCAGATACCCCCA- 3'</td></tr>
<tr><td>Bmi1:::NM_007552</td><td>mcmv-miR-M23-2</td><td>1</td><td>-16.5</td><td>3' GGCGAACTGGCTCCGGGGGTA 5'
    :| ||||| ||:|||
5' -TGAATGACC----CCTCCA- 3'</td></tr>
<tr><td>Crkl:::NM_007764</td><td>mcmv-miR-M23-2</td><td>1</td><td>-16.5</td><td>3' GGCGAACTGG---CTCCGGGGGTA 5'
         |:|| || |:|:|||
5' ------GGCCTTTGATGTCTCCA- 3'</td></tr>
<tr><td>Irf2:::NM_008391</td><td>mcmv-miR-M23-2</td><td>1</td><td>-16.5</td><td>3' GGCGAACTGGCTCCGGGGGTA 5'
    ||| || ||||||
5' -CGC----CCCTTCCCCCA- 3'</td></tr>
<tr><td>Osm:::NM_001013365</td><td>mcmv-miR-M23-2</td><td>1</td><td>-16.5</td><td>3' GGC-GAACT--GGCTCCGGGGGTA 5'
     | ||||| |:| ||:|||
5' --GACTTGAAACTGTTTCCTCCA- 3'</td></tr>
<tr><td>Pik3cd:::NM_001029837</td><td>mcmv-miR-M23-2</td><td>1</td><td>-16.5</td><td>3' GGCGAACTGGCTC---CGGGGGTA 5'
            ||:| ||||:||
5' ---------CGGGAACGCCCTCA- 3'</td></tr>
<tr><td>Samhd1:::NM_018851</td><td>mcmv-miR-M23-2</td><td>1</td><td>-16.47</td><td>3' GGCGAACTGGCTCCGGGGGTA 5'
     ||| ||||||
5' --GCTGTAAGCACACCCCCA- 3'</td></tr>
<tr><td>Slamf1:::NM_013730</td><td>mcmv-miR-M23-2</td><td>1</td><td>-16.4</td><td>3' GGCGAACTGGCTCC---------GGGGGTA 5'
             |||| ||||||
5' ----------GAGGAAGATACTCCCCCCA- 3'</td></tr>
<tr><td>Egr1:::NM_007913</td><td>mcmv-miR-M23-2</td><td>1</td><td>-16.1</td><td>3' GGCGAACTGGCTCCGGGGGTA 5'
     |||| ||:|:|||
5' --GCTTTC----GGTCTCCA- 3'</td></tr>
<tr><td>Trp53:::NM_011640</td><td>mcmv-miR-M23-2</td><td>1</td><td>-16.1</td><td>3' GGCG-AACTGGCTCCGGGGGTA 5'
   |:|| || |:| ||||||
5' CTGCATTTATCCAC-CCCCCA- 3'</td></tr>
<tr><td>Cd28:::NM_007642</td><td>mcmv-miR-M23-2</td><td>1</td><td>-16</td><td>3' GGCGAACTGGCTCCGGGGGTA 5'
     || || ||||||
5' --GCATTTCCCCTCCCCCCA- 3'</td></tr>
<tr><td>Wnt3a:::NM_009522</td><td>mcmv-miR-M23-2</td><td>1</td><td>-15.9</td><td>3' GGCGAACT-GGC-TCCGGGGGTA 5'
         || ::| || ||||||
5' ------GATTTGCAGTCCCCCA- 3'</td></tr>
<tr><td>Il15:::NM_008357</td><td>mcmv-miR-M23-2</td><td>1</td><td>-15.8</td><td>3' GGCGAACTGGCTCC--GGGGGTA 5'
   |:|:| |: ||| ||:|||
5' CTGTT--ATTAAGGTACCTCCA- 3'</td></tr>
<tr><td>Oasl2:::NM_011854</td><td>mcmv-miR-M23-2</td><td>1</td><td>-15.8</td><td>3' GGCGAACTGGCTCC---GGGGGTA 5'
         |||:| :|||||
5' ------GACTGTCAGATTCCCCA- 3'</td></tr>
<tr><td>Runx1:::NM_009821</td><td>mcmv-miR-M23-2</td><td>2</td><td>-15.76</td><td>3' GGCGAACTGGCTCC---GGGGGTA 5'
      ||||| ||||||
5' ---CTTGAAATAACTTTCCCCCA- 3'</td></tr>
<tr><td>Azgp1:::NM_013478</td><td>mcmv-miR-M23-2</td><td>1</td><td>-15.7</td><td>3' GGCGAACTGGCTCCGGGGGTA 5'
                |||||||
5' -------------GCCCCCA- 3'</td></tr>
<tr><td>Ccl1:::NM_011329</td><td>mcmv-miR-M23-2</td><td>1</td><td>-15.6</td><td>3' GGCGAACTGGCTCCGGGGGTA 5'
     |:|| |: || ||:|||
5' --GTTTCCCTCAGTCCTCCA- 3'</td></tr>
<tr><td>Cd19:::NM_009844</td><td>mcmv-miR-M23-2</td><td>1</td><td>-15.6</td><td>3' GGCGAACTGGCTCC-GGGGGTA 5'
         |:|| || |||:||
5' ------GGCC-AGTACCCTCA- 3'</td></tr>
<tr><td>Apc:::NM_007462</td><td>mcmv-miR-M23-2</td><td>1</td><td>-15.5</td><td>3' GGCGAACTGGCTCCGGGGGTA 5'
        ||| |:|||||
5' -----TGAAGTCTGTCCCCA- 3'</td></tr>
<tr><td>Hoxa9:::NM_010456</td><td>mcmv-miR-M23-2</td><td>1</td><td>-15.5</td><td>3' GGCGAACTGGCTCCGGGGGTA 5'
   || | ||| |||:|||
5' CC-CCAAACCCA-GCCTCCA- 3'</td></tr>
<tr><td>Inpp5d:::NM_010566</td><td>mcmv-miR-M23-2</td><td>1</td><td>-15.5</td><td>3' GGCGAACTGGCTCC-----GGGGGTA 5'
          ||:|||| :|:|||
5' -------ACTGAGGAAGAGTCTCCA- 3'</td></tr>
<tr><td>Tcfe3:::NM_172472</td><td>mcmv-miR-M23-2</td><td>1</td><td>-15.5</td><td>3' GGCGAACT-GGCTCCGGGGGTA 5'
         || || |:|||||
5' ------GAGCCA--GTCCCCA- 3'</td></tr>
<tr><td>Hells:::NM_008234</td><td>mcmv-miR-M23-2</td><td>1</td><td>-15.4</td><td>3' GGCGAACTGGCTCCGGGGGTA 5'
     |:|| | :| ||||||
5' --GTTT-AGTGTCTCCCCCA- 3'</td></tr>
<tr><td>Lax1:::NM_172842</td><td>mcmv-miR-M23-2</td><td>1</td><td>-15.4</td><td>3' GGCGAACTGGCTCCGGGGGTA 5'
   || |||| | |||:||
5' CC-CTTG-CAATCTCCCTCA- 3'</td></tr>
</table>

Supplementary Table 1. page 11

<table>
<tr><td>Dbh:::NM_138942</td><td>mcmv-miR-M23-2</td><td>1</td><td>-15.3</td><td>3' GGCGAACTGGCTCCGGGGGTA 5'
           ||| |||:|||
5' -------ACCTT-GCCTCCA- 3'</td></tr>
<tr><td>Pml:::NM_008884</td><td>mcmv-miR-M23-2</td><td>3</td><td>-15.2</td><td>3' GGCGAACTGGCTCCGGGGGTA 5'
      ||| || ||:|||
5' --GCTAATCCCCACCCTCCA- 3'</td></tr>
<tr><td>Rbpj:::NM_001080927</td><td>mcmv-miR-M23-2</td><td>1</td><td>-15.2</td><td>3' GGCGAACTGGCTCCGGGGGTA 5'
           |: ||| |||:||
5' -------ATGGAGCCCCTCA- 3'</td></tr>
<tr><td>Il4ra:::NM_001008700</td><td>mcmv-miR-M23-2</td><td>1</td><td>-15.1</td><td>3' GGCGAACTGGCTCCGGGGGTA 5'
       ||| ||:| | ||:|||
5' ---CTTCACTG-GTCCTCCA- 3'</td></tr>
<tr><td>Pik3r1:::NM_001024955</td><td>mcmv-miR-M23-2</td><td>1</td><td>-15.1</td><td>3' GGCGAACTGGCTCCGGGGGTA 5'
      |||| :| ||:|||
5' --GCTTTGCAA---CCTCCA- 3'</td></tr>
<tr><td>Rag2:::NM_009020</td><td>mcmv-miR-M23-2</td><td>1</td><td>-15.1</td><td>3' GGCGAACTGGCTCC--GGGGGTA 5'
      |:   :||| :|||||
5' --GT---GCCGTACTCTCCCCA- 3'</td></tr>
<tr><td>Tollip:::NM_023764</td><td>mcmv-miR-M23-2</td><td>1</td><td>-15.1</td><td>3' GGCGAACTGGCTCCGGGGGTA 5'
    |:|| || |:||:||
5' CTGCATCTCCT--GTCCTCA- 3'</td></tr>
<tr><td>Cxcr4:::NM_009911</td><td>mcmv-miR-M23-2</td><td>1</td><td>-15</td><td>3' GGCGAACTGGCTCCGGGGGTA 5'
    || | ||| ||||||
5' CC-CCC-ACCCCCACCCCCA- 3'</td></tr>
<tr><td>Fech:::NM_007998</td><td>mcmv-miR-M23-2</td><td>1</td><td>-14.9</td><td>3' GGCGAACTG-GCTCCGGGGGTA 5'
          ||: :| |||:|||
5' ------GATGTGCAGCCTCCA- 3'</td></tr>
<tr><td>Fcamr:::NM_144960</td><td>mcmv-miR-M23-2</td><td>1</td><td>-14.8</td><td>3' GGCGAACTGGCTCCGGGGGTA 5'
    || ||| || ||:|||
5' CCTCTTCTCCTTTCCCTCCA- 3'</td></tr>
<tr><td>H2-Ob:::NM_010389</td><td>mcmv-miR-M23-2</td><td>1</td><td>-14.8</td><td>3' GGCGAACTGGCTCCGGGGGTA 5'
           :|| ||||||
5' -------GCCTCT-CCCCCA- 3'</td></tr>
<tr><td>Elf4:::NM_019680</td><td>mcmv-miR-M23-2</td><td>1</td><td>-14.7</td><td>3' GGCGA----ACTGGC-TCCGGGGGTA 5'
      ||| |||:| || |:|:||
5' --GCTCTCCTGATCTTAGTCTCTCA- 3'</td></tr>
<tr><td>Rsad2:::NM_021384</td><td>mcmv-miR-M23-2</td><td>1</td><td>-14.7</td><td>3' GGCGAAC-TGGCTCCGGGGGTA 5'
         || ||| ||||||
5' -----TGTACC----CCCCCA- 3'</td></tr>
<tr><td>C3ar1:::NM_009779</td><td>mcmv-miR-M23-2</td><td>1</td><td>-14.6</td><td>3' GGCGAACTGGCTCCGGGGGTA 5'
    || | || ||||||
5' CCACCAT-CCCCA-CCCCCA- 3'</td></tr>
<tr><td>Pax1:::NM_008780</td><td>mcmv-miR-M23-2</td><td>1</td><td>-14.5</td><td>3' GGCGAACTGGCTCCGGGGGTA 5'
           ||| ||||||
5' -------ACCCCTCCCCCCA- 3'</td></tr>
<tr><td>Psen2:::NM_011183</td><td>mcmv-miR-M23-2</td><td>1</td><td>-14.5</td><td>3' GGCGA-ACTGGCTCCGGGGGTA 5'
      |: ||| |:|||||
5' --GTCATGAAT---GTCCCCA- 3'</td></tr>
<tr><td>Hfe:::NM_010424</td><td>mcmv-miR-M23-2</td><td>1</td><td>-14.4</td><td>3' GGCGAAC-TGGCTCCGGGGGTA 5'
      |:| ||| ||:|||
5' --GTTGCAACCTCTCCCTCCA- 3'</td></tr>
<tr><td>Atp7a:::NM_009726</td><td>mcmv-miR-M23-2</td><td>1</td><td>-14.3</td><td>3' GGCGAAC-TGGC-TCCGGGGGTA 5'
          | || || ||||||
5' ------GAACATAAGTCCCCCA- 3'</td></tr>
<tr><td>Polm:::NM_017401</td><td>mcmv-miR-M23-2</td><td>1</td><td>-14.3</td><td>3' GGCGAACTGGCTCCGGGGGTA 5'
      | :|| ||||||
5' --GATAAGCCTT--CCCCCA- 3'</td></tr>
<tr><td>Polr3h:::NM_030229</td><td>mcmv-miR-M23-2</td><td>1</td><td>-14.3</td><td>3' GGCGAACTGGCTCCGGGGGTA 5'
      |:|| |:|| ||:|||
5' --GTTT--CTGAT-CCTCCA- 3'</td></tr>
<tr><td>Rb1:::NM_009029</td><td>mcmv-miR-M23-2</td><td>1</td><td>-14.2</td><td>3' GGCGAACTGGCTCC-GGGGGTA 5'
      || || ||||||
5' --GCACTCCCTTCACCCCCCA- 3'</td></tr>
<tr><td>Serping1:::NM_009776</td><td>mcmv-miR-M23-2</td><td>1</td><td>-14.2</td><td>3' GGCGAACTGGCTCCGGGGGTA 5'
           ||| ||||||
5' -------ACCAA--CCCCCA- 3'</td></tr>
<tr><td>Itga6:::NM_008397</td><td>mcmv-miR-M23-2</td><td>1</td><td>-14.1</td><td>3' GGCGAACTGGCTCCGGGGGTA 5'
          ||: ||||||
5' ------GATGCTCTCCCCCA- 3'</td></tr>
<tr><td>Dlg1:::NM_007862</td><td>mcmv-miR-M23-2</td><td>1</td><td>-14.01</td><td>3' GGCGAACTGGCTC-------CGGGGGTA 5'
          |:|| || ||:|:||
5' ------GGCC-AGTGTATTAGCTCTCA- 3'</td></tr>
<tr><td>Ccl9:::NM_011338</td><td>mcmv-miR-M23-2</td><td>1</td><td>-13.8</td><td>3' GGCGAACTGGCTCCGGGGGTA 5'
       :||| | :|||||
5' ---TTTGGTCT---TCCCCA- 3'</td></tr>
<tr><td>Ifit2:::NM_008332</td><td>mcmv-miR-M23-2</td><td>1</td><td>-13.8</td><td>3' GGCGAACTGG--CTC-CGGGGGTA 5'
          || || ||||:||
5' ------GAAAAAGAAAGCCCTCA- 3'</td></tr>
<tr><td>Pglyrp1:::NM_009402</td><td>mcmv-miR-M23-2</td><td>1</td><td>-13.8</td><td>3' GGCGAACT--GGCTCCGGGGGTA 5'
          || || ||||||
5' ------GAATCCCCCCCCCCCA- 3'</td></tr>
<tr><td>Tnfsf8:::NM_009403</td><td>mcmv-miR-M23-2</td><td>1</td><td>-13.8</td><td>3' GGCGAACTGGCTCC-GGGGGTA 5'
      |:|| | ||||||
5' --GTTTTTAAGTATTCCCCCA- 3'</td></tr>
<tr><td>Pknox1:::NM_016670</td><td>mcmv-miR-M23-2</td><td>1</td><td>-13.41</td><td>3' GGCGAACTGGCTCCGGGGGTA 5'
      || ||||||
5' --GCAAAATAAAA-CCCCCA- 3'</td></tr>
<tr><td>Rag1:::NM_009019</td><td>mcmv-miR-M23-2</td><td>2</td><td>-13.4</td><td>3' GGCGAACTGGCTCCGGGGGTA 5'
            || ||||||
5' --------CCCACTCCCCCA- 3'</td></tr>
<tr><td>Crcp:::NM_007761</td><td>mcmv-miR-M23-2</td><td>1</td><td>-13.3</td><td>3' GGCGAACTGGCTC-CGGGGGTA 5'
         || | ||:|:||
5' -----TGGTCACCAGCTCTCA- 3'</td></tr>
<tr><td>Ciapin1:::NM_134141</td><td>mcmv-miR-M23-2</td><td>1</td><td>-13.22</td><td>3' GGCGAACTGGCTCC-GGGGGTA 5'
          || ||||||
5' ------GAAGTCACTCCCCCA- 3'</td></tr>
<tr><td>Clec4e:::NM_019948</td><td>mcmv-miR-M23-2</td><td>1</td><td>-13</td><td>3' GGCGAACTGGCTCCGGGGGTA 5'
         |||| ||:|||
5' -----TGACATC--CCTCCA- 3'</td></tr>
</table>

Supplementary Table 1. page 12

<table>
<tr><td>Cr2:::NM_007758</td><td>mcmv-miR-M23-2</td><td>1</td><td>-13</td><td>3' GGCGAACTGGCTCCGGGGGTA 5'
                ||||:||
5' -------------GCCCTCA- 3'</td></tr>
<tr><td>Sod2:::NM_013671</td><td>mcmv-miR-M23-2</td><td>1</td><td>-12.3</td><td>3' GGCGAACTGGCTCCGGGGGTA 5'
   || || ||| :|||||
5' CC-CT--ACC----TCCCCA- 3'</td></tr>
<tr><td>Myd88:::NM_010851</td><td>mcmv-miR-M23-2</td><td>1</td><td>-12.1</td><td>3' GGCGAAC-TGGCTCCGGGGGTA 5'
     |: ||| ||:|||
5' --GTGTAAACCT---CCTCCA- 3'</td></tr>
<tr><td>Ccnd3:::NM_001081635</td><td>mcmv-miR-M23-2</td><td>1</td><td>-11.5</td><td>3' GGCGAAC--TGGCTCCGGGGGTA 5'
   || || ||| ||:|||
5' CCTCTCCTCACC----CCTCCA- 3'</td></tr>
<tr><td>Daf2:::NM_007827</td><td>mcmv-miR-M23-2</td><td>1</td><td>-11.4</td><td>3' GGCGAACTGGCTCCGGGGGTA 5'
         || |:|:|||
5' ------GAATCCAGTCTCCA- 3'</td></tr>
<tr><td>Cxcl12:::NM_001012477</td><td>mcmv-miR-M23-2</td><td>1</td><td>-10.4</td><td>3' GGCGAACTGGCTCCGGGGGTA 5'
   || | || |||:||
5' CC-CAAT-CCACTACCCTCA- 3'</td></tr>
<tr><td>Hoxb3:::NM_001079869</td><td>mcmv-miR-M23-2</td><td>1</td><td>-10.4</td><td>3' GGCGAACTGGCTCCGGGGGTA 5'
           || ||:|||
5' --------CCACTCCCTCCA- 3'</td></tr>
</table>

Supplementary Table 1. page 13

Predicted targets of both miR-m21-1 and miR-M23-2 ranked by minimal free energy (MFE) of binding

<table>
<tr><th>Gene symbol:::RefSeq</th><th>miRNA</th><th># binding sites</th><th>MFE (Kcal/mol)</th><th>Binding site</th><th>miRNA</th><th># binding sites</th><th>MFE (Kcal/mol)</th><th>Binding site</th></tr>
<tr><td>Ccl22:::NM_009137</td><td>mcmv-miR-M23-2</td><td>2</td><td>-27.5</td><td>3' GGCGAACTGGCTCCGGGGGTA 5'
|:| |:|| |||:|||||
5' --GTTAGGCCAAGGTCCCCA- 3'</td><td>mcmv-miR-m21-1</td><td>1</td><td>-12.2</td><td>3' GCCGAACTTGCACAGGGGATA 5'
||||||:
5' -------------TCCCCTG- 3'</td></tr>
<tr><td>Icosl:::NM_015790</td><td>mcmv-miR-M23-2</td><td>2</td><td>-26.4</td><td>3' GGCGAACT-GG-C-TCCGGGGGTA 5'
||| || | |||||||||
5' --GCTGATTCCAGCAGGCCCCCA- 3'</td><td>mcmv-miR-m21-1</td><td>1</td><td>-10.9</td><td>3' GCCGAACTTGCACAGGGGATA 5'
|| :|| |:||||:
5' -GGA---GACT--TTCCCTG- 3'</td></tr>
<tr><td>Tal1:::NM_011527</td><td>mcmv-miR-M23-2</td><td>1</td><td>-25.4</td><td>3' GGCGAAC-TGGCT-CCGGGGGTA 5'
| ||:|| ||||||||
5' ------GTACTGAGGGCCCCCA- 3'</td><td>mcmv-miR-m21-1</td><td>3</td><td>-14.6</td><td>3' GCCGAACTTGC--ACAGGGGATA 5'
||| ||||:|||||
5' -----TGATTCTTTGTCTCCTAT 3'</td></tr>
<tr><td>Tlx1:::NM_021901</td><td>mcmv-miR-M23-2</td><td>1</td><td>-24</td><td>3' GGCGAACT-GGC--TCCGGGGGTA 5'
||||: |:| ||||||:||
5' ---CTTGGTCTGTCAGGCCCTCA- 3'</td><td>mcmv-miR-m21-1</td><td>1</td><td>-11.7</td><td>3' GCCGAACTTGCACAGGGGATA 5'
||||||:
5' -------------TCCCCTG- 3'</td></tr>
<tr><td>March8:::NM_027920</td><td>mcmv-miR-M23-2</td><td>2</td><td>-23.9</td><td>3' GGCGAACTGGCT-CCGGGGGTA 5'
|||: || ||:|||||
5' -----TGATGGAGGGTCCCCA- 3'</td><td>mcmv-miR-m21-1</td><td>1</td><td>-17.8</td><td>3' GCCGAACTTGCACAGGGGATA 5'
:| ||||||||:|
5' --------GC-TGTCCCCTGT 3'</td></tr>
<tr><td>Tgfbr2:::NM_009371</td><td>mcmv-miR-M23-2</td><td>6</td><td>-23.3</td><td>3' GGCGAACTGGCTCCGGGGGTA 5'
||| |:|| |||:|||
5' --GCTGGGCCAA-GCCTCCA- 3'</td><td>mcmv-miR-m21-1</td><td>1</td><td>-13.2</td><td>3' GCCGAACTTGCACAGGGGATA 5'
|||:||||:
5' -----------TGTTCCCTG- 3'</td></tr>
<tr><td>Spn:::NM_009259</td><td>mcmv-miR-M23-2</td><td>1</td><td>-23.1</td><td>3' GGCGAACTGGCTCCGGGGGTA 5'
|: || |:| |||||||
5' --GTGTGTCTGCAGCCCCCA- 3'</td><td>mcmv-miR-m21-1</td><td>1</td><td>-18.3</td><td>3' GCCGAACTTGCAC--AGGGGATA 5'
||| |||| || ||||:|:|
5' -GGC-TGAA-GTCTTTCCCTTGT 3'</td></tr>
<tr><td>H2-Eb1:::NM_010382</td><td>mcmv-miR-M23-2</td><td>1</td><td>-22.8</td><td>3' GGCGAACTGGCTCC--GGGGGTA 5'
|||||:|| ||:|||
5' --GCTTGGCCCACATCCCTCCA- 3'</td><td>mcmv-miR-m21-1</td><td>2</td><td>-22.5</td><td>3' GCCGAA-CTTGCACAGGGGATA 5'
||| |:|| |||||||
5' -GGCGGGGGACCCATCCCCTA- 3'</td></tr>
<tr><td>Epas1:::NM_010137</td><td>mcmv-miR-M23-2</td><td>2</td><td>-22.7</td><td>3' GGCGAACTGGCTC---CGGGGGTA 5'
|| || |:|: ||||||||
5' --GC-TGTCTGGTAATGCCCCCAT 3'</td><td>mcmv-miR-m21-1</td><td>4</td><td>-11.1</td><td>3' GCCGAACTTGCACAGGGGATA 5'
||||||
5' --------------CCCCTA- 3'</td></tr>
<tr><td>H2-Q8:::NM_023124</td><td>mcmv-miR-M23-2</td><td>1</td><td>-22.4</td><td>3' GGCGAACTGGCTCCGGGGGTA 5'
| || |:|:|: ||||||
5' -CACTAGGCTGGTTCCCCCA- 3'</td><td>mcmv-miR-m21-1</td><td>1</td><td>-14.1</td><td>3' GCCGAACTTGCACAGGGGATA 5'
|| ||||||:|
5' ----------GT-TCCCCTGT 3'</td></tr>
<tr><td>Smad3:::NM_016769</td><td>mcmv-miR-M23-2</td><td>2</td><td>-22.3</td><td>3' GGCGAAC-TGGCTCCGGGGGTA 5'
|| || ||||| ||||||
5' CCACTGACACCGA--CCCCCA- 3'</td><td>mcmv-miR-m21-1</td><td>2</td><td>-22.3</td><td>5'
|||| |||| |||||:||:|
5' -GGCTCTTTGAAA-TGTCCTCTGT</td></tr>
<tr><td>Zbtb16:::NM_001033324</td><td>mcmv-miR-M23-2</td><td>3</td><td>-22.3</td><td>3' GGCGAACTGGCTCCGGGGGTA 5'
|:|| ||||||||
5' ------GGCCAC-GCCCCCAT 3'</td><td>mcmv-miR-m21-1</td><td>1</td><td>-16</td><td>3' GC-CGAACTTGCACAGGGGATA 5'
| |||| :| ||||||||
5' -GAGCTTT--TGAATCCCCTAT 3'</td></tr>
<tr><td>Cblb:::NM_001033238</td><td>mcmv-miR-M23-2</td><td>3</td><td>-22.3</td><td>3' GGCGAACTGGCTC--CGGGGGTA 5'
|:||| |:| ||||||||
5' CTGCT---CTGTGTTGCCCCCAT 3'</td><td>mcmv-miR-m21-1</td><td>1</td><td>-18.1</td><td>3' GCCGAACTTGCACAGGGGATA 5'
|||| :|| ||||||
5' -GGCTAAT-TGTAACCCCTA- 3'</td></tr>
<tr><td>Smad5:::NM_008541</td><td>mcmv-miR-M23-2</td><td>1</td><td>-22.2</td><td>3' GGCGAACTGGCTCCGGGGGTA 5'
:||||| || ||||||
5' -TGCTTG-CCCTT-CCCCCA- 3'</td><td>mcmv-miR-m21-1</td><td>1</td><td>-11.9</td><td>3' GCCGAACTTGCACAGGGGATA 5'
||||||:
5' -------------TCCCCTG- 3'</td></tr>
<tr><td>Rorc:::NM_011281</td><td>mcmv-miR-M23-2</td><td>2</td><td>-21.8</td><td>3' GGCGAACTGGCTCCGGGGGTA 5'
|:| ||:| |||||||
5' CTGG-TGGCAA--GCCCCCA- 3'</td><td>mcmv-miR-m21-1</td><td>2</td><td>-19.1</td><td>3' GCCGAACTTGCACAGGGGATA 5'
||| |:| |||||:||:|
5' --GCTCCAGC-TGTCCTCTGT 3'</td></tr>
<tr><td>Cplx2:::NM_009946</td><td>mcmv-miR-M23-2</td><td>5</td><td>-21.6</td><td>3' GGCGAACTGGCTCCGGGGGTA 5'
||| :|| || ||||||
5' --GCT--GCCCAGACCCCCA- 3'</td><td>mcmv-miR-m21-1</td><td>3</td><td>-20.1</td><td>3' GCCGAACTTGCACAGGGGATA 5'
|||| ||||||||:
5' CGGCCAA----TGTCCCCTG- 3'</td></tr>
<tr><td>Blr1:::NM_007551</td><td>mcmv-miR-M23-2</td><td>1</td><td>-21.4</td><td>3' GGCGAACTGGCTC-CGGGGGTA 5'
|:|| || |:|||||
5' ------GGCC-AGTGTCCCCA- 3'</td><td>mcmv-miR-m21-1</td><td>1</td><td>-28</td><td>5'
| |||||::| ||||||||
5' -GAGCTTGGGCCAGTGTCCCC---</td></tr>
<tr><td>Traf6:::NM_009424</td><td>mcmv-miR-M23-2</td><td>3</td><td>-21.4</td><td>3' GGCGAACTGGCTCCGGGGGTA 5'
|| ||| |:| |||:|||
5' CC-CTTAGCTGT-GCCTCCA- 3'</td><td>mcmv-miR-m21-1</td><td>3</td><td>-21.9</td><td>3' GCCGAACTTGCACAGGGGATA 5'
|| || ||||||||:|
5' -GGAACCTACATGTCCCCTGT 3'</td></tr>
<tr><td>Cx3cr1:::NM_009987</td><td>mcmv-miR-M23-2</td><td>2</td><td>-21.3</td><td>3' GGCGAACTGGCTCC-----GGGGGTA 5'
|||:||| ||||||
5' ------GACTGAGTCATATCCCCCA- 3'</td><td>mcmv-miR-m21-1</td><td>1</td><td>-17.6</td><td>3' GCCGAACTTGCACAGGGGATA 5'
||||| ||||||:
5' --GCTTGCTTTC-TCCCCTG- 3'</td></tr>
<tr><td>Ihh:::NM_010544</td><td>mcmv-miR-M23-2</td><td>2</td><td>-21.3</td><td>3' GGCGAACTGGCTCCGGGGGTA 5'
|:||| ||| ||||||
5' CTGCTAAACCACA-CCCCCA- 3'</td><td>mcmv-miR-m21-1</td><td>1</td><td>-18.4</td><td>3' GCCGAACTTGCAC-AGGGGATA 5'
|||| |:| ||||||:
5' -GGCTA-AGCTCCTTCCCCTG- 3'</td></tr>
<tr><td>Nfam1:::NM_028728</td><td>mcmv-miR-M23-2</td><td>2</td><td>-21</td><td>3' GGCGAACTGG--CTC--CGGGGGTA 5'
:|||:| ||| |||:|||
5' ---TTTGGCACAGAGCTGCCTCCA- 3'</td><td>mcmv-miR-m21-1</td><td>2</td><td>-13.4</td><td>3' GCCGAACTTGCACAGGGGATA 5'
|| ||||||:
5' ---------CGCCTCCCCTG- 3'</td></tr>
<tr><td>Podxl:::NM_013723</td><td>mcmv-miR-M23-2</td><td>1</td><td>-20.9</td><td>3' GGCGAACTGGCTCCGGGGGTA 5'
||| |||:|||||
5' ------GAC--AGGTCCCCA- 3'</td><td>mcmv-miR-m21-1</td><td>3</td><td>-21.6</td><td>3' GCCGAACT-TGCACAGGGGATA 5'
||| || | ||||||:||:
5' -GGCA-GACAGGTGTCCTCTG- 3'</td></tr>
<tr><td>Rhoh:::NM_001081105</td><td>mcmv-miR-M23-2</td><td>2</td><td>-20.9</td><td>3' GGCGAACTGGCTCCGGGGGTA 5'
|| || :|: |||||||
5' CC-CTGAGCTAT-GCCCCCA- 3'</td><td>mcmv-miR-m21-1</td><td>1</td><td>-11.44</td><td>3' GCCGAACTTGCACAGGGGATA 5'
||| |||:||:|
5' --GCTGTTTTC--TCCTCTGT 3'</td></tr>
<tr><td>Il2ra:::NM_008367</td><td>mcmv-miR-M23-2</td><td>3</td><td>-20.9</td><td>3' GGCGAACTGGCTCCGGGGGTA 5'
|| ||||||||
5' ------GAAAT-GGCCCCCA- 3'</td><td>mcmv-miR-m21-1</td><td>1</td><td>-12</td><td>3' GCCGAACTTGCACAGGGGATA 5'
|||||||
5' -------------TCCCCTA- 3'</td></tr>
<tr><td>Olr1:::NM_138648</td><td>mcmv-miR-M23-2</td><td>2</td><td>-20.9</td><td>3' GGCGAACTGGCTCCGGGGGTA 5'
|| |:|: ||||:|||
5' --GCA-GGCT--GGCCTCCA- 3'</td><td>mcmv-miR-m21-1</td><td>3</td><td>-25.1</td><td>3' GCCGAACTT--GCACAGGGGATA 5'
| ||||| ||||||||||:
5' -GAATTGAATTCGTGTCCCCTG- 3'</td></tr>
<tr><td>Hs1bp3:::NM_021429</td><td>mcmv-miR-M23-2</td><td>3</td><td>-20.7</td><td>3' GGCGAACTGGCTCCGGGGGTA 5'
|: || |:: |||:|||||
5' CTCCTAGGTTAAGGTCCCCA- 3'</td><td>mcmv-miR-m21-1</td><td>1</td><td>-20.5</td><td>3' GCCGAACTTGCACAGGGGATA 5'
|||||||||:|
5' ----------GTGTCCCCTGT 3'</td></tr>
<tr><td>Klrk1:::NM_001083322</td><td>mcmv-miR-M23-2</td><td>2</td><td>-20.6</td><td>3' GGCGAACTGGCTCC----GGGGGTA 5'
||| :|||| ||||||
5' -----TGAATGAGGGATTCCCCCA- 3'</td><td>mcmv-miR-m21-1</td><td>1</td><td>-12.2</td><td>3' GCCGAACTTGCACAGGGGATA 5'
|||||||
5' -------------TCCCCTA- 3'</td></tr>
<tr><td>Irf4:::NM_013674</td><td>mcmv-miR-M23-2</td><td>2</td><td>-20.5</td><td>3' GGCGAACTGGCTCCGGGGGTA 5'
| ||| |||||||
5' ------GTTCGA-GCCCCCA- 3'</td><td>mcmv-miR-m21-1</td><td>1</td><td>-11.2</td><td>3' GCCGAACTTGCACAGGGGATA 5'
|||| | |:|||||
5' ---CTTGCCC---TTCCCTA- 3'</td></tr>
<tr><td>Cxcl16:::NM_023158</td><td>mcmv-miR-M23-2</td><td>8</td><td>-20.4</td><td>3' GGCGAACTGGCTCCGGGGGTA 5'
||: || |||:|:|||
5' -CGTATC-CCTAGGTCTCCA- 3'</td><td>mcmv-miR-m21-1</td><td>10</td><td>-14.3</td><td>3' GCC-GAACTTGCACAGGGGATA 5'
|| || |:| |:||||||
5' -GGTCTCCAGCC--TTCCCTAT 3'</td></tr>
<tr><td>Tbkbp1:::NM_198100</td><td>mcmv-miR-M23-2</td><td>2</td><td>-20.4</td><td>3' GGCGAACTGGCTCCGGGGGTA 5'
|| |||| | ||:|||||
5' CC-CTTG-C---GGTCCCCA- 3'</td><td>mcmv-miR-m21-1</td><td>1</td><td>-12.8</td><td>3' GCCGAACTTGCACAGGGGATA 5'
||| |:||||:
5' ---------CGTCTTCCCTG- 3'</td></tr>
<tr><td>Cbfa2t3h:::NM_009824</td><td>mcmv-miR-M23-2</td><td>5</td><td>-20.4</td><td>3' GGCGAACTGGCTC-CGGGGGTA 5'
:||| || || |||:|||
5' -TGCTCC-CCCAGAGCCTCCA- 3'</td><td>mcmv-miR-m21-1</td><td>3</td><td>-19.56</td><td>3' GCCGAACTTGCACAGGGGATA 5'
||: |||||||:|
5' -GGTGCTCTTA-GTCCCCTGT 3'</td></tr>
<tr><td>Kdr:::NM_010612</td><td>mcmv-miR-M23-2</td><td>1</td><td>-20.3</td><td>3' GGCGAACTGGCTCCGGGGGTA 5'
|:|: |||||||
5' ------GGCTCC-GCCCCCA- 3'</td><td>mcmv-miR-m21-1</td><td>2</td><td>-24.7</td><td>3' GCC-GAACTTGCACAGGGGATA 5'
||| || |||||||:|||:
5' CGGACTCTTACGTGTCTCCTG- 3'</td></tr>
<tr><td>Il1r1:::NM_008362</td><td>mcmv-miR-M23-2</td><td>1</td><td>-20.2</td><td>3' GGCGAACTGGCTCCGGGGGTA 5'
| ||| ||| |||||||
5' -CTCTT-ACCCCAGCCCCCA- 3'</td><td>mcmv-miR-m21-1</td><td>1</td><td>-13.6</td><td>3' GCCGAACTTGCACAGGGGATA 5'
|||||:|:|
5' ------------GTCCCTTGT 3'</td></tr>
</table>

Supplementary Table 1. page 14

<table>
<tr><td>Bcl3:::NM_033601</td><td>mcmv-miR-M23-2</td><td>1</td><td>-19.9</td><td>3' GGCGAACTGGCTC-CGGGGGTA 5'
        |||: |||||||
5' ------GACTCTCAGCCCCCA- 3'</td><td>mcmv-miR-m21-1</td><td>1</td><td>-13.2</td><td>3' GCCGAACTTGCACAGGGGATA 5'
                 ||:||||:|
5' ------------GTTCCCTGT 3'</td></tr>
<tr><td>Ttc7:::NM_028639</td><td>mcmv-miR-M23-2</td><td>2</td><td>-19.6</td><td>3' GGCGAACTGGCTCCGGGGGTA 5'
    | ||||:|: ||||||
5' -C-CTTGGCTT---CCCCCA- 3'</td><td>mcmv-miR-m21-1</td><td>2</td><td>-16</td><td>3' GCCGAACTTGCAC-AGGGGATA 5'
              ||| ||||||:
5' ----------GTGCTCCCCTG- 3'</td></tr>
<tr><td>Prkca:::NM_011101</td><td>mcmv-miR-M23-2</td><td>1</td><td>-19.6</td><td>3' GGCGAACTGGCTCCGGGGGTA 5'
    :|| || |||||||
5' -TGCCT--CCCCAGCCCCCA- 3'</td><td>mcmv-miR-m21-1</td><td>3</td><td>-16.3</td><td>3' GCCGAACTTGCACAGGGGATA 5'
    || :|| ||||:||:
5' -GGA---GACAGGTCCTCTG- 3'</td></tr>
<tr><td>Slc7a2:::NM_001044740</td><td>mcmv-miR-M23-2</td><td>1</td><td>-19.5</td><td>3' GGCGAACTGGC---TCCGGGGGTA 5'
        |:| |||||:||:
5' ------GGCAGAATAGGCCTCCG- 3'</td><td>mcmv-miR-m21-1</td><td>2</td><td>-14.7</td><td>3' GCCGAACTTGCAC-AGGGGATA 5'
      || ||:| ||||||:|
5' ---CTGAAATGCCATCCCCTGT 3'</td></tr>
<tr><td>Ifnar1:::NM_010508</td><td>mcmv-miR-M23-2</td><td>1</td><td>-19.4</td><td>3' GGCGAACT-GGCTC----CGGGGGTA 5'
        || |:||| |:|||||
5' ------GATCTGAGCTCTGTCCCCA- 3'</td><td>mcmv-miR-m21-1</td><td>4</td><td>-17.8</td><td>3' GCCGAACTTGC-ACAGGGGATA 5'
           |||| |||||:||:|
5' ------GAACCCTGTCCTCTGT 3'</td></tr>
<tr><td>Nod2:::NM_145857</td><td>mcmv-miR-M23-2</td><td>3</td><td>-19.4</td><td>3' GGCGAAC-TGGCTCCGGGGGTA 5'
   || | ||| ||||||||
5' CCACCCCCACCCCCGCCCCCAT 3'</td><td>mcmv-miR-m21-1</td><td>1</td><td>-15.4</td><td>3' GCCGAACTTGCAC-AGGGGATA 5'
    ||: |:| ||||||:
5' -GGTCA-AGCAGATTCCCCTG- 3'</td></tr>
<tr><td>Chrna4:::NM_015730</td><td>mcmv-miR-M23-2</td><td>2</td><td>-19.3</td><td>3' GGCGAACTGGCTCCGGGGGTA 5'
    ||| ||| |||:|||
5' -CGC---ACCAA-GCCTCCA- 3'</td><td>mcmv-miR-m21-1</td><td>1</td><td>-16.37</td><td>3' GCCGAACTTGCACAGGGGATA 5'
    |||| ||||||
5' -GGCTACCCACTTCCCCCTA- 3'</td></tr>
<tr><td>B4galt1:::NM_022305</td><td>mcmv-miR-M23-2</td><td>1</td><td>-19</td><td>3' GGCGAACTGGCTCCGGGGGTA 5'
          |||| |||:|||
5' --------CCGA-GCCTCCA- 3'</td><td>mcmv-miR-m21-1</td><td>1</td><td>-13.4</td><td>3' GCCGAACTTGCACAGGGGATA 5'
                 |||||:|:|
5' ------------GTCCCTTGT 3'</td></tr>
<tr><td>Sox6:::NM_001025559</td><td>mcmv-miR-M23-2</td><td>5</td><td>-18.4</td><td>3' GGCGAACTGGCTCCGGGGGTA 5'
    |||| || ||||||
5' -CGCT---CCCTTTCCCCCA- 3'</td><td>mcmv-miR-m21-1</td><td>1</td><td>-19</td><td>3' GCCGAACTTGCACAGGGGATA 5'
   |||: |:|| ||||||:|
5' CGGT--GGAC---TCCCCTGT 3'</td></tr>
<tr><td>Bak1:::NM_007523</td><td>mcmv-miR-M23-2</td><td>3</td><td>-18.4</td><td>3' GGCGAACTGGCTCCGGGGGTA 5'
   |:|:   :| |||||||
5' CTGT---GCAAT-GCCCCCA- 3'</td><td>mcmv-miR-m21-1</td><td>1</td><td>-10.9</td><td>3' GCCGAACTTGCACAGGGGATA 5'
                  ||||||
5' --------------CCCCTA- 3'</td></tr>
<tr><td>Cebpg:::NM_009884</td><td>mcmv-miR-M23-2</td><td>1</td><td>-18.4</td><td>3' GGCGAACTGGCTC--CGGGGGTA 5'
       ||:|| |||:|||
5' -----TGGCCTTCCTGCCTCCA- 3'</td><td>mcmv-miR-m21-1</td><td>1</td><td>-12.3</td><td>3' GCCGAACTTGCACAGGGGATA 5'
    ||:| ||:|||:|
5' -GGTT--------TCTCCTGT 3'</td></tr>
<tr><td>Sla2:::NM_029983</td><td>mcmv-miR-M23-2</td><td>2</td><td>-18.3</td><td>3' GGCGAA--CTGGCTCC----GGGGGTA 5'
   |||| |||| |:||||
5' CCGCAGTAGACCCCACCTAACTCCCA- 3'</td><td>mcmv-miR-m21-1</td><td>1</td><td>-12.24</td><td>3' GCCGAACTTGCACAGGGGATA 5'
    ||| ||||||
5' -GGCCTCA------CCCCTA- 3'</td></tr>
<tr><td>Ndrg1:::NM_008681</td><td>mcmv-miR-M23-2</td><td>2</td><td>-17.7</td><td>3' GGCGAA-CTGGCTCCGGGGGTA 5'
    ||:| || : ||||:||
5' -CGTTGGGAGTCA-GCCCTCA- 3'</td><td>mcmv-miR-m21-1</td><td>1</td><td>-15.1</td><td>3' GCCGAACTTGCACAGGGGATA 5'
     || |||| ||||:|:||
5' --GC-TGAA--TGTCTCTTA- 3'</td></tr>
<tr><td>Skap1:::NM_001033186</td><td>mcmv-miR-M23-2</td><td>1</td><td>-17.4</td><td>3' GGCGAACT-GGCTCCGGGGGTA 5'
     || |:| |:|||||
5' --GCCCTCTCTGT-GTCCCCA- 3'</td><td>mcmv-miR-m21-1</td><td>1</td><td>-16.1</td><td>3' GCCGAACTTGCACAGGGGATA 5'
                ||||||||
5' ------------GTCCCCTA- 3'</td></tr>
<tr><td>Tnfsf12:::NM_011614</td><td>mcmv-miR-M23-2</td><td>2</td><td>-17.4</td><td>3' GGCGAACTGGCTCCGGGGGTA 5'
   || | ||||: ||||||
5' CC-C-TGACT----CCCCCA- 3'</td><td>mcmv-miR-m21-1</td><td>1</td><td>-10.7</td><td>3' GCCGAACTTGCACAGGGGATA 5'
      ||| ||| |:||||:
5' ---CTTAAACT--TTCCCTG- 3'</td></tr>
<tr><td>Kit:::NM_021099</td><td>mcmv-miR-M23-2</td><td>2</td><td>-17.4</td><td>3' GGCGAACTGGCTCCGGGGGTA 5'
    | |||| || ||||||
5' -C-CTTG-CCCT--CCCCCA- 3'</td><td>mcmv-miR-m21-1</td><td>1</td><td>-16.5</td><td>3' GCCGAACTTGCAC-AGGGGATA 5'
              ||| ||||||:
5' ----------GTGCTCCCCTG- 3'</td></tr>
<tr><td>Pbx1:::NM_183355</td><td>mcmv-miR-M23-2</td><td>3</td><td>-17.3</td><td>3' GGCGAACTGGCTC-CGGGGGTA 5'
   || | || :| ||||:|||
5' CCTC---ACTAGGAGCCCTCAT 3'</td><td>mcmv-miR-m21-1</td><td>1</td><td>-10.1</td><td>3' GCCGAACTTGCACAGGGGATA 5'
                  |:||||:|
5' -------------TTCCCTGT 3'</td></tr>
<tr><td>Twsg1:::NM_023053</td><td>mcmv-miR-M23-2</td><td>1</td><td>-17.1</td><td>3' GGCGAACTGGC--TCCGGGGGTA 5'
     || || :||:|:|||
5' --GCAGAACAGCAGGGTCTCCA- 3'</td><td>mcmv-miR-m21-1</td><td>1</td><td>-29.7</td><td>5'
    ||:|||:|:|| |||||||:|
5' -GGTTTGGATGTTATGTCCCCTGT</td></tr>
<tr><td>Il10:::NM_010548</td><td>mcmv-miR-M23-2</td><td>1</td><td>-16.9</td><td>3' GGCGAACTGGCTCC-GGGGGTA 5'
   || || ||:| ||||||
5' CC-CT--ACTGTCATCCCCCA- 3'</td><td>mcmv-miR-m21-1</td><td>1</td><td>-17.1</td><td>3' GCCGAACTTGCACAGGGGATA 5'
           ||: || |||||||
5' ------GAGGGT-TCCCCTA- 3'</td></tr>
<tr><td>Il25:::NM_080729</td><td>mcmv-miR-M23-2</td><td>1</td><td>-16.8</td><td>3' GGCGAACTGGCTCCGGGGGTA 5'
          |:|| ||||||
5' --------CTGAATCCCCCA- 3'</td><td>mcmv-miR-m21-1</td><td>1</td><td>-16.3</td><td>3' GCCGAACTTGCACAGGGGATA 5'
    |||| ||||||:
5' -GGCTCCTT----TCCCCTG- 3'</td></tr>
<tr><td>Syk:::NM_011518</td><td>mcmv-miR-M23-2</td><td>1</td><td>-16.8</td><td>3' GGCGA-ACTGGCTCCGGGGGTA 5'
     | ||||| :|||||
5' --GATGTGACCT---TCCCCA- 3'</td><td>mcmv-miR-m21-1</td><td>2</td><td>-22.52</td><td>3' GCCGAACTTGCACAGGGGATA 5'
    || |||||||||:
5' -GGATTTCA-GTGTCCCCTG- 3'</td></tr>
<tr><td>Cxcl14:::NM_019568</td><td>mcmv-miR-M23-2</td><td>2</td><td>-16.6</td><td>3' GGCGAACTGGCTCCGGGGGTA 5'
     || || |:|||||
5' --GCAT--CCAAAGTCCCCA- 3'</td><td>mcmv-miR-m21-1</td><td>2</td><td>-22.7</td><td>3' GCCGA--ACTTGCACAGGGGATA 5'
    ||:| |||| |||||||:
5' -GGTTTCTGAAAG-GTCCCCTG- 3'</td></tr>
<tr><td>Add1:::NM_001102444</td><td>mcmv-miR-M23-2</td><td>1</td><td>-16.6</td><td>3' GGCGAACTGGCTCCGGGGGTA 5'
        |:| |:||||||
5' ------GGCAA--GTCCCCAT 3'</td><td>mcmv-miR-m21-1</td><td>1</td><td>-12.7</td><td>3' GCCGAACTTGCACAGGGGATA 5'
          ||| | ||||||:
5' -----TGACCTC-TCCCCTG- 3'</td></tr>
<tr><td>Osm:::NM_001013365</td><td>mcmv-miR-M23-2</td><td>1</td><td>-16.5</td><td>3' GGC-GAACT--GGCTCCGGGGGTA 5'
     | ||||| |:| ||:|||
5' --GACTTGAAACTGTTTCCTCCA- 3'</td><td>mcmv-miR-m21-1</td><td>2</td><td>-24.7</td><td>5'
    || |||| |||||||||
5' -GGGGTGAAGTTGAGTGTCCCCTA-</td></tr>
<tr><td>Pik3cd:::NM_001029837</td><td>mcmv-miR-M23-2</td><td>1</td><td>-16.5</td><td>3' GGCGAACTGGCTC---CGGGGGTA 5'
           ||:| ||||:||
5' ---------CGGGAACGCCCTCA- 3'</td><td>mcmv-miR-m21-1</td><td>1</td><td>-13.9</td><td>3' GCCGAACTTGCACAGGGGATA 5'
           || : ||||||:||
5' ------GACTCTGTCCCTTA- 3'</td></tr>
<tr><td>Samhd1:::NM_018851</td><td>mcmv-miR-M23-2</td><td>1</td><td>-16.47</td><td>3' GGCGAACTGGCTCCGGGGGTA 5'
     ||| ||||||
5' --GCTGTAAGCACACCCCCA- 3'</td><td>mcmv-miR-m21-1</td><td>1</td><td>-17.4</td><td>5'
    ||: ||| ||||||||:
5' -GGTAC-AACTCCCCCTGTCCCCTG-</td></tr>
<tr><td>Slamf1:::NM_013730</td><td>mcmv-miR-M23-2</td><td>1</td><td>-16.4</td><td>3' GGCGAACTGGCTCC---------GGGGGTA 5'
             |||| ||||||
5' ----------GAGGAAGATACTCCCCCCA- 3'</td><td>mcmv-miR-m21-1</td><td>2</td><td>-22.2</td><td>5'
    ||| ||:|| |||||||:
5' -GGCATAAATGTCAAGTCCCCTG-</td></tr>
<tr><td>Oasl2:::NM_011854</td><td>mcmv-miR-M23-2</td><td>1</td><td>-15.8</td><td>3' GGCGAACTGGCTCC---GGGGGTA 5'
        |||:| :|||||
5' ------GACTGTCAGATTCCCCA- 3'</td><td>mcmv-miR-m21-1</td><td>1</td><td>-14.7</td><td>AGGGGATA 5'
        ||:||||
||||:|:|</td></tr>
<tr><td>Apc:::NM_007462</td><td>mcmv-miR-M23-2</td><td>1</td><td>-15.5</td><td>3' GGCGAACTGGCTCCGGGGGTA 5'
       ||| |:|||||
5' -----TGAAGTCTGTCCCCA- 3'</td><td>mcmv-miR-m21-1</td><td>4</td><td>-17.3</td><td>3' GCCGAACTTGCACAGGGGATA 5'
    || |||| ||||||
5' -GGAAAGAACCAGCCCCCTA- 3'</td></tr>
<tr><td>Rbpj:::NM_001080927</td><td>mcmv-miR-M23-2</td><td>1</td><td>-15.2</td><td>3' GGCGAACTGGCTCCGGGGGTA 5'
         |: ||| |||:||
5' -------ATGGAGCCCCTCA- 3'</td><td>mcmv-miR-m21-1</td><td>1</td><td>-19.8</td><td>3' GCCGAACTTGCACAGGGGATA 5'
     |||| || ||||||:||
5' --GCTTTTAC-TGTCCCTTA- 3'</td></tr>
<tr><td>Pml:::NM_008884</td><td>mcmv-miR-M23-2</td><td>3</td><td>-15.2</td><td>3' GGCGAACTGGCTCCGGGGGTA 5'
     ||| || ||:|||
5' --GCTAATCCCCACCCTCCA- 3'</td><td>mcmv-miR-m21-1</td><td>1</td><td>-16.4</td><td>3' GCCGAACTTGCACAGGGGATA 5'
    ||: | |||:||||:|
5' -GGTCAGT---TGTTCCCTGT 3'</td></tr>
<tr><td>Pik3r1:::NM_001024955</td><td>mcmv-miR-M23-2</td><td>1</td><td>-15.1</td><td>3' GGCGAACTGGCTCCGGGGGTA 5'
     |||| :| ||:|||
5' --GCTTTGCAA---CCTCCA- 3'</td><td>mcmv-miR-m21-1</td><td>1</td><td>-20.1</td><td>3' GCCGAACTTGCACAGGGGATA 5'
    |||| ||||:|||:|
5' -GGCTGTT---TGTCTCCTGT 3'</td></tr>
<tr><td>Cxcr4:::NM_009911</td><td>mcmv-miR-M23-2</td><td>1</td><td>-15</td><td>3' GGCGAACTGGCTCCGGGGGTA 5'
   || | ||| ||||||
5' CC-CCC-ACCCCCACCCCCA- 3'</td><td>mcmv-miR-m21-1</td><td>1</td><td>-10.3</td><td>3' GCCGAACTTGCACAGGGGATA 5'
        |||| :|||||
5' ----TTGATTTCACTCCCTA- 3'</td></tr>
</table>

Supplementary Table 1. page 15

<table>
<tr><td>Elf4:::NM_019680</td><td>mcmv-miR-M23-2</td><td>1</td><td>-14.7</td><td>3' GGCGA----ACTGGC-TCCGGGGGTA 5'
||| |||:| || |:|:||
5' --GCTCTCCTGATCTTAGTCTCTCA- 3'</td><td>mcmv-miR-m21-1</td><td>1</td><td>-11.6</td><td>3' GCCGAACTTGCACA-GGGGATA 5'
| ||| :|||||
5' ------GTCCGTCCCTCCCTA- 3'</td></tr>
<tr><td>Rsad2:::NM_021384</td><td>mcmv-miR-M23-2</td><td>1</td><td>-14.7</td><td>3' GGCGAAC-TGGCTCCGGGGGTA 5'
|| ||| ||||||
5' -----TGTACC----CCCCCA- 3'</td><td>mcmv-miR-m21-1</td><td>2</td><td>-17.2</td><td>3' GCCGAACTTGCACAGGGGATA 5'
||||| ||||||:|
5' -----TGAAC---TCCCCTGT 3'</td></tr>
<tr><td>C3ar1:::NM_009779</td><td>mcmv-miR-M23-2</td><td>1</td><td>-14.6</td><td>3' GGCGAACTGGCTCCGGGGGTA 5'
|| | || ||||||
5' CCACCAT-CCCCA-CCCCCA- 3'</td><td>mcmv-miR-m21-1</td><td>2</td><td>-15.4</td><td>3' GCCGAACTTGCACAGGGGATA 5'
|||:|| || |:||||||
5' CGGTTT--ACATATTCCCTAT 3'</td></tr>
<tr><td>Atp7a:::NM_009726</td><td>mcmv-miR-M23-2</td><td>1</td><td>-14.3</td><td>3' GGCGAAC-TGGC-TCCGGGGGTA 5'
| || || ||||||
5' ------GAACATAAGTCCCCCA- 3'</td><td>mcmv-miR-m21-1</td><td>1</td><td>-11.3</td><td>3' GCCGAACTTGCACAGGGGATA 5'
|| ||||::||
5' -----TGTCATTGTCCTTTA- 3'</td></tr>
<tr><td>Ifit2:::NM_008332</td><td>mcmv-miR-M23-2</td><td>1</td><td>-13.8</td><td>3' GGCGAACTGG--CTC-CGGGGGTA 5'
|| || ||||:||
5' ------GAAAAAGAAAGCCCTCA- 3'</td><td>mcmv-miR-m21-1</td><td>1</td><td>-14.2</td><td>3' GCCGA-ACTTGCACAGGGGATA 5'
||| ||: ||||||:
5' --GCTCTGGT----TCCCCTG- 3'</td></tr>
<tr><td>Rag1:::NM_009019</td><td>mcmv-miR-M23-2</td><td>2</td><td>-13.4</td><td>3' GGCGAACTGGCTCCGGGGGTA 5'
|| ||||||
5' --------CCCACTCCCCCA- 3'</td><td>mcmv-miR-m21-1</td><td>1</td><td>-12.1</td><td>3' GCCGAACTTGCACAGGGGATA 5'
||||||:
5' ------------TCCCCTG- 3'</td></tr>
<tr><td>Ciapin1:::NM_134141</td><td>mcmv-miR-M23-2</td><td>1</td><td>-13.22</td><td>3' GGCGAACTGGCTCC-GGGGGTA 5'
|| ||||||
5' ------GAAGTCACTCCCCCA- 3'</td><td>mcmv-miR-m21-1</td><td>1</td><td>-12.5</td><td>3' GCCGAACTTGCACAGGGGATA 5'
||||||:|
5' ------------TCCCCTGT 3'</td></tr>
<tr><td>Sod2:::NM_013671</td><td>mcmv-miR-M23-2</td><td>1</td><td>-12.3</td><td>3' GGCGAACTGGCTCCGGGGGTA 5'
|| || ||| :|||||
5' CC-CT--ACC----TCCCCA- 3'</td><td>mcmv-miR-m21-1</td><td>1</td><td>-12.4</td><td>3' GCCGAACTTGCACAGGGGATA 5'
|||||||
5' ------------TCCCCTA- 3'</td></tr>
<tr><td>Myd88:::NM_010851</td><td>mcmv-miR-M23-2</td><td>1</td><td>-12.1</td><td>3' GGCGAAC-TGGCTCCGGGGGTA 5'
|: ||| ||:|||
5' --GTGTAAACCT---CCTCCA- 3'</td><td>mcmv-miR-m21-1</td><td>1</td><td>-12.2</td><td>3' GCCGAACTTGCACAGGGGATA 5'
|||||||
5' ------------TCCCCTA- 3'</td></tr>
<tr><td>Hoxb3:::NM_001079869</td><td>mcmv-miR-M23-2</td><td>1</td><td>-10.4</td><td>3' GGCGAACTGGCTCCGGGGGTA 5'
|| ||:|||
5' --------CCACTCCCTCCA- 3'</td><td>mcmv-miR-m21-1</td><td>1</td><td>-10.9</td><td>3' GCCGAACTTGCACAGGGGATA 5'
||||||
5' -------------CCCCTA- 3'</td></tr>
<tr><td>Cxcl12:::NM_001012477</td><td>mcmv-miR-M23-2</td><td>1</td><td>-10.4</td><td>3' GGCGAACTGGCTCCGGGGGTA 5'
|| | || |||:||
5' CC-CAAT-CCACTACCCTCA- 3'</td><td>mcmv-miR-m21-1</td><td>3</td><td>-17.8</td><td>3' GCCGAACTTGCACAGGGGATA 5'
||||| :| |:||||:
5' CGGCTGT-GCCC-TTCCCTG- 3'</td></tr>
</table>
