# Supplementary material for: Cytomegalovirus microRNAs Facilitate Persistent Virus Infection in Salivary Glands
Source: PLoS Pathog. 2010 Oct 14;6(10):e1001150. doi: 10.1371/journal.ppat.1001150 (PMC2954898; doi:10.1371/journal.ppat.1001150)
Supplement: Table S2 — List of PCR primers and oligonucleotides. (0.23 MB PDF) [file ppat.1001150.s007.pdf]

Table S2: PCR Primers & Oligonucleotides

Primers & Oligos Sequence

H5-miR-M23-2-galK/Kn 5’-**TCCCGGTTTCTTTGTCCGACGCTCGTCGATCGAGGCGCTA**GCCAGTGTTACAACCAATTAACC
H3-miR-M23-2-galk/Kn 5’-**CAGATAGACAGACAGGCTCAGTCTCATACCGTCGGCCATC**CCTGTTGACAATTAATCATCGGCA-3’
Homologies to the MCMV genome are indicated in bold.

H5-miR-M23-rev: 5’-CGACAGAGAAGAGTCCCGACACCC-3’
H3-miR-M23-rev: 5’-TCACCGCCTGCCTTCCTGTTCA-3’.
H5-GalK/Kn-m22: 5’-
GACAAACAGACAGATAGACAGACAGGCTCAGTCTCATACCGTCGGCCATCCCTGTTGACAATTAATCATCGGCA-3’;
H3-GalK/Kn-m22: 5’-
ATCGGATCGGACGGACCGGACGGACCGCGACTGCTTGTCGGGCGGGTGTGCCAGTGTTACAACCAATTAACC-3’
Homologies to the MCMV genome are indicated in bold.

m22-for: 5’-GTGTGAGCTC*GATATC*CCGCCCGAATTCGCAGCAG-3’;
m22-rev: 5’- GTGT**GGTACC***GATATC*CGCTCGAGGCCATGCTC-3’.
SacI site undelined, KpnI site indicated in bold, EcoRV sites indicated in italics

pre-miR-M23-2-mut-s: 5’-TGCAC**T**CG**G**AG**C**AA**G**CT**T**CC**T**CC**T**AT**T**GG**C**GA**G**AC**A**TT**T**AA**C**CC**T**GG**A**TA**C**CG**T**CTCGAT-3’;
pre-miR-M23-2-mut-as: 5’-CGATCGAG**A**CG**G**TA**T**CC**A**GG**G**TT**A**AA**T**GT**C**TC**G**CC**A**AT**A**GG**A**GG**A**AG**C**TT**G**CT**C**CG**A**GTGCAC-3’.
Mutated nucleotides are indicated in bold.

PM-M23-2-for 5’-AAAAAGCAGGCTATCGCCACCTTGTTTAAGCCACCGCTTGACCGAGGCCCCCATATTAGACCTACGCACTCCAG-3'
PM-M23-2-rev 5’-
AGAAAGCTGGGTCTGGAGTGCGTAGGTCTAATATGGGGGCCTCGGTCAAGCGGTGGCTTAAACAAGGTGGCGAT-3'
MM-M23-2-for 5’ AAAAAGCAGGCTATCGCCACCTTGTTTAAGCCACCGCTTGACCCTCGCCCCCATATTAGACCTACGCACTCCAG-3’
MM-M23-2-rev 5’-
AGAAAGCTGGGTCTGGAGTGCGTAGGTCTAATATGGGGGCGAGGGTCAAGCGGTGGCTTAAACAAGGTGGCGAT-3’

CXCL16-for 5’-AAAAAGCAGGCTTGGAAGCTCATGAGAGATGG-3’
CXCL16-rev. 5’-AGAAAGCTGGGTCTGGTAGTGGGAGGGTTGAA-3’
AttB1-for 5’-GGGGACAAGTTTGTACAAAAAAGCAGGCT-3’
AttB2-rev 5’ GGGGACCACTTTGTACAAGAAAGCTGGGT-3’

m21for 5’- GCAGCAGGCGACAGAGAAGA -3’
m21rev 5’- CCATCTGCCTGAGTTTCGCTC -3'
M23for 5’- ACAGCAGAGAAGAGCGGCGA -3'
M23rev5’- TCTCGCTCTGCATCGGCAT -3'
